# Seasonal Dynamics of Lake Winnipeg’s Microbial Communities Reveal Aerobic Anoxygenic Phototrophic Populations Coincide with Sunlight Availability

**DOI:** 10.3390/microorganisms10091690

**Published:** 2022-08-23

**Authors:** Steven B. Kuzyk, Xiao Ma, Vladimir Yurkov

**Affiliations:** Department of Microbiology, University of Manitoba, Winnipeg, MB R3T 2N2, Canada

**Keywords:** microbial ecology of lakes, aerobic anoxygenic phototrophs, bacterial community, Lake Winnipeg, food web dynamics, picoplankton, bacterioplankton

## Abstract

In this first comprehensive study of Lake Winnipeg’s microbial communities, limnetic and littoral euphotic zones were examined during each season from 2016 through 2020. Classical cultivation and modern high-throughput sequencing techniques provided quantification and identification of key phototrophic populations, including aerobic anoxygenic phototrophs (AAP). Annual dynamics found total heterotrophs reached 4.23 × 10^6^ CFU/g in littoral sands, and 7.69 × 10^4^ CFU/mL in summer littoral waters on oligotrophic media, higher counts than for copiotrophic compositions. Limnetic numbers inversely dipped to 4.34 × 10^3^ CFU/mL midsummer. Cultured AAP did not follow heterotrophic trends, instead peaking during the spring in both littoral and limnetic waters as 19.1 and 4.7% of total copiotrophs, or 3.9 and 4.9% of oligotrophs, decreasing till autumn each year. Complementary observations came from environmental 16S V4 rRNA gene analysis, as AAP made up 1.49 and 1.02% of the littoral and limnetic sequenced communities in the spring, declining with seasonal progression. Spatial and temporal fluctuations of microbes compared to environmental factors exposed photosynthetic populations to independently and regularly fluctuate in the ecosystem. Oxygenic phototrophic numbers expectantly matched the midsummer peak of Chl *a* and *b*, oxygenic photosynthesis related carbon fixation, and water temperature. Independently, AAP particularly colonized spring littoral areas more than limnetic, and directly corresponded to habitat conditions that specifically promoted growth: the requirement of light and organic material.

## 1. Introduction

Lakes are the largest accumulations of liquid freshwater on Earth yet make up only 1% of the world’s surface. These habitats have maintained incredible importance as sources for fisheries, drinking water and irrigation supply. The North American continent contains many of the highest volume natural water reservoirs, where the detrimental effects of anthropogenic eutrophication have caused the Laurentian Great Lake ecosystems to be rigorously monitored [1,2,3,4]. In contrast, the isolated northern Great Bear, Great Slave, and Lake Winnipeg have received less attention [5,6,7]. Of these three, Great Bear Lake has incurred the least human exposure, and Great Slave similarly maintained low land usage within its watershed. Inversely, Lake Winnipeg has been subjected to considerable nutrient loading due to its massive drainage basin spanning over 1,000,000 km^2^ across every Canadian prairie province and some American states [8]. All significant agricultural, industrial, municipal, and urban runoff eventually enters its waters prior to draining into the Hudson’s Bay of the Arctic Ocean via a single outlet, the Nelson River. In response, studies have predominantly focused on chemical and environmental fluctuations, as well as the impact to fish populations and other eukaryotes, presented thoroughly in recent collaborative efforts by academic researchers and provincial and federal governments [9,10]. Nonetheless, some knowledge gaps have remained, including a lack of information regarding the primary producers, consumers, and recyclers of nutrients and biomass. The organisms at the base of food web dynamics, the microbial community, were thus far largely unexplored.

Algae and cyanobacteria have been commonly characterized in freshwaters, acting as aerobic oxygenic photoautotrophs (AOP) that use chlorophyll (Chl)-based photosynthesis, capable of fixing CO_2_ into organic compounds consumable throughout the food chain [11,12]. However, as published for other great lakes [13], or in our cursory study of Lake Winnipeg [14,15], AOP made up only a portion of the microbial community, where many other photoheterotrophs, photoautotrophs, chemoheterotrophs, and chemoautotrophs also inhabited and influenced the ecosystem. Phototrophic populations have been particularly diverse in aquatic habitats, with predominant groups differentiated by their photosynthetic systems. Anaerobic anoxygenic phototrophs (AnAnP) produce biological energy from light using bacteriochlorophyll (BChl) mediated photophosphorylation in the absence of oxygen, typical of stratified or benthic zones [16]. Aerobic anoxygenic phototrophs (AAP) similarly use BChl *a* as a primary pigment, but require oxic euphotic habitats rather than anoxic ones. Photosynthesis has likely evolved in AAP to act as an auxiliary energy production strategy to outcompete typical cohabitating heterotrophs by consuming organics faster due to the supplemental use of light energy [17,18]. In addition to assorted photosynthesizing populations, numerous other trophic groups co-exist with harmonious and complex interactions occurring on the microscopic scale in each ecosystem [19,20]. 

Regarding aquatic microbial ecology, many methods have been developed to study either individual populations or entire communities, uncovering a wide spectrum in microbial species composition among freshwater systems [13]. While variability could indeed be present, results have been difficult to compare due to dissimilar sampling methods/approaches. Furthermore, limited sampling locations and infrequent return visits have yet to address spatial and temporal system dynamics and stabilities [1,2,3,4]. To determine such ecosystem patterns, Lake Winnipeg’s microbial communities were investigated at multiple euphotic zones, over all four seasons during the past 5 years. Chosen limnetic and littoral sites were analyzed with classical microbial cultivation and modern sequencing techniques in addition to physico-chemical parameter collection, a crucial combination which allowed for both diversity and functionality to be monitored in unison. Among the phototrophic groups, AAP were specifically regarded as they have been discovered in a vast array of environments at high numbers [17,20,21,22]. However, their role in ecology and biogeochemical cycling of elements has remained unclear. The abundance, fluctuations, and relation of AAP to other populations of the community were therefore highlighted in this work. 

## 2. Materials and Methods

### 2.1. Sampling and Environmental Parameters

**Figure 1:**
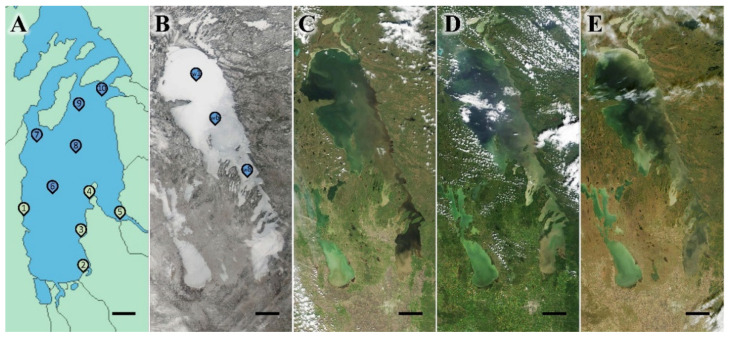
Lake Winnipeg seasonal progression and sampling locations. (**A**) Map of the South Basin of Lake Winnipeg, with littoral (green) and limnetic (blue) sites selected for study: S1–5, S6–10 (W1, W6, and W8 alternatively), respectively; Scale bar, 10 km. Satellite images of entire waterbody depicting seasonal change to lake habitat and surrounding lands during (**B**) Winter, 26 March 2017 indicating additional under-ice sites, (**C**) Spring, 31 May 2017, (**D**) Summer, 3 August 2017, and (**E**) Fall, 11 October 2017; Scale bars, 40 km. Photographs provided by Dr. K. Scott and the Lake Winnipeg Research Consortium (LWRC), in partnership with the Rapid Response Imagery from the Land Atmosphere Near-real time Capability for EOS (LANCE) system operated by the NASA/GSFC/Earth Science Data and Information System (ESDIS).

Lake Winnipeg was visited during the spring in May-June, summer between July-August, fall amid September-October, and winter among February-March, with 4 years’ worth of seasons occurring from 2016 to 2020 (Table S1). Euphotic regions were sampled 0.25 m below the surface at 5 littoral areas accessed by land, and 5 limnetic zones retrieved using a rosette canister deployed off the research vessel M.V. *Namao* during open-water months (Figure 1A), or from helicopter transport and ice coring equipment when the lake was frozen (Figure 1B). Each littoral site was located where lake water was 0.5 m deep, light reached the bottom, and had sediment samples collected. 

Littoral areas included Site 1 at Gimli Beach (50°38′2″ N, 96°59′2″ W); Site 2, Patricia Beach (50°25′25″ N, 96°37′2″ W); Site 3, Grand Beach (50°34′11″ N 96°35′59″ W); Site 4, Victoria Beach (50°42′16″ N, 96°33′49″ W); and Site 5 near the Sagkeeng First Nation Community (50°37′11″ N, 96°18′55″ W). Limnetic locations were spread across the South Basin, chosen from the expansive list that the M.V. *Namao* frequently visited, denoted here as Sites 6–10, with the catalog names provided in quotations. Site 6 “W10” (50°50.799′ N, 96°46.157′ W), Site 7 “57B” (50°58.529′ N, 96°52.551′ W), Site 8 “W9” (51°01.334′ N, 96°35.038′ W), Site 9 “12B” (51°08.014′ N, 96°37.159′ W), and Site 10 “46S” (51°08.512′ N, 96°25.270′ W) were all kilometers apart from each other as well as from the nearest shoreline. Only Sites 6 and 8 could be sampled in the winter, and during 2019–2020, 3 additional sites were included, W1 (53°22′34.788″, −98°23′25.188″), W6 (52°38′34.8″, −97°44′5.1″), and W8 (51°45′47.4474″, −96°50′21.6954″). While Sites 1–4, 6, and 7 were primarily fed by the waters of the Red River, Site 5 was downstream of the Winnipeg River, and 8–10 likely contained a mixture of the two inflows. Since Lake Winnipeg predominantly had a soft lakebed [23], sandy-silty shorelines were common.

Water temperature and pH were recorded with a calibrated Beckman Φ255 pH/Temp/mV meter, clarity by Secchi disk test, and light intensity (Lux) using a VWR Light Meter 21800-014. Data for daily average ambient temperatures, cloud coverage, and hours of sunlight were received from government sources. Major elemental compositions of limnetic water were determined via collision/reaction cell-based inductively coupled plasma mass spectrometry (CRC ICPMS) [15]. All 43 elements and their detection limits were collected by the Water Quality Management Section of Manitoba Environment, Climate and Parks Department, Canada and averages per season listed in Table S2. Concentrations of Chl *a*, Chl *b*, and BChl *a* from microbial communities were quantified as follows: A defined volume of liquid sample from each site was filtered through a 0.22 μm pore size 47 mm nitrocellulose membrane (Millipore-Sigma, Burlington, MA, USA) to concentrate cells, prior to the addition of 7:2 acetone:methanol (5 mL) for the extraction of pigments overnight in the dark at 4 °C. A Hitachi U-2010 spectrophotometer recorded absorption spectra from 350 to 1100 nm using a 50 mm path length quartz cuvette. Peaks at 663, 645, and 770 nm were used to calculate the concentration of Chl *a*, Chl *b*, and BChl *a,* respectively, per volume of water sample [24], based on classical approaches [25,26].

### 2.2. Primary Productivity Assay

Community photo- and chemosynthetic carbon fixation was determined during the summer and fall of 2017, as well as the spring, summer, and fall of 2018 and 2019 by a ^14^C-labelled NaHCO_3_ accumulation assay [27], with some minor procedural modifications [21]. Winter in situ analysis was not possible due to the extreme cold temperatures, where shallow littoral areas were frozen solid, and limnetic zones were not accessible twice during the same day. Measurement involved a 3 to 24 h in situ incubation of 3 sealed glass Balch vials each containing 9 mL of natural lake water and 5 µCi ^14^C-labelled NaHCO_3_. The first of three translucent vials contained only the two components, while the second was additionally wrapped in aluminum foil. The third uncovered vial contained the two components listed as well as 7 µmol diuron, a photosystem II (PSII) inhibitor. After residing in the lake, 400 µL of 40% formaldehyde (final as ~2%) was added, stopping reactions and “fixing” them for storage. A 0.1 mL aliquot was later filtered through a 0.22 μm pore size filter (Millipore-Sigma, Burlington, MA, USA) until dry, prior to washing off unfixed ^14^C with 5 mL of distilled water. Filters were placed in 0.5 mL ScintiSafe Plus 50% (Fisher Scientific, Waltham, MA, USA) scintillation fluid and measured for 5 min in a Beckman LS 6500. Counts were compared to 0.1 mL of unfiltered liquid for each sample. Carbonates and bicarbonates were determined by titration via a total alkalinity test [28]. Rates of carbon fixation were calculated and equalized to a 24 h incubation using the equation:

(1) $$\mathrm{Ca}=\frac{r\cdot C_{\mathrm{in}\;\mathrm{water}}}{R\cdot t}$$

Here, Ca was the amount of fixed carbon (mg/L); C_in water_, the total carbon present in water; R, the counts in control (unfiltered liquid sample); r, was counts in filtered material; and t, time of incubation. Chemosynthesis, anoxygenic or oxygenic photosynthesis related carbon fixation were calculated as the rates of vial 2, vial 3 minus 2, or vial 1 minus 3, respectively [21].

### 2.3. Cultivation and Enumeration of Targeted Populations

Water and sediment samples were decimally diluted to 10^−7^ and 0.1 mL aliquots were spread in triplicates on 2% agar plates. An assortment of media was designed (Table S3) to isolate and enumerate several specific physiological groups of microorganisms, with a main focus on phototrophs. Heterotrophs, including photoheterotrophic AAP, were cultivated with a standard rich organic (RO) medium [14,29]. Oligotrophs were selected on an organic-limited medium (OM), which had all compounds as RO, with only 10% of the carbon sources. A comparable R2A composition has been successful for the cultivation of some AAP [30], where recent works have diluted its contents to improve isolation attempts [31,32]. With such considerations, RO was implemented as a copiotrophic carbon source, OM as oligotrophic, whereas R2A was an intermediate with carbon content between RO and OM. Potato-broth based medium (PM) was included as it usually stimulated intense cellular pigmentation as a very rich nutrient source [17], and helped to discover rare colony colorations. Purple non-sulfur bacteria and other AnAnP were enriched on a specific medium, PNSM, while AOP were selected on BG-11 [21]. Heterotrophs capable of resisting toxic metalloid oxides were counted on RO supplemented with 100 µg/mL K_2_TeO_3_ (RO_T_), as some AAP have been found resistant to high levels [15,33,34]. Media were autoclaved at pH 5.9 and adjusted to 7.5 before agar solidification. Cultures were incubated for 7 days at 28 °C on RO, PM, R2A, and RO_T_ in the dark, and BG-11 and PNSM, under constant illumination. All were grown aerobically, except cultures on PNSM which developed in translucent oxygen-limited Gas-Pak chambers.

Pigmented and total (including pigmented and colorless) colony forming units (CFU) were counted as CFU/g of sediments, and CFU/mL in water. To determine numbers of AAP, numerous colored colonies were re-plated onto medium of isolation to verify growth and pigmentation, except colonies from RO_T_, which were instead passaged onto RO [15]. Patched strains were later suspended in 0.2 mM TRIS-HCl buffer, pH 7.8 containing 70% glycerol to measure absorption spectra from 350 to 1100 nm using a HITACHI U2010 spectrophotometer. Strict aerobes containing BChl *a* bound to reaction center and light harvesting complexes were identified as AAP [17]. The numerical presence of AAP among pigmented heterotrophs was established (Table S4), and used to estimate their proportion among the total cultured copiotrophic or oligotrophic heterotrophic bacteria, on RO and OM, respectively [15].

### 2.4. Microbial Community eDNA Sequencing

Environmental DNA (eDNA) was extracted from liquid or sediment samples using a QIAGEN DNeasy^®^ PowerWater^®^ Kit with minor modifications, and bacterial 16S V4 rRNA genes were sequenced by MiSeq (Illumina) with primer set 515F, 806R [15]. Community structures were computationally analyzed with Qiime2 11.3, a Python based framework [35,36], and BIOM formatting [37]. Reads were joined via paired-ends, trimmed to 292 bp (kept > 99%), then deblurred [38] and denoised [39], prior to de novo clustering sequences by 99% identity. Operational “taxonomic” unit (OTU) alignment were applied using 99% homology [40,41] to the Silva132 database [42], with a final data set that included “borderline-chimeras”. Phenetic identities were aligned as feature tables [43] at all hierarchical levels of the phylogenetic tree [44,45]. Both α- and β-diversity were statistically quantified, and graphed as boxplots or principle coordinate analysis (PCoA) [46,47]. Qiime2 project code text file describing entire pipeline and final output in both Qiime2 and BIOM formats were provided as Supplemental Materials. The AOP, AAP, and AnAnP proportions of the sequenced communities were analyzed by grouping all representative OTUs to known type species, Tables S5 and S6.

## 3. Results

### 3.1. Environmental Characteristics

**Figure 2:**
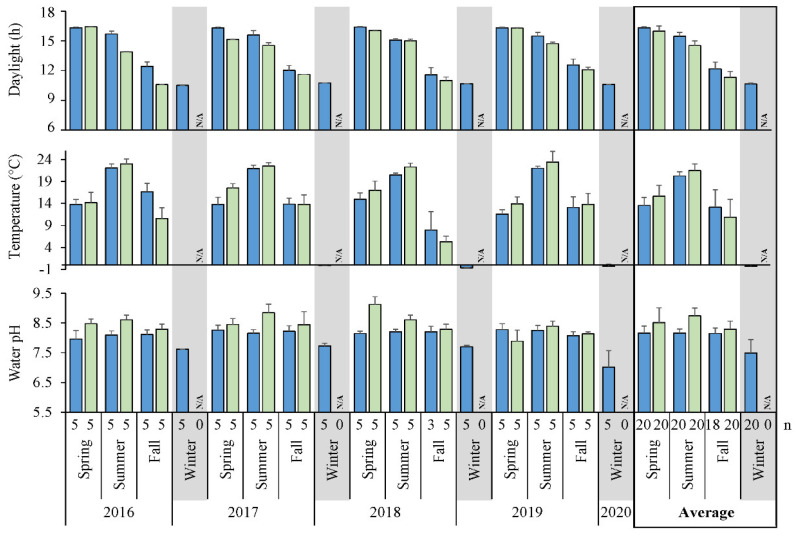
Periodic fluctuations of lake environmental parameters. Average light availability, water temperature and pH for all limnetic (blue bars) or littoral (light green bars) samples depicted for each season. N/A, not analyzed; n, number of samples per season.

**Figure A1:**
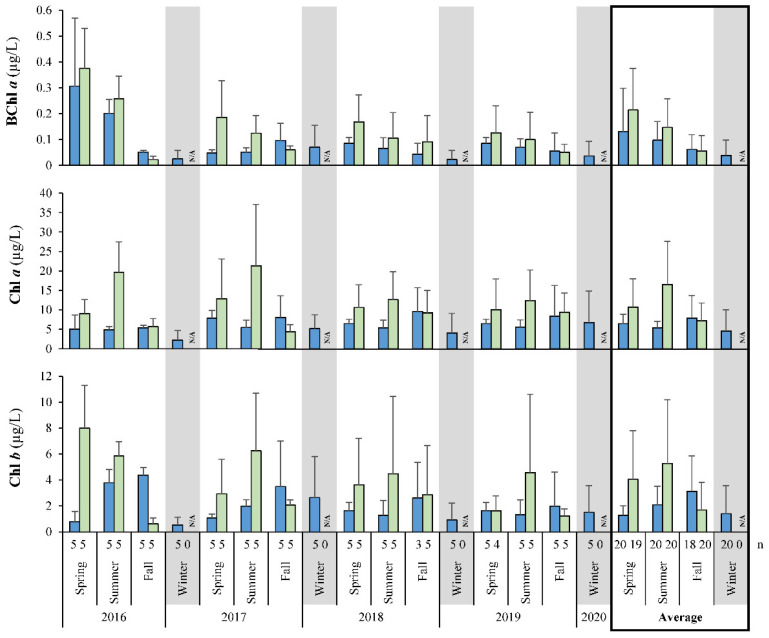
Photosynthesis primary pigment levels from each sampling location. Solvent extracts of collected biomass revealed BChl *a*, Chl *a*, and Chl *b* concentrations at limnetic (blue) or littoral (light green) zones. N/A, not analyzed; n, number of samples per season.

Seasonal observations of sites over 4 years (Table S1) revealed trends in physico-chemical environmental factors (Figure 2). Daylight was the longest during the spring with marginal variation, shortening into the fall (Figure 2). In comparison, lake water did not warm up until the peak of summer, decreasing to near 0 °C just below the ice surface at limnetic zones during winter. Littoral areas reached an average high of 21.5 °C, with some sites up to 26.0 °C, while limnetic regions had a moderate peak around 20.2 °C. Littoral warmed faster in the spring and summer, but was also colder than limnetic in the fall, which represented heat retention of open water. General trends in pH also followed seasonal changes, with winter limnetic zones being the most neutral at ~pH 7.5, whereas an increased pH ~8.2 was maintained at any other time. Littoral sites had a tendency to reach a slightly higher pH 8.5–8.7 in spring to summer, prior to dropping in the fall and holding a value similar to that offshore. Of the 43 elements estimated down to ppb in limnetic regions, most were invariably low (Table S2). Considering metals and metal (oids), only Al and Fe were above 1 ppm, where all others were closer to ppb concentrations. Extracted photosynthesis pigments Chl *a* and *b* had highest concentrations during the summer of most years, up to 16.5 and 5.3 μg/L on average, respectively (Figure A1). In comparison, levels of BChl *a* were lower and reached an average peak of 0.2 μg/L during the spring and decreased seasonally.

### 3.2. Primary Productivity Rates

**Figure 3:**
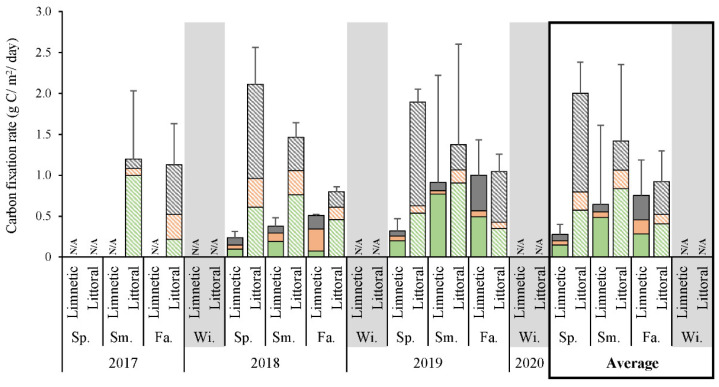
Primary productivity of Lake Winnipeg. ^14^C fixation measured in situ with total rates composed of oxygenic (green) or anoxygenic (orange) photosynthesis related fixation, and from dark chemosynthetic reactions (grey bars). N/A, not analyzed; Sp., Spring; Sm., Summer; Fa., Fall; Wi., Winter.

Total carbon fixation observed in situ at littoral sites between 2017 and 2019 had a repeatable yearly tendency for highest rates during the spring averaging 2.00 g C/m^2^/day, diminishing throughout the summer to become 0.92 g C/m^2^/day by the fall (Figure 3). Limnetic zones maintained a different pattern, where averaged offshore rates were at a minimum in the spring (0.28 g C/m^2^/day), gradually increasing throughout the summer to reach a fall maximum of merely 0.75 g C/m^2^/day. Independently, oxygenic phototrophs had a notable peak in fixation during the summer regardless of location (0.48–0.84 g C/m^2^/day), with reduced rates in both spring (0.15–0.57) and fall (0.28–0.40). When considering zones, shorelines maintained higher rates than offshore. Anoxygenic photosynthesis derived fixation was exceptionally low across all limnetic areas (0.05–0.17 g C/m^2^/day), and was only marginally higher in the littoral regions (0.11–0.23 g C/m^2^/day). Limnetic dark reactions (chemosynthesis) increased slightly from spring through fall, 0.08 to 0.30 g C/m^2^/day, respectively, whereas littoral zones had the highest rates in the spring (1.21 g C/m^2^/day) that would later decrease, with some annual inconsistency (Figure 3).

### 3.3. Seasonal Fluctuations in Cultivated Microbial Counts

**Figure 4:**
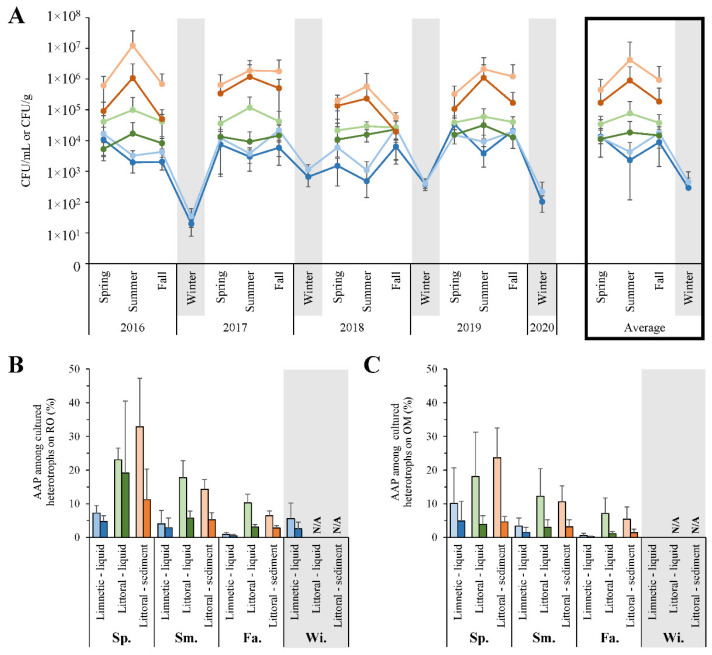
Enumeration of heterotrophs and AAP through all seasons and years. (**A**) Counts of total cultured heterotrophs from limnetic water (blue), littoral water (green), or sediment (orange) samples on copiotrophic RO (dark colors) or oligotrophic OM (lighter shade of each). (**B**) Copiotrophic and (**C**) oligotrophic AAP proportions of pigmented colonies (light shade) and total counts (dark shade) of seasonal averages from 2016–2020. N/A, not analyzed; Sp., Spring; Sm., Summer; Fa., Fall; Wi., Winter.

Total cultured bacteria on varied media were listed (Table S4), with patterns in RO grown copiotrophs and oligotrophs on OM evident after repetitive enumeration (Figure 4). Specifically, when grouped by zone and sample type for each season, littoral cultured bacteria peaked during the summer, while limnetic counts decreased at the same period (Figure 4A). Overall, littoral sediments maintained the highest numbers on OM (4.23 × 10^6^ CFU/g), shoreline waters had 100× less (7.69 × 10^4^ CFU/mL), and offshore counts were a further 10× decreased (4.34 × 10^3^ CFU/mL). As a general trend, more bacteria were culturable on OM than RO, regardless of season or site. Furthermore, PM and R2A media used as alternative complex organic sources to cultivate heterotrophs also maintained lesser counts than OM (Table S4).

AOP grown on BG-11 had a general apex during the summer of most years, to a maximum average of 9.37 × 10^3^ CFU/g and 4.13 × 10^3^ CFU/mL in littoral sediment and water, respectively, and would typically only reach a peak of 1.08 × 10^2^ CFU/mL offshore (Table S4). Anaerobes were often below detection limits and AnAnP hovered around 5.57 × 10^1^ CFU/mL when present. In comparison, AAP isolated and counted from both RO and OM displayed trends independent of summer maximal heterotroph numbers (Figure 4B, C). The most AAP were detected in the spring of each year on either media, diminishing into the fall. The proportion of the pigmented bacteria in littoral locations reached a height of 32.9% on RO, at which time AAP also made up to 4.7, 19.1, and 11.2% of the entire cultured community of limnetic waters, littoral liquid, and sediments, respectively (Figure 4B). In general, these photoheterotrophs were cultivated in highest numbers on OM in comparison to 10× nutrient enhanced RO or even higher carbon containing PM (Figure S1). They reached a peak of 2.39 × 10^4^ CFU/g in the spring littoral sediments. However, AAP made up a larger proportion of the heterotrophic numbers on RO, and PM, than they did on OM regardless of sampling site (Figure 4B,C, Table S3). Furthermore, RO_T_ was the most selective medium as 47.5% of cultured bacteria in sediments challenged with 100 µg/mL tellurite were AAP.

### 3.4. Sequenced Microbial Communities

**Figure 5:**
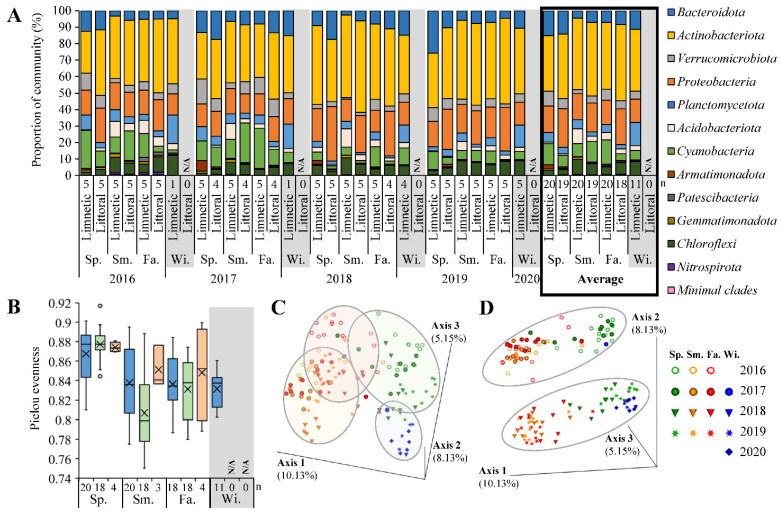
Bacterial community composition based on sequenced 16S V4 rRNA genes. (**A**) Phyla in water samples defined at limnetic vs. littoral sites. Minimal clades represented less than 1% per community with Firmicutes, Deinococcota, Bdellovibrionota, Crenarchaeota, Desulfobacterota, Hydrogenedentes, Margulisbacteria, Myxococcota, Sumerlaeota, SAR324, MBNT15, and NB1-j clades. (**B**) Total α-diversity of bacteria from limnetic water (blue), littoral water (green), or sediments (orange). (**C**,**D**) PCoA ordination based on Jaccard diversity distance matrix with percent variation as principal coordinates split on indicated axis. (**C**) From above axis 1 and 3 separate seasons, while (**D**) depicts major shift occurring along axis 2 by year with ellipses as visual cues. N/A, not analyzed; n, number of samples per season; Sp., Spring; Sm., Summer; Fa., Fall; Wi., Winter.

Amplified DNA resulted in an average of 261,105 reads from each site. After trimming and binning, 21,877 features representing unique phylotypes were identified throughout, later distinguished via Silva132 database to 2266 taxa of species level or higher. All samples were similarly rarified when grouped by season (Figure S2A), where the year 2016 reached a plateau of the highest diversity followed by 2017, then 2018–2020 (Figure S2B). The dominant phyla detected were averaged by zone for each collection period (Figure 5A). Considering a generalized community structure of Lake Winnipeg, the mean phyla across all sites and seasons listed from major to minor were 40.9 ± 10.4% Actinobacteriota, 17.5 ± 6.6% Proteobacteria, 9.9 ± 6.1% Bacteroidota, 9.8 ± 10.2% Cyanobacteria, 5.7 ± 4.0% Verrucomicrobiota, 5.7 ± 3.6% Chloroflexi, 4.9 ± 3.9% Planctomycetota, 3.4 ± 3.1% Acidobacteriota, 0.8 ± 0.6% Gemmatimonadota, 0.7 ± 1.6% Armatimonadota, 0.3 ± 0.7% Patescibacteria, 0.2 ± 0.5% Nitrospirota, and 0.3 ± 0.5% minimal clades. Standard deviation revealed some phyla maintained stability in the lake, while others varied dependent on location and time. Community richness and collation were exemplified using a PCoA of differing Jaccard beta diversity (Figure 5C,D), where samples were found to group by season (Figure 5C). A shift in composition was also detected from 2016 and 2017 to 2018 through 2020 (Figure 5D). The limnetic and littoral bacteria were also differentiated as separate cohorts when visualized in PCoA (Figure S3). Offshore samples were found at the extremities of the plot in tight groups, suggesting more stable communities. In comparison, shoreline representatives were both more centralized and dispersed, inferring a broad range of microbes was present with higher degrees of fluctuation.

Considering alpha diversity, monthly changes later exposed an annual rhythmic pattern (Figure 5B). Overall, the highest number of different phylotypes was found during spring regardless of site. There was also the smallest variation in diversity at that time, suggesting all locations to have similarly complex microbial communities early in the year after ice break-up. By summer, bacterial heterogeneity decreased across the lake where littoral regions established the largest range in species present. While the number of individual taxa then increased at shorelines during the fall, the communities offshore steadily became more homogenous from spring to winter. Sediment bacteria had similar trends to those inhabiting the upper liquid layers at littoral zones (Figure S4), which could be explained by the proximity of sampling and the frequent mixing of sand and water. However, sediment communities were distinct due to the decrease in average diversity from spring to fall that increased in variation as winter drew near, suggesting a range in community structure formed in sediments as the year progressed (Figure 5B).

**Figure 6:**
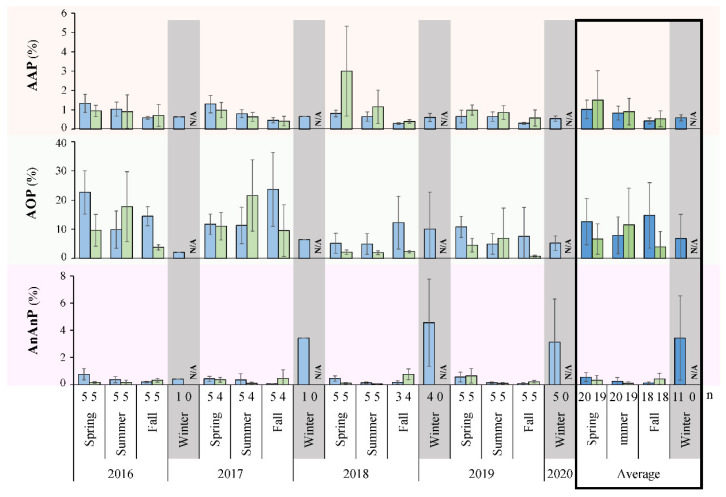
Relative proportions of phototrophs in sequenced communities. All 16S V4 rRNA genes of species and genera representing AAP, AOP, or AnAnP at limnetic (blue) and littoral (green) sites summed for each season. N/A, not analyzed; n, number of samples per season.

Based on 16S V4 rRNA gene sequences, the populations of AAP, AOP, and AnAnP were individually grouped to identify and assess their changing proportion in the bacterial community (Figure 6), using an updated list of known AAP or AnAnP (Tables S5 and S6). Seasonal patterns were found for each group, where AAP averaged 1.02 and 1.51% of the limnetic and littoral sequenced communities in the spring, respectively, dropping to 0.83 and 0.90% during the summer, and further to 0.41 and 0.54% by fall (Figure 6). AOP that included algae and cyanobacteria, made up approximately 10% of the sequences from limnetic sites at any given time, dipping during summer and winter, while inversely increasing at littoral zones midsummer (Figure 6). AOP numbers fluctuated substantially between sites and seasons, as depicted by large variations in standard deviation, whereas AAP were present and distributed more stably. The spring of 2018 was an exception, when records for Blastomonas related AAP were exceptionally high. AnAnP were quite nominal in comparison to other phototrophs during open water months, and only saw an increase in winter, dissimilar to the other populations (Figure 6). 

While all specific clades and their relative proportions were provided in Supplemental Material, the three most prevalent AAP-associated genera in sequenced communities were Gemmatimonas, Roseomonas, then Blastomonas. For AOP, Cryptomonadaceae, Aphanizomenon, and Aulacoseira were predominant, whereas the highest AnAnP were Rhodoferax, Roseiflexus, and Rhodoplanes. Among known AAP (Table S5), Rhodobacteraceae, Comamonadaceae, and Acidobacteriaceae were not detected. AnAnP were exceedingly rare, with no examples of Heliobacteria, Chlorobiaceae, Ectothiorhodospiraceae, Chromatiaceae, or Halorhodospiraceae found.

## 4. Discussion

### 4.1. Habitat Features

Lake Winnipeg has been regarded as atypical due to its shape and dimensions as the 11th largest freshwater reservoir in surface area, yet 25th by volume. Shallow in comparison to other great lakes, it has maintained a well-mixed state, where oxygen often penetrates to the bottom. As a result, thermocline-based stratification has been infrequent in the South Basin [48]. Indeed, during our study oxygen was well dispersed during open-water seasons (Table S2). As Lake Winnipeg is north of the 49th parallel, the duration of daylight was dependent on the time of year (Figure 2). Water temperature lagged behind the spring peak of available sunlight, instead reaching height in the summer, likely due to the thermostability of the vast volume of water as a slow conductor of heat. The most significant pH change was observed at limnetic zones, where a drop to 7.5 occurred in winter. That decrease, and the slight additional alkalinity of pH 8.3–8.7 found in littoral areas over offshore values around 8.2 may have been due to respective water temperatures. Shallow waters had less exchange, and the warmer conditions likely increased solubility of carbonates and/or promoted activity of microbes, such as photosynthesis and biological carbon fixation, decreasing pH. Regardless, the yearly changes of these parameters (Figure 2), matched previously reported years [9,10]. Furthermore, most metal concentrations were typically below 1 ppm and detection limits, confirming no significant toxic influence on life (Table S2). Chl *a* and *b* curves indicating AOP followed that of temperature rather than daylight (Figure A1), and the averaged Chl *a* peak of 16.5 µg/L was similar to summer maxima observed via satellite imagery from 2002–2011 [49]. While BChl *a* from anoxygenic phototrophs was nearly undetectable and often below 1 µg/L, a common occurrence in oxic lakes [50], its low levels were still recognizable and matched daylight more so than temperature or the other chlorophylls.

### 4.2. Primary Productivity in Lake Winnipeg

The seasonal in situ carbon fixation analysis of whole community productivity found littoral microbes fixed at higher rates than those taken from limnetic zones (Figure 3). These increased values were likely due to the warmer nearshore waters during the spring and summer, as temperature has been shown to affect oxygenic phototrophs [51,52], and thus may affect all carbon fixation similarly. Moreover, the maximal littoral rates decreased each fall, correlating to light availability, confirming its additional impact to fixation levels. Independently, AOP fixation was predominant during summer months, tracing the measured Chl *a* and *b* concentrations in addition to general temperature trends. In comparison, AnAnP associated productivity were marginal regardless of season, likely inhibited by the oxic nature of the habitat. Furthermore, AnAnP related rates did not correlate with BChl *a* data, suggesting this pigment was unlinked to primary productivity.

The total fixation rates at shorelines reached a spring average high of 2.0 ± 0.1 g C/m^2^/day (Figure 3), matching recently reported values for Lake Winnipeg calculated via oxygen production rather than using carbon isotopes [53]. When compared to satellite imagery-based findings from the other 11 largest freshwater lakes, Lake Winnipeg rates determined here were greater than all, yet were most equivalent to Lake Erie’s estimated mean fixation of 1.2 g C/m^2^/day [52]. Due to the well-known eutrophication of Lake Winnipeg, and confirmed Chl *a* levels that matched recent measurements, it may indeed currently have the highest rates among all great lakes. However, the relatively elevated numbers in Lake Winnipeg may simply be a result of underestimated values calculated in the recent satellite survey of other lakes, which was based solely on Chl *a* estimate without considering other types of carbon fixation. Indeed, a better match was found to earlier isotope-measured records of the Laurentian Great Lakes [54]. Specifically, Lake Ontario monthly dynamics aligned well with our three time points taken yearly, where littoral regions had maximal fixation rates in the spring, which occurred prior to the swell apparent at limnetic zones. Inversely, limnetic areas were slower to warm and cool, likely the cause of delayed fixation rate peaks. However, since Lake Ontario has also suffered from nutrient loading in its recent past, and primary production may have fluctuated, future work should include a combination of satellite imagery on Lake Winnipeg in addition to chemical isotope analyses to better support our conclusions and comparisons to other great lakes.

### 4.3. Trends in Cultivated Microbial Numbers

The height of heterotrophic cellular counts matched the peak of temperature during summer at all nearshore sites (Figure 4A). Littoral sands had 4.23 × 10^6^ and 9.05 × 10^5^ CFU/g, while its waters maintained 7.69 × 10^4^ and 1.84 × 10^4^ CFU/mL on oligotrophic and copiotrophic complex media, respectively, which matched previously reported values in freshwaters and sediments [55]. In comparison, limnetic numbers did not coordinate with temperature. This incongruence may have been due to the minimal organic carbon available offshore, where low organic production rates were observed (Figure 3). Since limnetic regions were slow to warm, autotrophic production of excess organic carbon was delayed, which in turn pushed back the bloom of heterotrophs till the fall. When considering concentrations of supplied organics, higher cell counts were found on oligotrophic OM rather than rich RO from all locations and times (Figure 4A), indicating that the natural lake bacteria were adapted to lower nutrient availability. In addition, cultivated numbers on PM were 10–100× lower than RO suggesting even fewer organisms preferred the highest level of organics provided (Table S4). R2A enumerated values fell between those counted on OM and RO, which also coincided with its organic content in between both custom-made media. Taken together, OM clearly provided the most ideal growth conditions by incurring the highest numbers of heterotrophs, that in-turn best represented the microbial community metabolism. Of note, carbon was observed at ~0.025 g/L in lake waters (Table S2), and OM had 0.1 g/L, about five times higher. This would suggest, that while common laboratory practices promote bacteria growth by supplying high levels of nutrients, more dilute concentrations closer to environmental conditions may be ideal for the cultivation of highest numbers of heterotrophs. While the amount of carbon in Lake Winnipeg were considered eutrophic, these findings also suggested that the current microbial community is accustomed to oligotrophy, and do not have preference to even higher levels of nutrients. Future work should include more dilute media for enumerations, as such composition may outperform the conditions provided in standard RO or previously considered oligotrophic OM. Follow-up microscopy may also shed light on the microbial content since cultivated counts fell short of microscopic observations from other Canadian lakes by 10–100× [22]. Later investigation could examine alive/dead cells, or if the additional microbial content had specific growth requirements not provided in laboratory.

Regarding phototrophs, few AnAnP and other anaerobes were detected (Table S4), presumably a result of the high inhibitory oxygen content. AOP were cultivated up to 9.37 × 10^3^ CFU/mL on average, peaking in summer littoral sediments and traced both the Chl *a* and *b* levels as well as the AOP associated fixation rates. The curiosity about metals in the lake, particularly metalloids, led to the study of tellurite tolerance by the aerobic heterotrophic community. Since the toxic metalloid oxide tellurite was not present in significant amounts (Table S2), it was surprising to find ~5% of all heterotrophs at any given site or season capable of resisting at least 100 µg/mL, a substantially high level (Table S4). While it had been previously postulated that 1 µg/mL of tellurite was toxic to most tested bacteria [56], our findings suggested resistance to higher levels may be more common than previously considered. Furthermore, since 47.5% of heterotrophs cultivated on RO_T_ were found to be AAP, this suggested that the medium was selective for some phototrophs, and that they may have metal transformation capabilities as part of their niche.

Compared to other photosynthesizing populations, cultured AAP counts neither followed trends in temperature dynamics and total heterotrophs, nor did they match measurements of AOP. Instead, they reached a pinnacle during the spring as 19.1 and 4.7% of total copiotrophs (Figure 4B), or 3.9 and 4.9% of oligotrophs (Figure 4C), at littoral and limnetic locations, respectively. The proportion decreased till winter each year and directly coincided with the hours of available sunlight, BChl *a* concentration, and shoreline rates of total carbon fixation. Such correlations were reasonable to find for AAP as these bacteria presumably required sunlight, and their inability to fix CO_2_ or act autotrophically meant they had to rely on other community members to provide organics [17]. In addition, AAP numbers did not match up with AnAnP associated fixation rates, dark (chemolithotrohic) reactions, or AOP fixation rates independently. Rather, AAP numbers were highest at shorelines, and particularly coincided to the maximal rates of total fixed carbon productivity (Figure 3). AAP may thus be coordinated with organisms capable of fixing inorganic carbon, by consuming excess nutrients released by autotrophic neighbors in euphotic zones. Moreover, while more AAP were culturable on organic limiting OM, it was evident that they made up a larger proportion of bacteria capable of growth on the richer RO or PM (Figure 4 and Figure S1). Obviously, they had a tolerance/ability to grow with excess nutrients, whereas several other heterotrophs did not. Hence, aerobic phototrophs were influenced by the environmental factor sunlight, and had advantage when organics were plentiful.

### 4.4. Sequenced Community Analysis

*General Community Composition:* Culture-independent analysis of 16S rRNA genes extracted and sequenced from each location indicated a rather stable bacterial community existed across the lake (Figure 5), which was reasonable as the habitat has been known to be well-mixed, therefore similarities between samples were expected [48]. While Lake Winnipeg contained a bacterial composition comparable to the average freshwater basin where *Actinobacteria* were dominant and *β-Proteobacteria* made up a large proportion [13], it was very similar to a particular few great lakes of North America. Lake Erie has been found analogous both in equivalent physical surface area and volume. In addition, it contained a similar distribution of bacterial phyla at limnetic sites during the summer [2]. However, there were vastly different cyanobacteria described. While only 0.2–0.8% of Lake Erie’s microbial community belong to AOP, and predominantly contained *Planktothrix*, Lake Winnipeg mostly had *Cryptomonadaceae*, and 7.9–11.6% of its spring to summer community were AOP (Figure 6). This divergence may have been due to the irregular presence of AOP in the ecosystem, as blooms were well known to unevenly disperse yearly and spatially because of several factors including wind currents [11]. In Lake Winnipeg, a large variance in AOP indicators was found. Chl *a* and *b* concentrations (Figure A1), AOP cultivated numbers (Table S4), and proportions of sequenced communities (Figure 6) each had dispersed standard deviation, which could be explained by their tendency to move in clumps and not be homogenous in every liter of lake water. With such considerations, the Lake Erie analysis may have missed these populations by only sampling once at limited locations. Indeed, a more recent comprehensive study quantified cyanobacterial blooms in Lake Erie using specific 16S rRNA primers for AOP when sampled in the fall [57]. These findings were both coherent with ours and more comparable to other lakes [58], suggesting future work dedicated to AOP in Lake Winnipeg may be required to assess pelagic apart from aggregate forming populations.

*Community Fluctuations:* Repeating cyclical patterns were evident for Lake Winnipeg’s bacterial populations (Figure 5C). Winter and spring samples individually had distinct groups, whereas summer and fall overlapped considerably. The warmer months likely developed homogenous compositions, as similar seasonal dynamics reaching a climax community has been observed in other locations with relatively comparable freeze-thaw cycles [58]. Lake Winnipeg’s distinct winter structure was likely influenced by the drastic habitat transformation, that had low temperatures and an encapsulating frozen surface for several months (Figure 1B). While seasons returned to similar repeated conditions present in previous years (Figure 5C), a total diversity migration was observed from 2016 to 2020 (Figure 5D). Results for both 2016 and 2017 were similar, whereas 2018–2020 clustered apart. A noticeable transition occurred in 2017, as its rarified diversity was between the 2016 maximum and 2018–2020 minimum (Figure S2B). Since most monitored environmental factors remained consistent across all 5 years (Figure 2), it was doubtful they caused the drift in bacterial composition. Instead, such a shift may have been due to the recent infestation by zebra mussel bivalves, *Dreissena polymorpha*. These invasive species were first spotted in 2013, but then undoubtedly confirmed in 2015. Perhaps their filter-feeding activity and rapid consumption of bacteria caused the slow change of microbial community composition [15,23,55], and decreased microbial diversity via selective grazing. Further monitoring of bacterial communities is therefore encouraged to see if this shift continues, or if natural decade-long fluctuations have been prone to occur in the habitat.

*Coordination of Phototroph and Environmental Dynamics:* Littoral AOP were observed maximally in the summer making up a significant proportion, 11.5% of the sequenced community (Figure 6). This followed the detected Chl *a* and *b*, highest AOP associated carbon fixation rates, as well as trends in water temperatures. They did not match the length of available daylight in spring, instead being delayed till summer. As suggested previously, temperature dependent growth and autotrophy contributed to this delay, rather than following available sunlight patterns [51]. As limnetic zones were even slower to warm than nearshore (Figure 2), lower proportions of AOP and associated fixation rates midsummer were expected and observed (Figure 3 and Figure 6). As for AnAnP, they made up a minimal part of any sequenced community during open water seasons (<0.5%), but peaked in winter at limnetic sites (Figure 6). This may have been a result of the limited number of total active organisms throughout colder months, as indicated by the few cultivated heterotrophs and other bacteria (Figure 4A; Table S4). If all heterotrophs and AOP had a winter decrease, perhaps the persisting AnAnP represented a higher proportion. As oxygen was present in the lake, even under frozen ice conditions (Table S2), these results stimulate future research to understand why certain AnAnP clades made up larger winter values.

The sequenced AAP of the community maintained a seasonal pattern similar to that of the culture-based enumerations, maintaining the highest proportion in the spring, decreasing throughout the year (Figure 4B,C and Figure 6). Up to 19.1% of the cultured heterotrophs on RO were AAP, whereas only 1.5–3.0% of the total sequences were from these phototrophs in spring shoreline waters. The diminished values estimated by sequencing suggested not all species present could grow on the provided RO or OM media. However, the corresponding trends between both techniques strongly inferred that cultivation reflected a fair vision of the microbial community, and that a majority of the bacteria were likely aerobic heterotrophs. In addition, the combination of applied methods permitted several comparisons to other lake studies. The highest cultured AAP count was 2.39 × 10^4^ CFU/mL (Figure S1), a number similar to mid-summer microscopic observations of other Canadian lakes [22]. Seasonal fluctuations of AAP have been analyzed infrequently in large freshwater habitats, with notable studies from the small Ĉertovo and Vlkov Lakes, Czechia [50,59], and alpine Gossenköllesee Lake, Austria [60]. In each case, a culture-independent autofluorescence technique was used to detect anoxygenic phototrophs, reporting AAP to be prevalent in the warmest months. The latter two studies also correlated their high numbers to dissolved organic carbon trends. Dependency on available nutrients was further confirmed when AAP where additionally shown to follow diel changes of sunlight and carbon fixation in a natural setting [61]. Such associations were notable, as they supported our results produced from Lake Winnipeg. Here, AAP peaked during the spring, persisting throughout the open water seasons. Their numbers were directly associated with the amount of daylight available (Figure 2), which also aligned to when the most carbon was fixed at shorelines (Figure 3). These comparable trends strongly suggested that AAP followed seasonal fluctuations reflecting on each part of their namesake. When there is ample oxygen, light, and organic carbon, they thrive. A recent pertinent study of several freshwater lakes in Quebec, Canada reported that while AAP numbers followed light availability, they negatively correlated to water clarity [22]. This was likely due to their requirement of organic carbon, as translucent water has been indicative of an extremely oligotrophic environment, with few microbes and highly limited available nutrients.

In the area of Lake Winnipeg, the longest daylight occurred during spring, which was the same period for the highest rate of carbon fixation. This timing was therefore optimal for light-dependent phototrophs, as well as heterotrophs consuming nutrients produced by close neighbors. AAP were uniquely persistent during the early open-water season, as the conditions provided a selective advantage for them to thrive. Of note, some growth experiments have reported AAP to promote BChl *a* production only after incubation at refrigerant temperatures (4–8 °C) [62], which may suggest adaptation to the colder spring, when sunlight was present. The low presence of AAP estimated in sequenced winter communities and cultivated on plates both revealed cold temperatures played a minor inhibitory role. The analysis of additional habitats far from the equator that maintain separate daylight and temperature shifts may help to confirm which parameters are most influential to AAP growth and photosynthesis. Furthermore, the analysis of isolates from such environments and their thermal tolerances in addition to pigment synthesis may elude how influential these factors are on growth.

*AAP Diversity in Lake Winnipeg:* Of the known taxa that contain AAP (Table S5), Lake Winnipeg samples had representative DNA from *Gemmatimonadota*: *Gemmmatimonas* and proteobacterial *Acetobacteraceae*: *Roseomonas*; *Methylobacteriaceae*: *Methylobacterium*, *Methylorubrum*; *Rhodospirillaceae*; *Sphingomonadaceae*: *Polymorphobacter*, *Blastomonas*; *Erythrobacteraceae*: *Porphyrobacter*; *Rhodocyclaceae*; and *Halieaceae*. Interestingly, no sequences were detected from AAP of the β-proteobacterial *Comamonadaceae* group, which included *Limnohabitans* and *Roseateles*. While *Limnohabitans* has been indicated in several lakes [63], perhaps its preference for oligotrophy, and the eutrophic nature of Lake Winnipeg let other organisms to out compete them. Furthermore, no AAP from *Rhodobacteraceae* were found in the sequenced communities. Likely, along with *Roseateles* [64], this was because most known examples such as *Roseobacter* [65], *Roseicyclus* [66], and *Charonomicrobium* [67] hailed from marine and salt water habitats. In addition, no representatives of the newly discovered saline-preferring prosthecate AAP *Photocaulis* of *Maricaulales* were detected [68]. Since Lake Winnipeg is freshwater, the presence of marine AAP was not expected. Finally, the unusual *Acidobacteriota*: *Chloracidobacterium* were also not observed. This was predicted since the representative type species was isolated from a hot spring, being thermophilic and microaerophilic [69], whereas Lake Winnipeg had quite moderate temperatures, and was well-aerated (Figure 2, Table S2). Future analysis of the Lake Winnipeg DNA dataset may reveal additional AAP species when reference databases are updated. Later work on isolated and purified strains from this habitat could also identify each taxon observed in the sequenced community, shedding light on their individuality and physiology of the diverse species present.

In summary, the well-mixed nature of Lake Winnipeg has maintained stable microbial communities that fluctuate seasonally with a common cyclical pattern, returning to an established composition each year. Littoral shorelines and limnetic open water had distinctly separate population dynamics, containing related yet specific diversity and culturable numbers of heterotrophs. While AOP were variably present, AAP made up a stable important component of the microbial community highest in the spring, decreasing throughout the year. Their levels clearly coincided with the available daylight in all areas, BChl *a* levels, and, particularly, with total carbon fixation rates at littoral zones, confirming that they were promoted when light and organic carbon is available. In comparison, temperature, pH, and physico-chemistry including metal concentrations may only have minor impacts on their population dynamics. Future research will corroborate if similar factors play important roles for AAP ecology in other habitats.

## Data Availability

The eDNA sequenced from each site via MiSeq (Illumina) was deposited on the National Center for Biotechnology Information Sequence Read Archive as BioProject PRJNA603112.

## Supplementary Materials

The following supporting information can be downloaded at: https://www.mdpi.com/article/10.3390/microorganisms10091690/s1, Figure S1: AAP counts on various complex media; Figure S2: Rarefaction curves of sequenced microbial communities; Figure S3: Alternative colorations of PCoA ordination based on Jaccard diversity distance matrix; Figure S4: Littoral community composition of sediment compared to liquid samples; Table S1: Sampling dates and environmental parameters; Table S2: Average elemental composition of limnetic site samples; Table S3: Media composition used to cultivate different groups of microorganisms; Table S4: Enumeration data from all individual sites and seasons; Table S5: AAP type species used as reference for eDNA 16S V4 rRNA gene analysis; Table S6: AnAnP type species used as reference for eDNA 16S V4 rRNA gene analysis. References [70,71,72,73,74,75,76,77,78,79,80,81,82,83,84,85,86,87,88,89,90,91,92] are cited in the Supplementary Materials.
