# Supplementary material for: Seasonal Dynamics of Lake Winnipeg’s Microbial Communities Reveal Aerobic Anoxygenic Phototrophic Populations Coincide with Sunlight Availability
Source: Microorganisms. 2022 Aug 23;10(9):1690. doi: 10.3390/microorganisms10091690 (PMC9501198; doi:10.3390/microorganisms10091690)
Supplement: Supplementary file 1 [file microorganisms-10-01690-s001.zip › Supplimental_Figures.pdf]

Seasonal dynamics of Lake Winnipeg's microbial communities reveal aerobic anoxygenic phototrophic populations to coincide with sunlight availability

*Steven B. Kuzyk, Xiao Ma, and Vladimir Yurkov**

Microbiology Department, University of Manitoba, Winnipeg, MB, R3T 2N2, Canada
**Corresponding author*

Supplemental Figures:

Figure S1. AAP counts on various complex media.

Figure S2. Rarefaction curves of sequenced microbial communities.

Figure S3. Alternative colourations of PCoA ordination based on Jaccard diversity distance matrix.

Figure S4. Littoral community composition of sediment compared to liquid samples.

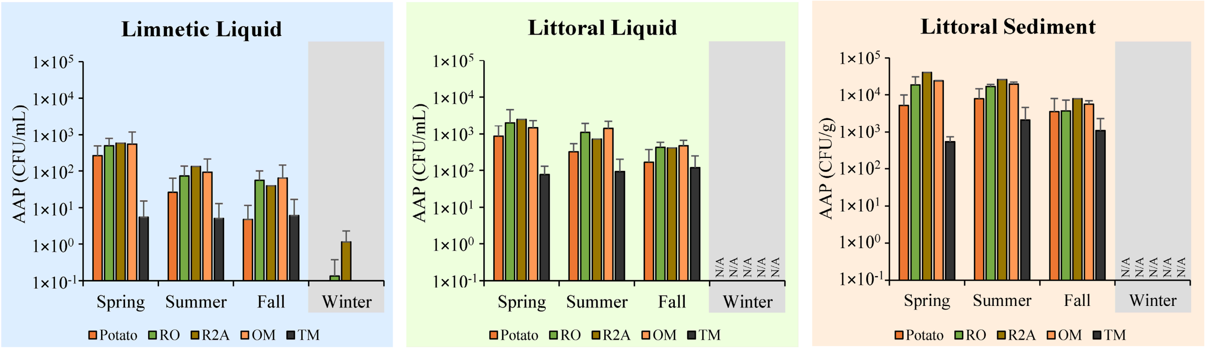

Figure S1. AAP counts on various complex media. Enumerated values from all limnetic water and littoral water/sediment samples, averaged by season. N/A, not analyzed.

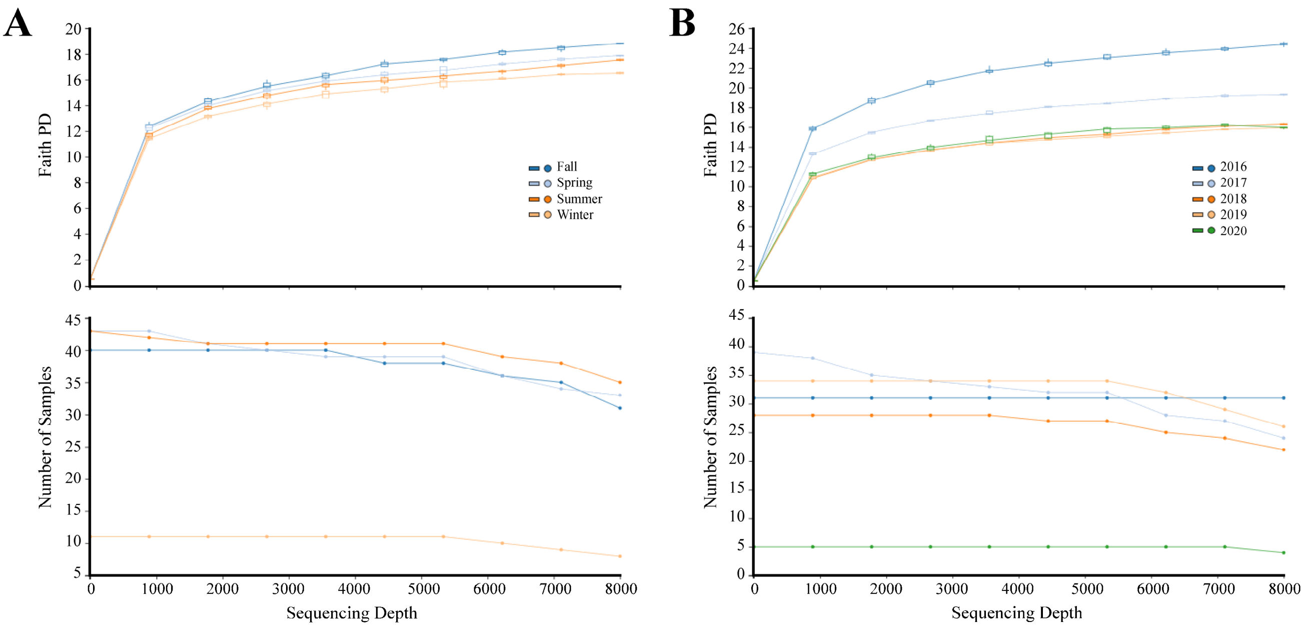

Figure S2. Rarefaction curves of sequenced microbial communities. (A) Yearly and **(B)** seasonal totals of 16S V4 rRNA genes reached a plateau in diversity, when all samples reached sufficient sequencing depth.

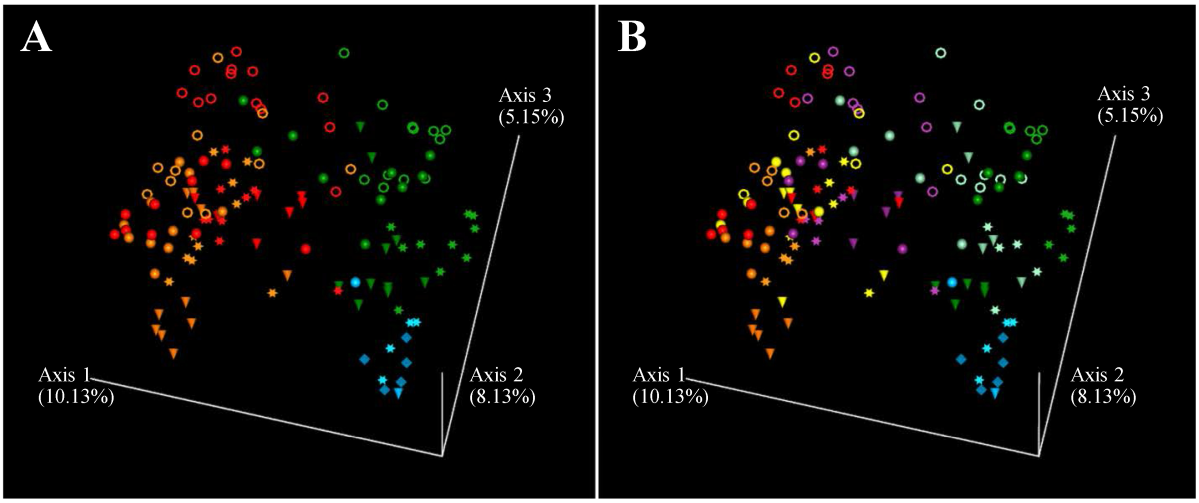

Figure S3. Alternative colouration of PCoA ordination based on Jaccard diversity distance matrix. **(A)** Seasons included: Winter, blue; Spring, green; Summer, orange; Fall, red. **(B)** Eupotic zones seperated: Winter limnetic, blue; Spring limnetic, green; Spring littoral, teal; Summer limnetic, orange; Summer littoral, yellow; Fall limnetic, red; Fall littoral, purple.

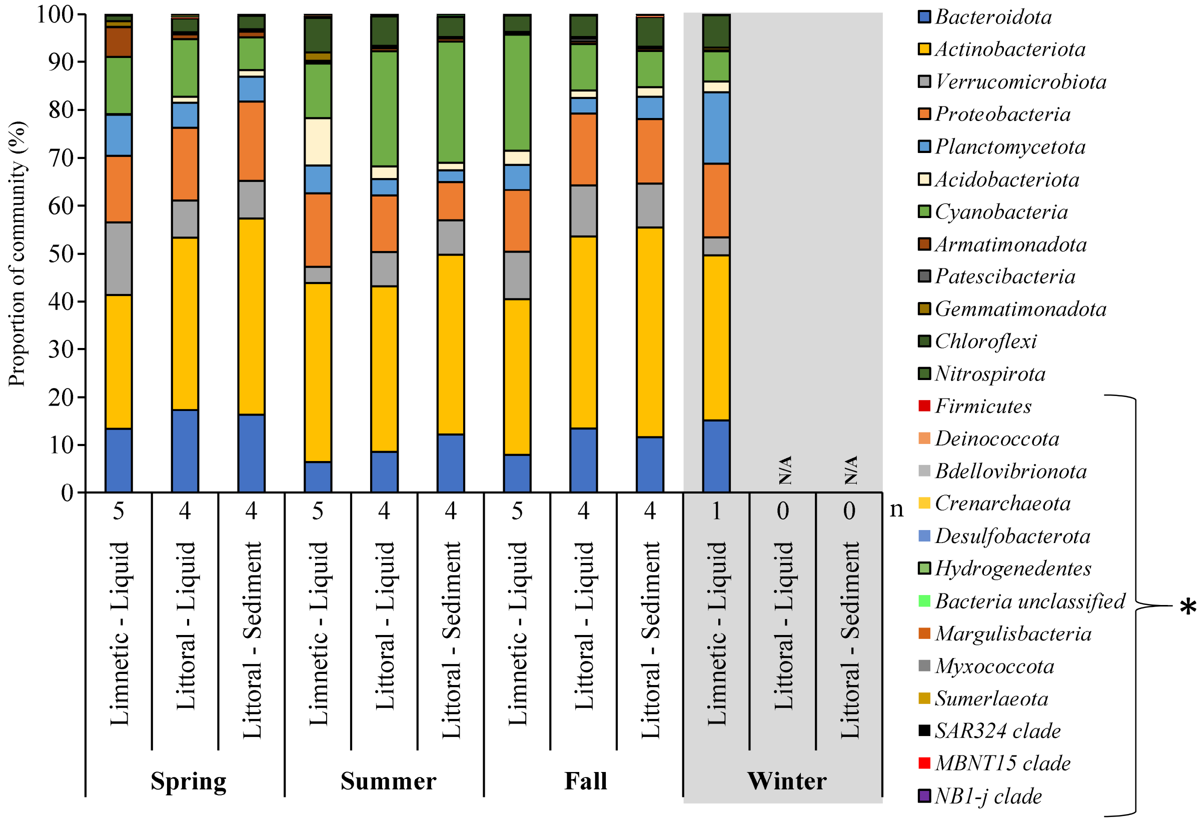

Figure S4. Littoral community composition of sediment compared to liquid samples. Zone averages from Spring 2017 to Winter of 2018. N/A, not analyzed; n, number of samples per season. *, *Minimal Clades (<1%).*
