# Supplementary material for: Seasonal Dynamics of Lake Winnipeg’s Microbial Communities Reveal Aerobic Anoxygenic Phototrophic Populations Coincide with Sunlight Availability
Source: Microorganisms. 2022 Aug 23;10(9):1690. doi: 10.3390/microorganisms10091690 (PMC9501198; doi:10.3390/microorganisms10091690)
Supplement: Supplementary file 1 [file microorganisms-10-01690-s001.zip › Supplimental_Tables.pdf]

Seasonal dynamics of Lake Winnipeg's microbial communities reveal aerobic anoxygenic phototrophic populations to coincide with sunlight availability

*Steven B. Kuzyk, Xiao Ma, and Vladimir Yurkov**

Microbiology Department, University of Manitoba, Winnipeg, MB, R3T 2N2, Canada
**Corresponding author*

<u>Supplemental Tables:</u>

Table S1. Sampling dates and environmental parameters.

Table S2. Average elemental composition of limnetic site samples.

Table S3. Media composition used to cultivate different groups of microorganisms.

Table S4. Enumeration data from all individual sites and seasons.

Table S5. AAP type species used as reference for eDNA 16S V4 rRNA gene analysis.

Table S6. AnAnP type species used as reference for eDNA 16S V4 rRNA gene analysis.

Table S1. Sampling dates and environmental parameters. Listed for each site (**1-10**).

Year	Season	Characteristic	1 Littoral	2 Littoral	3 Littoral	4 Littoral	5 Littoral	6 Limnetic	7 Limnetic	8 Limnetic	9 Limnetic	10 Limnetic
2016	**Spring**	Date	2016.06.14	2016.06.14	2016.06.14	2016.06.14	2016.06.14	2016.06.02	2016.06.02	2016.06.07	2016.06.07	2016.06.07
		Time	8:12	14:07	10:24	11:27	12:16	12:20	15:10	11:52	14:15	17:45
		Daylight (h)	16:26:57	16:24:45	16:26:16	16:27:42	16:26:48	16:15:22	16:16:51	16:24:32	16:18:15	16:25:47
		Water pH	8.3	8.6	8.3	8.6	8.5	8.1	8.5	8.1	8.2	7.8
		Water (°C)	14.0	13.0	15.0	11.0	18.0	12.0	13.0	14.2	15.1	14.6
		Air (°C)	26.0	28.0	27.0	27.0	24.0	10.3	12.0	10.5	15.0	12.6
		Water clarity (m)	0.81	0.50	0.20	0.30	0.76	0.80	1.10	1.25	1.05	0.8
		Light intensity (lux)	106000	125500	126300	124700	127400	78000	107800	126700	127000	33600
	**Summer**	Date	2016.08.25	2016.08.25	2016.08.25	2016.08.25	2016.08.25	2016.07.29	2016.07.29	2016.07.19	2016.07.19	2016.07.19
		Time	7:38	9:13	9:51	10:37	11:25	18:00	14:03	11:58	14:44	18:23
		Daylight (h)	13:54:19	13:53:29	13:54:03	13:54:36	13:54:16	15:20:21	15:27:29	15:54:59	15:56:00	15:56:04
		Water pH	8.5	8.4	8.6	8.8	8.8	8.4	8.3	8.0	7.8	8.1
		Water (°C)	23.0	25.0	23.0	22.0	22.0	22.7	22.1	20.9	21.1	21.1
		Air (°C)	13.0	17.0	18.5	18.0	19.0	28.5	24.0	23.2	23.5	23.5
		Water clarity (m)	0.20	0.10	0.20	0.20	0.10	0.50	0.75	0.40	0.30	0.4
		Light intensity (lux)	47300	106000	40500	46600	129100	120400	100400	50500	115300	102700
	**Fall**	Date	2016.10.17	2016.10.17	2016.10.17	2016.10.17	2016.10.17	2016.09.26	2016.09.26	2016.09.12	2016.09.12	2016.09.12
		Time	9:57	11:25	12:12	12:50	13:25	10:31	12:51	13:00	19:11	14:33
		Daylight (h)	10:35:32	10:36:13	10:35:45	10:35:19	10:35:35	11:46:46	11:58:12	12:48:05	12:48:16	12:48:17
		Water pH	8.0	8.3	8.3	8.3	8.5	8.0	8.2	8.3	8.3	8.2
		Water (°C)	9.0	8.0	9.0	13.0	14.0	14.9	16.1	18.9	14.6	18.9
		Air (°C)	13.0	11.0	12.5	13.0	15.0	10.7	12.5	13.5	12.0	15.5
		Water clarity (m)	0.10	0.20	0.20	0.20	0.13	0.35	0.40	0.25	0.45	0.5
		Light intensity (lux)	116800	117500	126600	127800	122800	22000	45000	30000	3050	32000
2017	**Winter**	Date	N/A	N/A	N/A	N/A	N/A	N/A	N/A	2017.02.22	N/A	N/A
		Time	N/A	N/A	N/A	N/A	N/A	N/A	N/A	12:03	N/A	N/A
		Daylight (h)	N/A	N/A	N/A	N/A	N/A	N/A	N/A	10:31:04	N/A	N/A
		Water pH	N/A	N/A	N/A	N/A	N/A	N/A	N/A	7.8	N/A	N/A
		Water (°C)	N/A	N/A	N/A	N/A	N/A	N/A	N/A	0.1	N/A	N/A
		Air (°C)	N/A	N/A	N/A	N/A	N/A	N/A	N/A	-1.4	N/A	N/A
		Water clarity (m)	N/A	N/A	N/A	N/A	N/A	N/A	N/A	1.80	N/A	N/A
		Light intensity (lux)	N/A	N/A	N/A	N/A	N/A	N/A	N/A	N/A	N/A	N/A
	**Spring**	Date	2017.05.08	2017.05.08	2017.05.08	2017.05.08	2017.05.08	2017.05.31	2017.05.31	2017.06.06	2017.06.06	2017.06.06
		Time	7:45	9:15	9:55	10:38	11:10	11:54	14:03	15:50	21:40	17:50
		Daylight (h)	15:10:10	15:08:42	15:09:43	15:10:40	15:10:04	16:11:24	16:12:52	16:22:54	16:24:03	16:24:09
		Water pH	8.7	8.4	8.4	8.2	8.7	8.4	8.4	8.5	8.4	8.3
		Water (°C)	16.0	19.5	17.5	17.0	17.5	11.7	12.7	15.1	13.2	16.2
		Air (°C)	22.0	18.0	20.5	24.5	22.0	18.0	13.6	20.7	20.6	19.6
		Water clarity (m)	0.03	0.38	0.13	0.13	0.05	1.40	1.40	1.50	1.40	1.5
		Light intensity (lux)	106100	114300	122800	119300	126100	127000	129000	125900	60000	123200
	**Summer**	Date	2017.08.04	2017.08.17	2017.08.17	2017.08.17	2017.08.17	2017.08.04	2017.08.04	2017.07.25	2017.07.25	2017.07.25
		Time	21:00	8:48	9:45	10:25	11:06	10:46	12:45	12:06	16:27	14:29
		Daylight (h)	15:07:10	14:22:39	14:23:23	14:24:05	14:23:39	15:08:38	15:09:40	16:19:44	15:40:51	15:40:55
		Water pH	8.3	9.1	8.8	8.9	9.2	8.2	8.3	8.2	8.3	8.1
		Water (°C)	22.5	22.0	24.0	22.0	22.5	22.0	22.9	20.8	22.6	21.3
		Air (°C)	22.2	14.0	18.0	18.5	19.0	20.3	22.1	21.8	21.8	22.6
		Water clarity (m)	0.90	1.30	1.17	1.04	>1.32	0.45	0.70	0.35	0.25	0.4
		Light intensity (lux)	N/A	74400	109700	110100	120400	128700	143100	129500	11340	56200
	**Fall**	Date	2017.10.01	2017.10.01	2017.10.15	2017.10.15	2017.10.15	2017.10.04	2017.10.04	2017.09.18	2017.09.18	2017.09.18
		Time	8:23	10:21	7:30	8:10	8:50	13:35	13:47	18:20	23:10	21:20
		Daylight (h)	11:36:25	11:36:38	11:36:29	11:36:21	11:36:26	11:24:47	11:24:34	12:26:01	12:26:06	12:26:07
		Water pH	8.8	9.1	8.0	8.2	8.1	8.2	8.2	8.5	8.0	8.4
		Water (°C)	9.5	13.0	16.0	15.0	15.0	13.1	12.3	15.5	13.0	15.4
		Air (°C)	14.0	17.0	4.0	5.0	5.5	13.4	11.3	11.9	15.5	14.8
		Water clarity (m)	0.84	0.48	0.90	1.00	0.35	0.90	0.90	0.79	1.07	0.79
		Light intensity (lux)	43700	61000	2020	16240	25220	21700	41800	125400	72900	119300
2018	**Winter**	Date	N/A	N/A	N/A	N/A	N/A	2018.02.26	N/A	2018.02.26	N/A	N/A
		Time	N/A	N/A	N/A	N/A	N/A	15:30	N/A	10:35	N/A	N/A
		Daylight (h)	N/A	N/A	N/A	N/A	N/A	10:45:52	N/A	10:45:22	N/A	N/A
		Water pH	N/A	N/A	N/A	N/A	N/A	7.71	N/A	7.72	N/A	N/A
		Water (°C)	N/A	N/A	N/A	N/A	N/A	-0.1	N/A	-0.1	N/A	N/A
		Air (°C)	N/A	N/A	N/A	N/A	N/A	-5.0	N/A	-2.0	N/A	N/A
		Water clarity (m)	N/A	N/A	N/A	N/A	N/A	1.00	N/A	1.40	N/A	N/A
		Light intensity (lux)	N/A	N/A	N/A	N/A	N/A	N/A	N/A	N/A	N/A	N/A

Table S1. (continued)

Year	**Season**	Characteristic	**1** Littoral	**2** Littoral	**3** Littoral	**4** Littoral	**5** Littoral	**6** Limnetic	**7** Limnetic	**8** Limnetic	**9** Limnetic	**10** Limnetic
2018	**Spring**	Date	2018.05.29	2018.05.29	2018.05.29	2018.05.29	2018.05.29	2018.06.13	2018.06.12	2018.06.13	2018.06.04	2018.06.04
		Time	7:42	9:19	10:07	10:52	11:35	9:26	10:00	12:53	8:59	10:14
		Daylight (h)	16:05:02	16:03:03	16:04:25	16:05:42	16:04:54	16:23:20	16:22:17	16:22:34	16:20:45	16:30:35
		Water pH	9.2	9.4	9.1	9.3	8.7	8.2	8.1	8.2	8.0	8.2
		Water (°C)	15.0	19.0	15.5	16.0	20.0	16.2	13.7	12.9	17.2	15.6
		Air (°C)	20.0	25.5	27.5	27.0	26.0	16.5	16.0	18.5	20.3	20
		Water clarity (m)	1.07	0.05	0.52	1.00	0.97	1.96	1.20	2.33	2.33	2.33
		Light intensity (lux)	60400	118800	123500	124900	124800	99000	13700	92100	119400	124200
	**Summer**	Date	2018.07.30	2018.08.08	2018.08.08	2018.08.08	2018.08.08	2018.08.07	2018.07.31	2018.08.07	2018.07.30	2018.07.30
		Time	3:50	8:07	8:51	9:36	10:17	10:35	9:44	12:00	12:08	10:39
		Daylight (h)	15:23:11	14:53:45	14:54:41	14:55:33	14:55:00	14:59:45	15:22:56	15:00:54	15:01:39	15:01:43
		Water pH	8.5	8.8	8.4	8.6	8.8	8.3	8.2	8.3	8.1	8.2
		Water (°C)	21.5	24.0	22.0	22.0	21.5	20.0	21.2	20.2	21.1	20.6
		Air (°C)	31.0	22.0	24.0	24.5	24.0	19.1	18.3	18.9	15.8	25.6
		Water clarity (m)	0.81	0.42	0.50	0.58	0.20	0.39	0.84	0.67	0.56	0.53
		Light intensity (lux)	105300	61500	82900	86900	104700	1170	2340	9850	3240	8320
	**Fall**	Date	2018.10.22	2018.10.08	2018.10.08	2018.10.08	2018.10.08	2018.10.22	-	2018.09.25	-	2018.09.25
		Time	18:00	8:34	9:20	10:15	10:50	10:19	-	11:32	-	13:53
		Daylight (h)	10:18:59	11:11:19	11:11:03	11:10:48	11:10:57	10:18:11	Ship	12:00:03	Ship	12:00:02
		Water pH	8.0	8.5	8.3	8.3	8.3	8.2	Repaired	8.4	Repaired	8.3
		Water (°C)	3.0	4.5	6.0	6.0	6.5	4.1	No	12.1	No	12.2
		Air (°C)	1.5	4.0	4.5	4.5	6.0	2.6	Sampling	7.2	Sampling	9.3
		Water clarity (m)	0.25	0.15	0.41	0.67	0.29	0.65	-	0.50	-	0.86
		Light intensity (lux)	6890	4800	11240	14090	2250	12270	-	128700	-	122800
2019	**Winter**	Alternative Sites	N/A	N/A	N/A	N/A	N/A	W10	**W1**	W9	**W6**	**W8**
		Date	N/A	N/A	N/A	N/A	N/A	2019.02.28	2019.02.20	2019.02.28	2019.02.21	2019.02.28
		Time	N/A	N/A	N/A	N/A	N/A	13:20	9:00	9:30	10:50	11:30
		Daylight (h)	N/A	N/A	N/A	N/A	N/A	10:41:11	10:12:29	10:40:37	10:19:28	10:50:10
		Water pH	N/A	N/A	N/A	N/A	N/A	7.8	8.17	7.7	8.09	7.96
		Water (°C)	N/A	N/A	N/A	N/A	N/A	-0.5	-1.0	-0.7	-1.0	0.8
		Air (°C)	N/A	N/A	N/A	N/A	N/A	-8.0	-18.9	-9.0	-17.1	-10
		Water clarity (m)	N/A	N/A	N/A	N/A	N/A	1.8	0.70	1.2	0.60	0.6
		Light intensity (lux)	N/A	N/A	N/A	N/A	N/A	N/A	N/A	N/A	N/A	N/A
	**Spring**	Sites	1	2	3	4	5	6	7	8	9	10
		Date	2019.06.03	2019.06.08	2019.06.08	2019.06.05	2019.06.08	2019.06.03	2019.06.03	2019.06.05	2019.06.05	2019.06.05
		Time	7:07	7:38	8:28	7:00	9:26	11:30	2:30	10:25	16:45	13:12
		Daylight (h)	16:13:44	16:18:44	16:20:13	16:17:33	16:20:44	16:15:44	16:17:12	16:20:50	16:21:58	16:22:05
		Water pH	8.4	7.5	8.1	8.0	7.6	8.2	8.4	8.5	8.0	8.5
		Water (°C)	13.0	16.0	13.0	12.0	15.5	10.5	11.1	11.2	12.4	12
		Air (°C)	12.9	18.0	18.0	12.1	17.0	10.2	11.3	12.9	18.7	17.1
		Water clarity (m)	0.76	0.38	0.34	0.53	>1.50	0.9	0.8	1.2	1.6	1.1
		Light intensity (lux)	12020	8550	8140	106800	28700	6630	37800	126200	48400	66200
	**Summer**	Date	2019.08.06	2019.08.13	2019.08.13	2019.08.13	2019.08.13	2019.08.06	2019.08.06	2019.07.23	2019.07.23	2019.07.23
		Time	7:20	7:02	8:20	10:20	9:05	11:33	2:15	11:24	17:15	14:05
		Daylight (h)	15:02:21	14:37:59	14:38:49	14:39:36	14:39:07	15:03:42	15:04:42	15:46:41	15:47:39	15:47:44
		Water pH	8.4	8.7	8.4	8.3	8.2	8.4	8.3	8.0	8.1	8
		Water (°C)	26.0	25.0	23.0	24.0	19.0	22.2	22.1	21.8	21.3	22.7
		Air (°C)	16.7	15.5	25.5	24.0	23.0	21.8	24.8	23.1	23.2	24.1
		Water clarity (m)	0.65	0.39	0.61	0.51	1.02	0.50	1.40	0.60	0.85	0.8
		Light intensity (lux)	15310	6030	95500	118000	116500	41500	64600	119700	122400	117800
	**Fall**	Date	2019.10.02	2019.09.22	2019.09.22	2019.09.22	2019.09.22	2019.10.02	2019.10.02	2019.09.16	2019.09.16	2019.09.16
		Time	8:55	8:11	11:03	12:28	11:55	13:10	16:10	13:03	19:00	15:45
		Daylight (h)	11:34:29	12:12:21	12:12:24	12:12:26	12:12:25	13:28:13	11:34:07	12:35:34	12:35:42	12:35:42
		Water pH	8.2	8.2	8.1	8.0	8.2	8.2	8.2	8.2	8.0	8.2
		Water (°C)	9.0	15.0	15.5	14.0	16.0	11.9	10.4	15.8	16.8	16.2
		Air (°C)	3.1	14.5	16.0	17.0	14.5	4.7	5.2	21.4	23.1	27.7
		Water clarity (m)	0.33	0.17	0.27	0.19	0.13	0.50	0.70	0.50	0.09	0.85
		Light intensity (lux)	4380	10980	122000	133500	192000	29400	18800	35200	N/A	7750
2020	**Winter**	Alternative Sites	N/A	N/A	N/A	N/A	N/A	W10	**W1**	W9	**W6**	**W8**
		Date	N/A	N/A	N/A	N/A	N/A	2020.02.24	2020.03.04	2020.02.24	2020.03.03	2020.02.24
		Time	N/A	N/A	N/A	N/A	N/A	14:10	9:30	13:15	9:30	10:30
		Daylight (h)	N/A	N/A	N/A	N/A	N/A	10:36:30	11:05:44	10:35:53	11:03:09	10:33:30
		Water pH	N/A	N/A	N/A	N/A	N/A	6.3	6.66	7.1	6.53	6.34
		Water (°C)	N/A	N/A	N/A	N/A	N/A	0.4	-9.2	0.3	-10.1	-2.9
		Air (°C)	N/A	N/A	N/A	N/A	N/A	-0.9	-0.8	-0.5	-0.7	-0.6
		Water clarity (m)	N/A	N/A	N/A	N/A	N/A	1.00	0.70	0.70	0.50	0.6
		Light intensity (lux)	N/A	N/A	N/A	N/A	N/A	N/A	N/A	N/A	N/A	N/A

N/A, Not analyzed

Table S2. Average elemental composition of limnetic site samples. Listed as ppm.

	2016 Spring	Summer	Fall	2017 Winter	Spring	Summer	Fall	2018 Winter
**Ag**	L0.00001	0.000005	L0.00001	L0.00001	L0.00001	L0.00001	L0.00001	L0.00001
**Al**	0.191	1.5035	1.42	0.213	0.113	0.68	0.261	0.12
**As**	0.00188	0.001825	0.002395	0.00116	0.001645	0.00207	0.002885	0.001135
**Ba**	0.02625	0.0314	0.0371	0.00996	0.0229	0.0327	0.03275	0.0117
**Be**	L0.0002	L0.0002	L0.0002	L0.0002	L0.0002	L0.0001	L0.0001	L0.0001
**Bi**	L0.0002	L0.0002	L0.0002	L0.0002	L0.0002	L0.00005	L0.00005	L0.00005
**B**	0.0315	0.0245	0.0305	L0.01	0.0245	0.033	0.038	0.01
**Cd**	0.00001	0.00002	0.00002	0.00001	L0.00001	0.00002	0.00000955	0.0000179
**Ca**	33.65	25.4	27.85	11.5	28.2	32	36.1	15.25
**C (organic)**	10.175	8.775	10.35	12.24	10.175	9.15	11.85	12.6
**C (inorganic)**	17.575	20.025	20.05	9.26	19.275	22	20.775	10.5
**C (total)**	27.75	28.8	30.4	21.5	29.45	31.15	32.625	23.1
**Cs**	L0.0001	0.000205	0.00018	L0.0001	L0.0001	0.000099	0.0000445	0.0000945
**Cl**	12.1	8.83	11.375	1.66	9.58	9.72	11.71	2
**Cr**	0.00034	0.002155	0.002055	0.00046	0.00023	0.001105	0.00048	0.00031
**Co**	L0.0002	0.0005	0.000505	L0.0002	L0.0002	0.00038	0.000175	L0.0001
**Cu**	0.001845	0.002465	0.002695	0.00134	0.001705	0.002355	0.00201	0.001565
**Fe**	0.1725	1.233	1.163	0.21	0.1025	0.7435	0.2525	0.1155
**K**	3.795	3.105	3.66	0.931	3.06	3.585	3.875	0.988
**Li**	0.01545	0.01125	0.0151	L0.002	0.01265	0.01585	0.0194	0.00365
**Mg**	15.3	11.655	14.32	3.66	14.05	15.65	18.2	5.17
**Mn**	0.01865	0.0561	0.0511	0.00672	0.02256	0.06615	0.0204	0.01021
**Hg**	N/A	N/A	N/A	N/A	N/A	N/A	N/A	N/A
**Mo**	0.000895	0.00069	0.00086	L0.0002	0.0007	0.00079	0.001058	0.0002075
**Ni**	0.00168	0.00275	0.00287	0.00084	0.001415	0.00256	0.00174	0.000885
**N**	0.8575	0.8425	0.8025	0.59	0.5875	0.765	0.7425	0.505
**N (Kjeldahl)**	0.6925	0.625	0.5775	0.41	0.5875	0.6125	0.6875	0.385
**NH_3**	0.01375	0.0105	0.01375	0.04	0.01025	0.0465	0.02125	L0.01
**NO_2+NO_3**	0.1655	0.218	0.22425	0.181	N/A	0.1515	0.0795	0.1215
**Na**	16.15	11.82	14.505	2.37	13.55	15.15	17.8	3.055
**O**	10.05	8.05	8.8	10.6	10.2	7.25	9.5	14.25
**P**	0.065	0.09325	0.13625	0.032	0.05575	0.11075	0.104	0.032
**P (dissolved)**	0.0395	0.0595	0.08425	0.024	0.0415	0.07475	0.07925	0.0235
**P (inorganic)**	0.054	0.0825	0.12425	0.028	0.0455	0.10575	0.098	0.03
**P (particulate)**	0.0265	0.041	0.054	L0.014	0.016	0.042	0.03	L0.014
**P (reactive)**	0.0405	0.06425	0.0995	0.026	0.03675	0.09075	0.0965	0.024
**P (acid hydrolizable)**	0.01625	0.0215	0.02675	L0.014	L0.014	0.01525	L0.014	L0.014
**Pb**	0.000115	0.000585	0.000555	0.00013	0.0001	0.000473	0.0001725	0.0001115
**Rb**	0.001765	0.004075	0.0042	0.0016	0.001555	0.00289	0.002265	0.00164
**Sb**	L0.0002	L0.0002	L0.0002	L0.0002	L0.0002	0.000195	0.00017	0.00012
**Se**	0.00025	0.00023	0.000275	L0.0001	0.000225	0.0002685	0.0003	0.0001595
**Si**	1.08	6.51	7.405	2.36	0.595	4.765	4.93	2.1
**Sr**	0.10195	0.0768	0.09675	0.0233	0.0866	0.1009	0.119	0.02915
**Sn**	L0.0002	L0.0002	L0.0002	L0.0002	L0.0002	L0.0001	L0.0001	L0.0001
**SO_4**	50.8	37.8	47.9	3.74	44.7	52	67.25	3.015
**Te**	L0.0002	L0.0002	L0.0002	L0.0002	L0.0002	L0.0002	L0.0002	L0.0002
**Tl**	L0.0001	L0.0001	L0.0001	L0.0001	L0.0001	0.000015	L0.00001	L0.00001
**Th**	L0.0001	0.00036	0.00031	L0.0001	L0.0001	0.000155	L0.0001	L0.0001
**Ti**	0.00578	0.0538	0.0487	0.00661	0.00328	0.02135	0.007475	0.0035
**U**	0.0015	0.001085	0.00127	0.00011	0.001365	0.001535	0.00177	0.0001115
**V**	0.00189	0.005085	0.005545	0.00077	0.001475	0.00346	0.002965	0.000785
**Zn**	0.0014	0.0048	0.0053	0.0038	L0.002	0.004	L0.003	0.00355
**Zr**	L0.0004	0.00167	0.00149	L0.0004	L0.0004	0.000707	0.000336	0.0001625

Table S2. Continued. Listed as ppm.

	2018			2019				2020
	Spring	Summer	Fall	Winter	Spring	Summer	Fall	Winter
**Ag**	L0.00001	L0.00001	L0.00001	L0.00001	L0.00001	L0.00001	L0.00001	L0.00001
**Al**	0.1485	0.383	0.274	0.062	0.1109	0.27125	0.55075	0.151
**As**	0.001745	0.002185	0.00301	0.001145	0.002095	0.0024725	0.00307	0.00102
**Ba**	0.02315	0.02885	0.03235	0.01185	0.0224	0.026625	0.0351	0.01265
**Be**	L0.0001	L0.0001	L0.0001	L0.0001	L0.0001	L0.0001	L0.0001	L0.0001
**Bi**	L0.00005	L0.00005	L0.00005	L0.00005	L0.00005	L0.00005	L0.00005	L0.00005
**B**	0.0275	0.026	0.035	L0.01	0.0195	0.02725	0.0355	L0.01
**Cd**	0.00001285	0.00001435	0.00001295	0.00003265	0.00000975	0.0000084	0.0000139	0.0000368
**Ca**	26.8	28.55	31	16.85	27.45	28.9	30.675	13.95
**C (organic)**	9.825	8.5	8.7	9.565	8.788333333	9.8025	9.1225	10.925
**C (inorganic)**	18.95	18.35	19.3	11.155	17.7	19.9	20.5875	9.45
**C (total)**	28.775	26.85	28	20.7	26.46666667	29.65	29.725	20.35
**Cs**	0.000036	0.0000615	0.0000465	0.0425	0.02775	0.05325	0.08075	0.1585
**Cl**	10.75	11.2	12.55	2.72	10.2025	10.8	9.7725	2.435
**Cr**	0.00032	0.00071	0.000875	0.000215	0.0002725	0.0005825	0.0009375	0.000475
**Co**	0.000125	0.00024	0.000195	L0.1	0.135	0.2075	0.285	L0.1
**Cu**	0.00175	0.002145	0.00212	0.00137	0.00186	0.0019025	0.00234	0.00158
**Fe**	0.1685	0.45	0.326	0.062	0.135	0.3175	0.47975	0.2285
**K**	2.98	3.6	3.795	1.067	3.8225	3.985	4.0925	1.106
**Li**	0.01295	0.01315	0.01775	0.00231	0.0127	0.013825	0.01585	0.002055
**Mg**	14.2	15.95	17.1	5.54	13.9	14.175	15.4	4.705
**Mn**	0.02705	0.0337	0.02735	0.003105	0.0192	0.0422	0.024825	0.006015
**Hg**	N/A	N/A	N/A	L0.000005	0.000925	0.0011875	0.00126125	L0.000005
**Mo**	0.0007175	0.0008535	0.0010595	0.000222	0.000791	0.0009255	0.00100125	0.0002005
**Ni**	0.00156	0.002135	0.00184	0.00077	0.0019475	0.00207	0.0020675	0.00096
**N**	0.44	0.5725	0.696666667	0.67	0.506666667	0.6175	0.84125	0.585
**N (Kjeldahl)**	0.4275	0.46	0.656666667	0.515	0.495	0.5825	0.7525	0.5
**NH_3**	0.011	0.0185	0.015666667	L0.01	L0.01	0.01625	0.036375	L0.01
**NO_2+NO_3**	0.0197	0.111075	0.0423	0.1555	0.01105	0.0366	0.091125	0.08355
**Na**	14.8	15.4	17.45	3.475	13.75	13.9	13.725	3.205
**O**	10.5	8.45	11.1	14.4	9.75	7.3	9.5	16.3
**P**	0.0569	0.093925	0.111733333	0.02545	0.064783333	0.10085	0.135375	0.0232
**P (dissolved)**	0.0359	0.06775	0.088533333	0.02	0.04735	0.074375	0.105425	0.0164
**P (inorganic)**	0.04665	0.0807	0.1012	0.0184	0.06505	0.0837625	0.120125	0.0181
**P (particulate)**	0.018	0.02905	0.0276	0.00545	0.019075	0.031125	0.047275	0.00675
**P (reactive)**	0.030475	0.0617	0.082966667	0.01485	0.03215	0.0574	0.103775	0.01375
**P (acid hydrolizable)**	0.01615	0.019	0.0184	L0.0042	0.0329	0.026375	0.017525	0.00765
**Pb**	0.0001105	0.000227	0.000278	0.000074	0.000101	0.00017375	0.00031525	0.0001955
**Rb**	0.001705	0.00244	0.00228	0.00159	0.00155	0.00207	0.002655	0.001755
**Sb**	0.00011	0.00013	0.000185	0.00026	0.00013	0.00014	0.0001775	0.0001
**Se**	0.000234	0.000244	0.0002945	0.0001185	0.00021275	0.00022725	0.0002935	0.0001215
**Si**	2.27	4.565	5.34	2.585	0.8175	3.3125	7.525	2.05
**Sr**	0.089	0.0927	0.111	0.03275	0.088475	0.0966	0.10735	0.03075
**Sn**	L0.0001	L0.0001	L0.0001	L0.0001	L0.0001	L0.0001	0.0001325	0.00013
**SO_4**	46.45	51.15	60.5	4.86	43.225	52.25	56.5	4.615
**Te**	L0.0002	L0.0002	L0.0002	L0.0002	L0.0002	L0.0002	L0.0002	L0.0002
**Tl**	L0.00001	0.000013	0.000011	L0.00001	L0.00001	0.00001	0.000014	L0.00001
**Th**	L0.0001	L0.0001	L0.0001	L0.0001	L0.0001	L0.0001	0.0001425	L0.0001
**Ti**	0.00464	0.01244	0.008565	0.00143	0.0035525	0.0083225	0.018635	0.004565
**U**	0.001125	0.001085	0.00141	0.0001365	0.00105725	0.001355	0.00146	0.0001385
**V**	0.001575	0.002815	0.00299	0.00064	0.001995	0.0025025	0.004325	0.00075
**Zn**	L0.003	L0.003	0.00335	0.0067	L0.003	L0.003	0.005925	0.0091
**Zr**	0.000222	0.0003375	0.0003395	0.000123	0.000188	0.0002975	0.000795	0.000205

L, Less than the detection limit listed

Table S3. Media composition used to cultivate different groups of microorganisms. Values are listed as g/L unless otherwise noted. All were adjusted to pH 5.9 prior to autoclaving.

Component	RO	OM	BG-11	PNSM	PM	DM
$MgCl_2$	-	-	-	0.5	-	-
$MgSO_4$	0.50	0.50	0.0750	-	-	0.50
KH_2PO_4	0.30	0.30	0.0300	0.3	-	0.30
NH_4Cl	0.30	0.30	-	0.3	-	0.30
$NaNO_3$	-	-	0.1500	-	-	-
KCl	0.30	0.30	-	-	-	-
$CaCl_2$	0.05	0.05	-	0.1	-	0.10
Na_2CO_3	-	-	0.0200	-	-	-
NaAcetate	1.00	**0.10**	-	1.0	0.50	-
NaCitrate	-	-	0.0060	-	-	-
Malic Acid	-	-	-	0.3	-	-
Na_2CO_3	-	-	0.0200	-	-	-
Na_2-EDTA	-	-	0.0001	-	-	-
Ferric Ammonium Citrate	-	-	0.0060	-	-	-
Potato Extract*	-	-	-	-	*	-
Yeast Extract	1.00	**0.05**	-	0.2	1.00	-
Bacto Peptone	0.50	-	-	-	-	-
Casamino Acids	0.50	**0.05**	-	-	-	-
Trace Elements	2 mL	2 mL	2 mL	2 mL	2 mL	-
Vitamins	2 mL	2 mL	2 mL	2 mL	2 mL	-
Added after autoclaving:						
Cystine (0.3mM)	-	-	-	5 mL	-	-
Methionine (0.3mM)	-	-	-	5 mL	-	-
ph adjustments	**A**	**A**	**B**	**C**	**B**	**D**

A, 4.0uL/mL 0.5N NaOH added after autoclaving to achieve 7.5 pH
B, Self-adjusts to pH 7.5 during autoclaving process
C, 3.0uL/mL 0.5N NaOH added after autoclaving to achieve 7.5 pH
D, 2.4uL/mL 0.5N NaOH added after autoclaving to achieve 7.5 pH

* 1L of water per kg of peeled potatoes (organic better), cut in halves or quarters depending on size, enough water to cover all by 1-2 cm. Boiled for 1 h, stained water through cotton and dilute to twice volume.

Trace Elements	g/L
$FeSO_4$	0.300
$MnCl_2$	0.003
$CoCl_2$	0.005
$CuCl_2$	0.001
$NiCl_2$	0.002
Na_2MoO_4	0.003
$ZnSO_4$	0.005
H_3BO_3	0.002

In 0.5L Adjust to **3.0**pH
Fill to 1L and refrigerate

Essential Vitamins	g/L
Nicotinic Acid	0.40
Thiamin	0.40
Biotin	0.08
B_{12}	0.02

Filter sterilize solution and refrigerate

Table S4 (1/7). Enumeration data from all individual sites and seasons. Bacterial counts from liquid and sediment listed as means with standard deviation of triplicates with CFU/mL or CFU/g, respectfully.

				1		2		3		4	
Year	Season	Medium		liquid	sediment	liquid	sediment	liquid	sediment	liquid	sediment
2016	**Spring**	RO	Total	$6.55\pm0.75\times10^{3}$	$2.11\pm0.54\times10^{5}$	$3.40\pm0.40\times10^{3}$	$1.60\pm0.07\times10^{4}$	$1.01\pm0.22\times10^{4}$	$1.42\pm0.03\times10^{5}$	$5.10\pm1.00\times10^{3}$	$3.45\pm0.15\times10^{3}$
			Pigmented	$1.90\pm0.40\times10^{3}$	$2.40\pm2.30\times10^{4}$	$1.40\pm0.20\times10^{3}$	$3.55\pm0.05\times10^{3}$	$1.85\pm0.45\times10^{3}$	$2.95\pm0.45\times10^{4}$	$3.00\pm0.00\times10^{2}$	$1.50\pm0.20\times10^{3}$
			AAP	2.00×10^{2}	1.17×10^{4}	3.77×10^{2}	1.08×10^{3}	4.32×10^{2}	1.12×10^{4}	8.68×10^{1}	1.33×10^{3}
		OM	Total	$7.50\pm0.70\times10^{4}$	$1.74\pm0.47\times10^{6}$	$4.25\pm1.75\times10^{4}$	$1.31\pm0.42\times10^{5}$	$2.85\pm0.46\times10^{4}$	$4.85\pm0.75\times10^{5}$	N/A	$4.65\pm1.65\times10^{5}$
			Pigmented	$4.50\pm1.50\times10^{3}$	$2.85\pm0.75\times10^{5}$	$8.00\pm1.00\times10^{3}$	$2.80\pm1.00\times10^{4}$	$4.30\pm1.30\times10^{3}$	$9.00\pm2.00\times10^{4}$	$1.90\pm0.40\times10^{3}$	$5.00\pm1.00\times10^{4}$
			AAP	7.50×10^{2}	3.29×10^{4}	4.44×10^{2}	2.47×10^{4}	3.25×10^{2}	3.46×10^{3}	4.75×10^{1}	1.43×10^{4}
		RO_T	Total	$2.00\pm1.00\times10^{1}$	$2.31\pm0.36\times10^{3}$	$5.00\pm0.00\times10^{1}$	$4.00\pm0.00\times10^{1}$	$1.30\pm0.00\times10^{2}$	$4.70\pm0.00\times10^{3}$	$4.00\pm0.00\times10^{1}$	$7.60\pm0.00\times10^{2}$
			Pigmented	$1.50\pm0.50\times10^{1}$	$1.87\pm0.17\times10^{3}$	$4.00\pm0.00\times10^{1}$	$4.00\pm0.00\times10^{1}$	$1.30\pm0.00\times10^{2}$	$1.50\pm0.00\times10^{3}$	$3.00\pm0.00\times10^{1}$	$5.10\pm0.00\times10^{2}$
			AAP	0	6.57×10^{2}	0	3.79×10^{1}	1.08×10^{1}	1.00×10^{3}	9.00×10^{0}	1.52×10^{2}
		Potato	Total	$8.90\pm0.00\times10^{2}$	$5.45\pm1.45\times10^{4}$	$9.10\pm0.00\times10^{2}$	$3.50\pm0.00\times10^{3}$	$2.75\pm0.00\times10^{3}$	$4.60\pm0.00\times10^{4}$	$1.64\pm0.00\times10^{3}$	$8.91\pm0.00\times10^{3}$
			Pigmented	$2.60\pm0.10\times10^{2}$	$1.35\pm0.75\times10^{4}$	$3.20\pm0.00\times10^{2}$	$1.00\pm0.00\times10^{3}$	$3.30\pm0.00\times10^{2}$	$6.00\pm0.00\times10^{3}$	$2.30\pm0.00\times10^{2}$	$2.80\pm0.00\times10^{2}$
			AAP	1.73×10^{1}	3.46×10^{2}	3.37×10^{1}	5.00×10^{1}	6.60×10^{1}	3.53×10^{2}	2.56×10^{1}	6.53×10^{1}
		BG-11	Total	$6.45\pm0.25\times10^{4}$	$7.50\pm1.50\times10^{5}$	$7.65\pm0.25\times10^{4}$	$4.16\pm1.98\times10^{4}$	$1.70\pm1.41\times10^{4}$	$3.02\pm0.14\times10^{5}$	$5.15\pm1.25\times10^{4}$	$3.25\pm0.15\times10^{5}$
			AOP	$1.50\pm1.50\times10^{1}$	$4.60\pm0.60\times10^{2}$	$8.50\pm5.50\times10^{1}$	$1.00\pm0.30\times10^{3}$	$6.00\pm2.00\times10^{2}$	$2.05\pm0.05\times10^{4}$	$5.00\pm5.00\times10^{1}$	$5.00\pm5.00\times10^{2}$
		PNSm	Total	$4.35\pm2.25\times10^{2}$	$7.40\pm0.00\times10^{3}$	$2.40\pm0.00\times10^{2}$	$9.10\pm0.00\times10^{2}$	$1.70\pm0.00\times10^{2}$	$6.30\pm0.00\times10^{3}$	$9.00\pm0.00\times10^{1}$	$8.20\pm0.00\times10^{2}$
			AnAnP	0	0	0	0	0	0	0	0
	**Summer**	RO	Total	$5.95\pm0.85\times10^{4}$	$2.80\pm0.80\times10^{5}$	$4.85\pm0.35\times10^{3}$	$5.15\pm0.55\times10^{6}$	$6.65\pm0.55\times10^{3}$	$9.60\pm2.20\times10^{3}$	$7.15\pm3.75\times10^{3}$	$1.37\pm0.04\times10^{4}$
			Pigmented	$7.50\pm1.50\times10^{3}$	$8.50\pm6.50\times10^{4}$	$1.50\pm0.30\times10^{3}$	$1.80\pm1.00\times10^{6}$	$3.25\pm1.15\times10^{3}$	$3.95\pm0.35\times10^{3}$	$5.05\pm2.15\times10^{3}$	$1.95\pm0.25\times10^{3}$
			AAP	1.32×10^{3}	1.23×10^{4}	3.46×10^{2}	8.00×10^{4}	8.92×10^{2}	7.29×10^{2}	1.36×10^{3}	3.78×10^{2}
		OM	Total	$4.05\pm0.45\times10^{5}$	$5.05\pm0.15\times10^{5}$	$4.05\pm1.45\times10^{4}$	$6.10\pm0.30\times10^{7}$	$2.02\pm1.95\times10^{4}$	$4.80\pm0.40\times10^{4}$	$1.37\pm0.01\times10^{4}$	$8.00\pm1.00\times10^{4}$
			Pigmented	$1.50\pm0.30\times10^{5}$	$1.25\pm0.65\times10^{5}$	$1.75\pm0.95\times10^{4}$	$2.35\pm0.55\times10^{7}$	$2.05\pm0.25\times10^{3}$	$1.30\pm0.70\times10^{4}$	$5.30\pm1.80\times10^{3}$	$2.30\pm0.30\times10^{4}$
			AAP	1.11×10^{4}	1.39×10^{4}	1.22×10^{3}	0	1.58×10^{2}	5.80×10^{2}	4.16×10^{2}	2.17×10^{3}
		RO_T	Total	$1.55\pm0.00\times10^{3}$	$3.10\pm0.00\times10^{3}$	$8.70\pm0.00\times10^{2}$	$2.41\pm0.00\times10^{4}$	$2.60\pm0.00\times10^{2}$	$9.60\pm0.00\times10^{2}$	$2.30\pm0.00\times10^{2}$	$5.30\pm0.00\times10^{2}$
			Pigmented	$1.39\pm0.00\times10^{3}$	$3.10\pm0.00\times10^{3}$	$8.70\pm0.00\times10^{2}$	$2.41\pm0.00\times10^{4}$	$3.10\pm0.00\times10^{2}$	$8.10\pm0.00\times10^{2}$	$2.30\pm0.00\times10^{2}$	$4.90\pm0.00\times10^{2}$
			AAP	0	0	3.00×10^{1}	0	2.21×10^{1}	9.95×10^{1}	4.26×10^{1}	0
		Potato	Total	$7.90\pm0.00\times10^{3}$	$4.40\pm0.00\times10^{4}$	$4.00\pm0.00\times10^{2}$	$2.41\pm0.00\times10^{4}$	$1.05\pm0.00\times10^{3}$	$2.24\pm0.00\times10^{3}$	$1.60\pm0.00\times10^{2}$	$9.10\pm0.00\times10^{3}$
			Pigmented	$2.70\pm0.00\times10^{3}$	$6.00\pm0.00\times10^{3}$	$1.90\pm0.00\times10^{2}$	$2.41\pm0.00\times10^{4}$	$3.70\pm0.00\times10^{2}$	$1.02\pm0.00\times10^{3}$	$8.00\pm0.00\times10^{1}$	$1.00\pm0.00\times10^{3}$
			AAP	6.75×10^{2}	7.50×10^{2}	3.45×10^{1}	0	0	9.71×10^{1}	0	0
		BG-11	Total	$1.07\pm0.00\times10^{6}$	$7.50\pm0.00\times10^{5}$	$5.50\pm0.00\times10^{5}$	$4.10\pm0.00\times10^{5}$	$9.00\pm0.00\times10^{5}$	$1.49\pm0.00\times10^{5}$	$7.20\pm0.00\times10^{3}$	$1.58\pm0.00\times10^{5}$
			AOP	$2.20\pm0.00\times10^{3}$	$2.20\pm0.00\times10^{3}$	$3.70\pm0.00\times10^{2}$	$2.00\pm0.00\times10^{4}$	$1.40\pm0.00\times10^{4}$	$4.90\pm0.00\times10^{2}$	$4.60\pm0.00\times10^{2}$	$5.00\pm0.00\times10^{2}$
		PNSm	Total	$1.88\pm0.00\times10^{3}$	$1.27\pm0.00\times10^{3}$	$9.80\pm0.00\times10^{2}$	$4.40\pm0.00\times10^{4}$	$1.20\pm0.00\times10^{2}$	$1.90\pm0.00\times10^{2}$	$8.40\pm0.00\times10^{2}$	$4.50\pm0.00\times10^{3}$
			AnAnP	$3.00\pm0.00\times10^{1}$	0	0	0	0	0	0	0
	**Fall**	RO	Total	$7.75\pm0.35\times10^{3}$	$2.44\pm0.14\times10^{4}$	$1.08\pm0.13\times10^{4}$	$5.35\pm0.35\times10^{3}$	$8.10\pm3.40\times10^{3}$	$8.95\pm0.25\times10^{3}$	$6.65\pm1.45\times10^{2}$	$1.07\pm0.55\times10^{5}$
			Pigmented	$2.75\pm0.45\times10^{3}$	$7.50\pm0.50\times10^{3}$	$3.00\pm0.20\times10^{3}$	$1.15\pm0.45\times10^{3}$	$1.30\pm0.10\times10^{3}$	$2.10\pm0.60\times10^{3}$	$2.65\pm0.65\times10^{2}$	$2.50\pm0.50\times10^{4}$
			AAP	2.43×10^{2}	4.69×10^{2}	4.44×10^{2}	3.38×10^{1}	1.63×10^{2}	1.17×10^{2}	5.68×10^{1}	0
		OM	Total	$5.95\pm0.65\times10^{4}$	$2.06\pm0.15\times10^{5}$	$4.62\pm0.35\times10^{4}$	$7.90\pm0.10\times10^{4}$	$1.80\pm0.09\times10^{4}$	$1.31\pm0.19\times10^{4}$	2.18 ± 0.1510^{3}	$2.04\pm0.24\times10^{6}$
			Pigmented	$4.50\pm1.50\times10^{3}$	$1.80\pm0.30\times10^{4}$	$4.00\pm0.10\times10^{3}$	$5.00\pm1.00\times10^{3}$	$1.50\pm0.20\times10^{3}$	$1.10\pm0.30\times10^{3}$	$5.00\pm0.30\times10^{2}$	$5.50\pm0.50\times10^{5}$
			AAP	5.63×10^{2}	0	1.74×10^{2}	2.38×10^{2}	9.38×10^{1}	7.33×10^{1}	4.55×10^{1}	0
		RO_T	Total	$3.80\pm0.00\times10^{2}$	$5.00\pm0.00\times10^{3}$	$1.18\pm0.00\times10^{3}$	$4.00\pm0.00\times10^{2}$	$1.10\pm0.00\times10^{2}$	$9.80\pm0.00\times10^{2}$	$6.00\pm0.00\times10^{1}$	$1.00\pm0.00\times10^{4}$
			Pigmented	$3.80\pm0.00\times10^{2}$	$4.50\pm0.00\times10^{3}$	$1.18\pm0.00\times10^{3}$	$3.40\pm0.00\times10^{2}$	$1.10\pm0.00\times10^{2}$	$9.00\pm0.00\times10^{2}$	$6.00\pm0.00\times10^{1}$	$1.00\pm0.00\times10^{4}$
			AAP	7.60×10^{1}	1.23×10^{3}	1.18×10^{2}	6.58×10^{1}	0	2.25×10^{2}	5.00×10^{0}	0
		Potato	Total	$5.90\pm0.00\times10^{2}$	$2.40\pm0.00\times10^{4}$	$4.50\pm0.00\times10^{3}$	$1.22\pm0.00\times10^{3}$	$6.60\pm0.00\times10^{2}$	$2.40\pm0.00\times10^{4}$	$2.00\pm0.00\times10^{1}$	$2.89\pm0.00\times10^{6}$
			Pigmented	$2.00\pm0.00\times10^{2}$	$1.70\pm0.00\times10^{4}$	$1.70\pm0.00\times10^{3}$	$9.00\pm0.00\times10^{2}$	$2.20\pm0.00\times10^{2}$	$4.00\pm0.00\times10^{3}$	0	$8.60\pm0.00\times10^{5}$
			AAP	0	0	0	0	0	0	0	0
		BG-11	Total	$2.02\pm0.00\times10^{3}$	$1.02\pm0.00\times10^{5}$	$2.50\pm0.00\times10^{5}$	$2.17\pm0.00\times10^{4}$	$4.00\pm0.20\times10^{4}$	$9.20\pm0.20\times10^{4}$	$5.80\pm0.80\times10^{4}$	$1.35\pm0.28\times10^{5}$
			AOP	$1.00\pm0.00\times10^{1}$	$2.00\pm0.00\times10^{2}$	$2.70\pm0.00\times10^{2}$	$1.70\pm0.00\times10^{2}$	$7.00\pm5.00\times10^{1}$	$1.80\pm0.10\times10^{2}$	$1.00\pm0.00\times10^{1}$	$5.50\pm1.50\times10^{1}$
		PNSm	Total	$3.10\pm0.00\times10^{2}$	$7.00\pm0.00\times10^{2}$	$3.50\pm0.00\times10^{2}$	$1.70\pm0.00\times10^{2}$	$6.00\pm0.00\times10^{1}$	$2.20\pm0.00\times10^{2}$	$2.00\pm0.00\times10^{1}$	$1.74\pm0.00\times10^{3}$
			AnAnP	$2.00\pm0.00\times10^{1}$	$5.00\pm0.00\times10^{1}$	0	0	0	0	0	0
2017	**Spring**	RO	Total	$7.63\pm2.10\times10^{3}$	$8.67\pm0.80\times10^{4}$	$1.71\pm0.27\times10^{4}$	$1.84\pm0.00\times10^{4}$	$2.20\pm0.04\times10^{4}$	$5.47\pm0.90\times10^{5}$	$1.53\pm0.05\times10^{4}$	$9.40\pm0.75\times10^{5}$
			Pigmented	$2.27\pm0.45\times10^{3}$	$4.33\pm1.85\times10^{4}$	$1.83\pm0.15\times10^{3}$	$2.17\pm0.40\times10^{3}$	$5.40\pm1.00\times10^{3}$	$1.09\pm0.04\times10^{4}$	$1.09\pm0.04\times10^{4}$	$3.37\pm0.70\times10^{5}$
			AAP	1.62×10^{2}	4.48×10^{3}	0	9.85×10^{1}	2.08×10^{2}	2.73×10^{3}	2.73×10^{3}	1.02×10^{5}
		OM	Total	$1.63\pm0.33\times10^{4}$	$2.20\pm0.27\times10^{5}$	$6.93\pm1.20\times10^{4}$	$1.63\pm0.30\times10^{4}$	$5.79\pm0.16\times10^{4}$	$8.77\pm0.10\times10^{5}$	$2.49\pm0.05\times10^{4}$	$1.91\pm0.25\times10^{6}$
			Pigmented	$4.40\pm0.85\times10^{3}$	$5.05\pm0.25\times10^{4}$	$1.10\pm0.65\times10^{4}$	$4.70\pm1.70\times10^{3}$	$7.83\pm0.90\times10^{3}$	$4.33\pm0.65\times10^{5}$	$1.19\pm0.13\times10^{4}$	$3.40\pm0.00\times10^{5}$
			AAP	5.50×10^{2}	0	5.50×10^{2}	2.14×10^{2}	5.11×10^{3}	1.63×10^{5}	6.78×10^{3}	8.50×10^{4}
		RO_T	Total	$6.67\pm0.00\times10^{0}$	$2.47\pm1.70\times10^{2}$	$6.23\pm0.95\times10^{2}$	$5.43\pm0.25\times10^{2}$	$4.47\pm2.25\times10^{2}$	$2.67\pm0.55\times10^{2}$	$1.40\pm0.15\times10^{2}$	$1.07\pm0.55\times10^{3}$
			Pigmented	$3.33\pm5.00\times10^{0}$	$2.37\pm1.55\times10^{2}$	$4.90\pm0.65\times10^{2}$	$4.87\pm0.35\times10^{2}$	$3.73\pm3.20\times10^{2}$	$4.27\pm0.30\times10^{2}$	$1.23\pm0.10\times10^{2}$	$8.33\pm3.00\times10^{2}$
			AAP	0	1.18×10^{2}	2.45×10^{2}	3.35×10^{2}	1.87×10^{2}	1.42×10^{2}	5.29×10^{1}	7.69×10^{2}
		Potato	Total	$6.80\pm0.45\times10^{3}$	$1.08\pm0.13\times10^{4}$	$2.13\pm0.30\times10^{3}$	$1.00\pm0.09\times10^{5}$	$5.97\pm0.85\times10^{3}$	$2.83\pm0.30\times10^{3}$	$6.10\pm1.40\times10^{3}$	$3.07\pm0.05\times10^{4}$
			Pigmented	$8.40\pm0.70\times10^{3}$	$1.38\pm0.09\times10^{4}$	$4.57\pm0.65\times10^{3}$	$1.47\pm0.04\times10^{5}$	$2.12\pm0.81\times10^{4}$	$3.07\pm0.20\times10^{4}$	$2.45\pm0.06\times10^{4}$	$3.37\pm0.10\times10^{4}$
			AAP	1.20×10^{3}	5.52×10^{2}	4.57×10^{2}	3.50×10^{4}	1.18×10^{3}	5.12×10^{3}	5.52×10^{3}	3.37×10^{3}
		BG-11	Total	$1.58\pm0.12\times10^{4}$	$1.42\pm0.10\times10^{5}$	$1.25\pm0.00\times10^{5}$	$1.80\pm0.00\times10^{5}$	$1.42\pm0.03\times10^{5}$	$1.10\pm0.04\times10^{6}$	$3.09\pm0.40\times10^{4}$	$1.75\pm0.21\times10^{6}$
			AOP	$3.33\pm5.00\times10^{0}$	$3.93\pm0.60\times10^{2}$	$9.85\pm9.75\times10^{3}$	$1.45\pm0.05\times10^{3}$	$2.23\pm0.45\times10^{2}$	$1.83\pm0.10\times10^{4}$	$9.00\pm0.00\times10^{1}$	$2.89\pm3.00\times10^{3}$
		PNSm	Total	$9.67\pm3.50\times10^{1}$	$4.00\pm0.80\times10^{2}$	$9.70\pm1.20\times10^{2}$	$1.66\pm1.04\times10^{3}$	$9.37\pm1.10\times10^{2}$	$2.17\pm0.44\times10^{3}$	$1.77\pm0.05\times10^{2}$	$1.76\pm0.24\times10^{4}$
			AnAnP	$3.33\pm5.00\times10^{0}$	$7.00\pm3.50\times10^{1}$	$3.00\pm1.50\times10^{1}$	$2.73\pm2.10\times10^{2}$	0	$6.73\pm0.30\times10^{2}$	$3.33\pm5.00\times10^{0}$	$5.93\pm5.35\times10^{3}$

Table S4. Continued (2/7).

Year	Season	Medium		1 liquid	1 sediment	2 liquid	2 sediment	3 liquid	3 sediment	4 liquid	4 sediment
2017	**Summer**	RO	Total	6.30±0.50×10^{3}	8.67±0.50×10^{4}	4.00±0.00×10^{4}	4.27±0.55×10^{6}	1.27±0.04×10^{3}	4.03±0.15×10^{5}	6.53±0.15×10^{3}	4.75±0.00×10^{4}
			Pigmented	6.30±0.50×10^{3}	1.80±0.35×10^{4}	4.10±0.00×10^{4}	7.00±1.50×10^{2}	3.00±0.15×10^{2}	1.20±0.15×10^{5}	1.87±0.10×10^{3}	1.35±0.00×10^{4}
			AAP	6.67×10^{1}	0	0	4.67×10^{4}	3.53×10^{1}	1.55×10^{4}	2.67×10^{2}	3.21×10^{3}
		OM	Total	3.80±0.95×10^{5}	1.14±0.09×10^{6}	1.00±0.05×10^{5}	5.83±2.05×10^{6}	1.51±0.12×10^{4}	9.83±2.30×10^{5}	7.87±0.10×10^{4}	1.74±0.45×10^{5}
			Pigmented	2.90±0.30×10^{3}	1.93±0.20×10^{5}	5.87±1.35×10^{4}	7.00±3.00×10^{5}	2.40±1.20×10^{3}	1.13±0.45×10^{5}	6.33±2.50×10^{3}	1.43±0.45×10^{4}
			AAP	0	0	1.43×10^{3}	5.83×10^{4}	1.41×10^{2}	3.97×10^{4}	4.22×10^{2}	8.96×10^{2}
		RO_T	Total	9.33±0.00×10^{1}	1.24±0.21×10^{3}	3.23±0.10×10^{2}	2.13±3.05×10^{4}	2.00±0.00×10^{1}	6.03±0.39×10^{3}	5.20±0.25×10^{2}	6.73±0.05×10^{2}
			Pigmented	9.00±0.50×10^{1}	1.24±0.21×10^{3}	3.23±0.10×10^{2}	2.11±0.30×10^{4}	1.00±0.00×10^{1}	5.00±3.25×10^{3}	4.30±0.20×10^{2}	4.07±0.05×10^{2}
			AAP	1.50×10^{1}	3.54×10^{2}	0	1.40×10^{4}	0	1.43×10^{3}	0	2.71×10^{2}
		Potato	Total	2.40±0.71×10^{3}	2.34±0.24×10^{4}	1.53±0.12×10^{4}	1.36±0.17×10^{6}	3.53±0.05×10^{3}	3.43±0.05×10^{5}	9.23±2.00×10^{3}	4.03±0.50×10^{4}
			Pigmented	3.43±1.40×10^{2}	3.17±0.20×10^{3}	5.57±2.10×10^{3}	2.03±0.60×10^{5}	1.43±0.20×10^{3}	8.67±1.00×10^{4}	5.37±3.20×10^{3}	1.57±0.10×10^{4}
			AAP	0	0	0	1.20×10^{4}	6.83×10^{1}	8.39×10^{3}	2.98×10^{2}	0
		BG-11	Total	5.80±1.00×10^{5}	2.71±0.38×10^{5}	1.14±0.31×10^{5}	6.53±0.75×10^{6}	2.15±0.03×10^{4}	1.51±0.15×10^{6}	1.01±0.02×10^{5}	1.70±0.11×10^{5}
			AOP	0	0	4.60±2.10×10^{4}	1.10±0.15×10^{5}	3.33±5.00×10^{1}	0	1.47±0.45×10^{2}	1.00±0.15×10^{3}
		PNSm	Total	2.50±0.20×10^{2}	1.74±0.18×10^{3}	1.36±0.10×10^{3}	9.00±0.50×10^{4}	7.00±0.00×10^{1}	1.65±0.53×10^{3}	1.08±0.30×10^{3}	4.13±0.20×10^{3}
			AnAnP	0	0	0	0	0	0	0	1.33±2.00×10^{1}
	**Fall**	RO	Total	1.81±0.12×10^{4}	5.35±0.50×10^{5}	1.58±0.10×10^{4}	9.91±1.53×10^{4}	2.08±0.01×10^{4}	3.01±0.12×10^{5}	8.90±2.45×10^{3}	1.36±0.27×10^{6}
			Pigmented	4.23±0.25×10^{3}	1.83±0.28×10^{5}	1.25±0.13×10^{4}	7.44±1.17×10^{4}	8.73±0.15×10^{3}	1.14±0.00×10^{5}	4.40±0.30×10^{3}	5.97±1.10×10^{5}
			AAP	2.65×10^{2}	0	3.38×10^{2}	0	3.49×10^{2}	1.63×10^{4}	2.10×10^{2}	2.44×10^{4}
		OM	Total	1.38±0.13×10^{5}	6.75±0.15×10^{5}	1.83±0.19×10^{4}	2.80±0.00×10^{5}	2.80±0.11×10^{4}	1.06±0.11×10^{6}	1.17±0.14×10^{4}	6.13±0.15×10^{6}
			Pigmented	2.47±0.70×10^{4}	1.12±0.35×10^{5}	8.53±0.55×10^{3}	7.33±0.00×10^{4}	8.47±1.80×10^{3}	4.21±0.50×10^{5}	2.87±0.20×10^{3}	1.93±0.15×10^{6}
			AAP	7.47×10^{2}	3.60×10^{3}	0	2.53×10^{3}	0	3.83×10^{4}	1.79×10^{2}	0
		RO_T	Total	8.33±1.00×10^{1}	5.80±0.33×10^{3}	3.67±0.50×10^{1}	1.76±0.10×10^{3}	9.00±5.00×10^{1}	8.14±0.79×10^{2}	8.00±2.00×10^{1}	6.53±0.90×10^{3}
			Pigmented	8.33±1.00×10^{1}	5.48±0.45×10^{3}	3.67±0.50×10^{1}	1.62±0.03×10^{3}	9.00±5.00×10^{1}	7.14±0.14×10^{2}	7.67±1.50×10^{1}	5.27±0.95×10^{3}
			AAP	1.19×10^{2}	4.98×10^{2}	9.17×10^{0}	5.41×10^{2}	1.13×10^{1}	2.60×10^{2}	7.69×10^{1}	2.15×10^{3}
		Potato	Total	1.42±0.00×10^{4}	3.17±0.23×10^{5}	8.80±0.60×10^{3}	9.27±0.10×10^{4}	9.93±1.05×10^{3}	3.08±0.00×10^{4}	1.48±0.05×10^{3}	8.93±0.80×10^{5}
			Pigmented	4.25±0.00×10^{3}	1.33±0.20×10^{5}	6.10±0.00×10^{3}	7.67±0.13×10^{4}	5.33±0.90×10^{3}	1.49±0.00×10^{4}	8.50±0.15×10^{2}	4.00±0.75×10^{5}
			AAP	1.33×10^{2}	0	0	0	2.05×10^{2}	5.50×10^{2}	4.25×10^{1}	5.22×10^{4}
		BG-11	Total	2.02±0.17×10^{4}	6.75±0.15×10^{5}	1.80±0.37×10^{5}	1.49±0.05×10^{5}	1.33±0.11×10^{5}	7.76±0.86×10^{5}	8.10±0.30×10^{4}	1.17±0.00×10^{5}
			AOP	0.67±1.00×10^{2}	6.67±0.25×10^{3}	1.33±0.40×10^{4}	1.00±0.17×10^{3}	2.73±0.60×10^{4}	3.81±0.71×10^{1}	5.00±0.25×10^{1}	1.50±0.65×10^{2}
		PNSm	Total	8.83±3.85×10^{2}	1.07±0.21×10^{4}	1.40±0.10×10^{2}	1.84±0.10×10^{3}	4.00±1.00×10^{1}	6.29±0.00×10^{2}	7.33±2.50×10^{1}	6.17±0.30×10^{4}
			AnAnP	0	5.00±5.00×10^{1}	0	0	0	0	0	1.00±0.50×10^{1}
2018	**Spring**	RO	Total	6.47±3.85×10^{3}	1.37±0.20×10^{5}	4.53±0.30×10^{4}	1.13±0.18×10^{5}	1.38±0.02×10^{3}	6.05±0.00×10^{4}	3.33±1.75×10^{2}	2.97±1.10×10^{5}
			Pigmented	2.67±1.20×10^{4}	5.00±0.50×10^{4}	1.30±0.45×10^{5}	4.87±1.60×10^{4}	3.87±0.30×10^{3}	2.07±1.60×10^{4}	1.50±1.10×10^{3}	6.23±0.15×10^{4}
			AAP	4.64×10^{3}	4.63×10^{3}	2.60×10^{4}	2.05×10^{4}	1.10×10^{3}	2.02×10^{3}	8.33×10^{1}	1.47×10^{4}
		OM	Total	1.11±0.56×10^{4}	2.00±0.90×10^{5}	6.87±1.40×10^{4}	1.60±0.01×10^{5}	3.00±0.00×10^{4}	1.35±0.08×10^{5}	2.59±0.72×10^{3}	3.70±0.20×10^{5}
			Pigmented	3.83±2.65×10^{4}	4.67±1.50×10^{4}	7.00±1.00×10^{4}	4.93±0.25×10^{4}	3.00±0.00×10^{4}	1.83±0.25×10^{4}	4.00±0.70×10^{3}	5.03±0.20×10^{4}
			AAP	0	1.67×10^{3}	5.71×10^{3}	0	2.73×10^{3}	3.67×10^{3}	4.44×10^{2}	8.39×10^{3}
		RO_T	Total	8.00±2.00×10^{1}	4.43±0.35×10^{2}	6.63±3.80×10^{2}	1.46±0.95×10^{3}	5.33±3.50×10^{1}	1.20±0.45×10^{2}	3.00±1.50×10^{1}	3.00±0.58×10^{3}
			Pigmented	8.00±2.00×10^{1}	2.23±0.00×10^{2}	5.77±3.60×10^{2}	1.22±0.75×10^{3}	2.67±0.50×10^{1}	9.67±2.50×10^{1}	3.00±1.50×10^{1}	3.00±0.58×10^{3}
			AAP	0	1.49×10^{2}	2.88×10^{2}	8.59×10^{2}	7.06×10^{0}	9.67×10^{1}	0	2.18×10^{3}
		Potato	Total	5.53±0.75×10^{2}	8.33±1.30×10^{2}	5.17±0.25×10^{2}	7.30±7.60×10^{3}	1.74±0.22×10^{3}	1.10±0.00×10^{4}	2.25±1.04×10^{3}	3.60±1.15×10^{3}
			Pigmented	4.33±1.10×10^{2}	4.60±0.45×10^{2}	2.37±0.50×10^{2}	4.43±4.65×10^{3}	5.37±1.10×10^{2}	2.50±0.00×10^{3}	4.90±2.95×10^{2}	1.17±0.04×10^{3}
			AAP	8.46×10^{1}	6.97×10^{1}	1.23×10^{2}	2.55×10^{3}	3.22×10^{2}	1.07×10^{3}	8.91×10^{1}	3.55×10^{2}
		BG-11	Total	4.33±0.60×10^{4}	8.70±0.10×10^{4}	9.83±4.00×10^{5}	1.29±1.12×10^{5}	3.43±0.30×10^{5}	5.15±0.00×10^{5}	7.75±0.00×10^{4}	1.95±0.06×10^{5}
			AOP	1.13±0.35×10^{2}	3.97±0.45×10^{2}	1.63±0.05×10^{3}	1.83±0.25×10^{3}	1.40±0.25×10^{2}	2.05±0.00×10^{3}	1.90±1.50×10^{2}	1.80±0.30×10^{3}
		PNSm	Total	1.13±0.20×10^{2}	8.33±4.50×10^{1}	2.43±0.40×10^{2}	1.40±0.16×10^{3}	1.90±0.55×10^{2}	1.33±0.02×10^{3}	1.33±0.00×10^{1}	1.90±0.20×10^{2}
			AnAnP	0	0	0	0	0	0	0	0
	**Summer**	RO	Total	1.15±0.07×10^{4}	1.36±0.24×10^{4}	1.13±0.19×10^{4}	5.53±1.00×10^{4}	2.63±0.35×10^{4}	9.33±1.25×10^{5}	1.36±0.11×10^{4}	3.93±0.10×10^{4}
			Pigmented	8.33±3.50×10^{2}	3.75±1.35×10^{3}	2.33±0.35×10^{3}	2.17±0.55×10^{4}	1.17±0.25×10^{4}	4.10±0.65×10^{5}	4.93±0.35×10^{3}	1.77±0.05×10^{4}
			AAP	0	0	1.40×10^{3}	3.61×10^{3}	1.89×10^{3}	8.33×10^{4}	1.50×10^{3}	0
		OM	Total	1.45±0.00×10^{4}	9.10±1.30×10^{4}	3.34±0.15×10^{4}	1.10±0.05×10^{5}	4.73±0.75×10^{4}	2.45±0.06×10^{6}	2.36±0.12×10^{4}	1.10±0.02×10^{5}
			Pigmented	1.30±0.00×10^{3}	3.10±0.30×10^{4}	6.43±0.20×10^{3}	3.43±0.55×10^{4}	1.33±0.35×10^{4}	9.77±0.60×10^{5}	7.27±0.20×10^{3}	2.63±0.65×10^{4}
			AAP	0	5.64×10^{3}	1.77×10^{3}	0	3.46×10^{3}	1.87×10^{5}	3.85×10^{3}	0
		RO_T	Total	9.67±8.50×10^{1}	3.53±0.85×10^{2}	3.03±1.40×10^{2}	9.67±6.50×10^{1}	1.50±0.50×10^{2}	2.07±1.00×10^{3}	4.17±0.45×10^{2}	9.50±6.10×10^{2}
			Pigmented	9.67±8.50×10^{1}	2.87±0.65×10^{2}	2.13±1.90×10^{2}	5.00±2.50×10^{1}	1.50±0.50×10^{2}	2.00±0.90×10^{3}	3.83±0.25×10^{2}	7.63±5.20×10^{2}
			AAP	0	0	1.90×10^{2}	0	2.31×10^{1}	5.38×10^{2}	1.92×10^{2}	0
		Potato	Total	7.33±0.70×10^{3}	1.28±0.10×10^{3}	8.30±1.75×10^{3}	1.73±0.08×10^{4}	8.17±1.45×10^{3}	4.50±1.15×10^{5}	6.10±1.30×10^{3}	8.67±1.40×10^{3}
			Pigmented	7.00±2.00×10^{2}	3.20±0.50×10^{2}	2.83±0.85×10^{3}	7.07±0.25×10^{3}	3.77±0.25×10^{3}	3.43±0.35×10^{5}	3.50±0.90×10^{3}	4.47±1.30×10^{3}
			AAP	0	0	5.15×10^{2}	0	9.83×10^{2}	4.64×10^{4}	7.50×10^{2}	0
		BG-11	Total	1.23±0.07×10^{4}	1.89±0.25×10^{4}	1.33±0.50×10^{4}	1.00±0.00×10^{4}	1.17±0.40×10^{5}	1.00±0.50×10^{7}	8.67±1.00×10^{3}	1.00±1.00×10^{7}
			AOP	0	0	1.57±0.20×10^{2}	1.27±0.05×10^{3}	5.07±0.85×10^{2}	1.43±0.20×10^{4}	2.35±0.00×10^{2}	1.27±1.50×10^{3}
		PNSm	Total	7.10±0.20×10^{3}	1.57±0.09×10^{3}	2.54±0.27×10^{3}	1.22±1.70×10^{3}	2.40±0.16×10^{3}	3.93±0.60×10^{4}	9.10±0.15×10^{2}	1.06±1.20×10^{3}
			AnAnP	0	0	1.80±0.50×10^{2}	0	0	0	0	0

Table S4. Continued (3/7).

				1		2		3		4	
Year	**Season**	Medium		liquid	sediment	liquid	sediment	liquid	sediment	liquid	sediment
2018	**Fall**	RO	Total	$1.86\pm0.98\times10^{4}$	$1.92\pm0.04\times10^{4}$	$6.27\pm0.15\times10^{4}$	$3.60\pm0.55\times10^{4}$	$1.25\pm0.01\times10^{4}$	$5.70\pm0.20\times10^{3}$	$1.89\pm0.23\times10^{4}$	$1.38\pm0.26\times10^{4}$
			Pigmented	$1.63\pm0.50\times10^{3}$	$3.60\pm0.05\times10^{3}$	$1.43\pm0.00\times10^{4}$	$1.70\pm0.50\times10^{4}$	$4.70\pm0.15\times10^{3}$	$2.60\pm0.20\times10^{3}$	$7.63\pm0.05\times10^{3}$	$6.27\pm0.95\times10^{3}$
			AAP	6.53×10^{1}	3.72×10^{2}	2.61×10^{3}	4.47×10^{2}	2.61×10^{2}	2.74×10^{2}	3.55×10^{2}	4.18×10^{2}
		OM	Total	$3.87\pm0.15\times10^{4}$	$7.47\pm0.10\times10^{4}$	$1.17\pm0.04\times10^{4}$	$7.90\pm0.10\times10^{4}$	$4.57\pm0.65\times10^{4}$	$1.41\pm0.01\times10^{4}$	$3.00\pm0.13\times10^{4}$	$3.87\pm0.15\times10^{4}$
			Pigmented	$3.67\pm0.00\times10^{3}$	$1.13\pm0.50\times10^{4}$	$4.83\pm0.60\times10^{3}$	$2.17\pm0.10\times10^{4}$	$1.47\pm0.35\times10^{4}$	$3.30\pm0.25\times10^{3}$	$8.37\pm0.35\times10^{3}$	$1.10\pm0.10\times10^{4}$
			AAP	0	0	0	1.11×10^{3}	1.83×10^{3}	0	1.01×10^{3}	7.86×10^{2}
		RO_T	Total	$8.67\pm0.50\times10^{1}$	$5.70\pm0.65\times10^{2}$	$5.80\pm0.90\times10^{2}$	$1.66\pm0.12\times10^{3}$	$4.47\pm0.55\times10^{2}$	$1.13\pm0.05\times10^{2}$	$9.67\pm1.50\times10^{1}$	$7.77\pm0.70\times10^{2}$
			Pigmented	$8.67\pm0.50\times10^{1}$	$5.60\pm0.60\times10^{2}$	$5.80\pm0.90\times10^{2}$	$1.66\pm0.12\times10^{3}$	$4.47\pm0.55\times10^{2}$	$1.13\pm0.05\times10^{2}$	$8.67\pm0.00\times10^{1}$	$7.77\pm0.70\times10^{2}$
			AAP	1.73×10^{1}	0	2.42×10^{2}	5.19×10^{2}	5.58×10^{1}	0	6.67×10^{0}	2.59×10^{2}
		Potato	Total	$4.77\pm0.15\times10^{3}$	$9.70\pm0.25\times10^{2}$	$1.12\pm0.06\times10^{3}$	$4.63\pm0.05\times10^{3}$	$9.37\pm2.05\times10^{3}$	$2.73\pm0.35\times10^{3}$	$9.63\pm0.70\times10^{3}$	$9.70\pm0.40\times10^{3}$
			Pigmented	$2.43\pm0.40\times10^{3}$	$2.10\pm0.25\times10^{2}$	$4.57\pm0.50\times10^{2}$	$2.13\pm0.30\times10^{3}$	$4.23\pm0.60\times10^{3}$	$1.33\pm0.50\times10^{3}$	$5.93\pm0.70\times10^{3}$	6.30 ± 0.0510^{3}
			AAP	1.43×10^{2}	0	8.30×10^{1}	1.94×10^{2}	0	0	0	0
		BG-11	Total	$2.39\pm0.18\times10^{4}$	$5.57\pm1.95\times10^{4}$	$1.70\pm0.11\times10^{4}$	$3.30\pm0.61\times10^{4}$	$2.10\pm0.50\times10^{4}$	$4.03\pm0.45\times10^{5}$	$6.60\pm2.70\times10^{3}$	$2.26\pm0.35\times10^{6}$
			AOP	$3.30\pm0.00\times10^{0}$	$2.67\pm2.60\times10^{2}$	$8.33\pm2.00\times10^{1}$	$2.13\pm0.45\times10^{2}$	$2.35\pm0.00\times10^{2}$	$6.57\pm4.70\times10^{3}$	$1.23\pm0.35\times10^{2}$	$2.57\pm0.85\times10^{4}$
		PNSm	Total	$8.00\pm0.00\times10^{1}$	$1.27\pm0.20\times10^{2}$	$1.10\pm0.05\times10^{2}$	$9.00\pm0.50\times10^{1}$	$1.53\pm0.05\times10^{2}$	$2.40\pm0.30\times10^{2}$	$7.33\pm0.50\times10^{1}$	$7.40\pm0.10\times10^{2}$
			AnAnP	$6.67\pm0.00\times10^{0}$	$3.33\pm1.00\times10^{1}$	0	0	0	0	0	0
2019	**Spring**	RO	Total	$2{,}01\pm0.52\times10^{3}$	$1.23\pm0.26\times10^{4}$	$3.13\pm0.20\times10^{3}$	$5.05\pm0.00\times10^{4}$	$1.13\pm0.03\times10^{4}$	$8.53\pm0.15\times10^{3}$	$1.36\pm0.15\times10^{4}$	$4.40\pm0.45\times10^{5}$
			Pigmented	$3.80\pm1.20\times10^{2}$	$5.07\pm3.45\times10^{3}$	$1.33\pm0.15\times10^{3}$	$6.75\pm0.00\times10^{3}$	$4.13\pm0.10\times10^{3}$	$4.30\pm0.95\times10^{3}$	$4.37\pm0.05\times10^{3}$	$2.83\pm0.00\times10^{5}$
			AAP	1.22×10^{2}	1.97×10^{3}	3.01×10^{2}	3.21×10^{3}	1.22×10^{3}	2.04×10^{3}	1.28×10^{3}	1.77×10^{5}
		OM	Total	$1.87\pm0.43\times10^{4}$	$4.53\pm0.90\times10^{4}$	$4.03\pm0.45\times10^{4}$	$4.77\pm0.95\times10^{5}$	$8.90\pm2.65\times10^{3}$	$3.41\pm0.16\times10^{5}$	$4.97\pm0.00\times10^{4}$	$7.27\pm1.15\times10^{5}$
			Pigmented	$2.93\pm0.80\times10^{3}$	$5.33\pm0.50\times10^{3}$	$4.67\pm1.00\times10^{3}$	$6.67\pm1.50\times10^{4}$	$3.03\pm0.60\times10^{3}$	$5.03\pm0.15\times10^{4}$	$5.67\pm0.50\times10^{3}$	$1.90\pm0.20\times10^{5}$
			AAP	6.67×10^{2}	1.11×10^{3}	2.03×10^{3}	2.42×10^{4}	5.59×10^{2}	1.53×10^{4}	1.47×10^{3}	9.07×10^{4}
		RO_T	Total	$3.97\pm1.85\times10^{2}$	$5.00\pm1.80\times10^{2}$	$6.67\pm0.70\times10^{2}$	$3.05\pm0.03\times10^{3}$	$2.25\pm0.00\times10^{2}$	$3.23\pm0.67\times10^{3}$	$1.43\pm0.07\times10^{2}$	$2.03\pm0.26\times10^{3}$
			Pigmented	$3.63\pm1.55\times10^{2}$	$5.00\pm1.80\times10^{2}$	$5.23\pm0.45\times10^{2}$	$2.94\pm0.19\times10^{3}$	$1.15\pm0.00\times10^{2}$	$1.91\pm0.88\times10^{3}$	$1.27\pm0.45\times10^{2}$	$1.02\pm0.77\times10^{3}$
			AAP	3.63×10^{2}	3.00×10^{2}	3.49×10^{2}	1.07×10^{3}	2.30×10^{1}	4.78×10^{2}	0	6.14×10^{2}
		Potato	Total	$3.52\pm0.07\times10^{3}$	$2.22\pm0.01\times10^{3}$	$9.10\pm0.00\times10^{3}$	$7.43\pm0.85\times10^{4}$	$8.27\pm1.60\times10^{3}$	$2.37\pm0.01\times10^{4}$	$3.53\pm0.50\times10^{3}$	$4.50\pm0.45\times10^{4}$
			Pigmented	$5.67\pm1.10\times10^{2}$	$4.50\pm0.05\times10^{2}$	$3.35\pm0.00\times10^{3}$	$2.70\pm0.35\times10^{4}$	$3.80\pm1.05\times10^{3}$	$1.30\pm0.02\times10^{4}$	$2.53\pm0.15\times10^{3}$	$2.77\pm0.00\times10^{4}$
			AAP	2.48×10^{2}	1.31×10^{2}	1.73×10^{3}	1.35×10^{4}	2.03×10^{3}	1.13×10^{4}	1.86×10^{3}	2.40×10^{4}
		R2A	Total	N/A	$3.25\pm0.61\times10^{4}$	$5.53\pm0.60\times10^{4}$	$6.30\pm3.00\times10^{4}$	$5.90\pm0.05\times10^{4}$	$5.13\pm0.05\times10^{5}$	$2.09\pm0.22\times10^{4}$	$2.22\pm0.19\times10^{5}$
			Pigmented	N/A	$7.47\pm1.65\times10^{3}$	$9.67\pm0.45\times10^{3}$	$2.80\pm1.80\times10^{4}$	$1.70\pm0.35\times10^{4}$	$2.00\pm0.45\times10^{5}$	$7.60\pm1.05\times10^{3}$	$8.17\pm1.15\times10^{4}$
			AAP	N/A	1.74×10^{3}	2.94×10^{3}	1.01×10^{4}	1.79×10^{3}	1.36×10^{5}	5.43×10^{3}	4.67×10^{4}
		BG-11	Total	$2.14\pm0.74\times10^{4}$	$4.45\pm1.25\times10^{4}$	$1.07\pm0.02\times10^{5}$	$2.30\pm0.06\times10^{5}$	$4.57\pm0.00\times10^{4}$	$1.00\pm0.10\times10^{6}$	$5.33\pm0.65\times10^{4}$	$4.57\pm0.20\times10^{5}$
			AOP	$1.30\pm0.05\times10^{2}$	$5.33\pm0.50\times10^{1}$	0	$3.50\pm0.30\times10^{4}$	0	$4.43\pm2.05\times10^{4}$	$2.33\pm0.00\times10^{1}$	$4.87\pm0.40\times10^{2}$
		PNSm	Total	$5.77\pm2.35\times10^{2}$	$2.67\pm0.00\times10^{1}$	$3.83\pm0.10\times10^{2}$	$1.32\pm0.04\times10^{4}$	$1.05\pm0.05\times10^{3}$	$3.43\pm0.10\times10^{3}$	$5.67\pm1.50\times10^{1}$	$5.00\pm5.10\times10^{2}$
			AnAnP	$4.33\pm2.50\times10^{1}$	0	0	0	$1.33\pm2.00\times10^{1}$	$3.33\pm5.00\times10^{1}$	0	$1.00\pm1.50\times10^{1}$
	**Summer**	RO	Total	$5.63\pm1.50\times10^{3}$	$5.77\pm0.75\times10^{4}$	$7.50\pm3.10\times10^{4}$	$4.40\pm0.75\times10^{6}$	$6.80\pm0.30\times10^{4}$	$1.05\pm0.07\times10^{5}$	$9.63\pm0.50\times10^{3}$	$2.48\pm0.26\times10^{5}$
			Pigmented	$3.07\pm0.60\times10^{3}$	0	$2.40\pm0.25\times10^{4}$	$2.03\pm0.30\times10^{5}$	$2.93\pm0.25\times10^{4}$	$5.07\pm0.30\times10^{4}$	$3.33\pm0.40\times10^{3}$	$8.20\pm0.95\times10^{4}$
			AAP	6.13×10^{2}	0	5.19×10^{3}	4.15×10^{4}	6.29×10^{3}	1.27×10^{4}	4.76×10^{2}	2.39×10^{4}
		OM	Total	$4.57\pm0.20\times10^{4}$	N/A	$1.43\pm0.16\times10^{5}$	$6.40\pm2.90\times10^{6}$	$5.73\pm1.65\times10^{4}$	$3.37\pm0.15\times10^{5}$	$3.36\pm0.11\times10^{4}$	$1.11\pm0.13\times10^{6}$
			Pigmented	$5.00\pm1.00\times10^{3}$	N/A	$2.73\pm1.20\times10^{4}$	$1.97\pm0.45\times10^{5}$	$2.03\pm0.30\times10^{4}$	$1.50\pm0.45\times10^{5}$	$2.47\pm0.35\times10^{3}$	$1.87\pm0.20\times10^{5}$
			AAP	0	0	3.28×10^{3}	4.57×10^{4}	0	1.10×10^{4}	5.23×10^{2}	1.78×10^{4}
		RO_T	Total	$7.17\pm2.80\times10^{2}$	$4.17\pm1.25\times10^{3}$	$1.70\pm0.03\times10^{3}$	$5.07\pm1.20\times10^{4}$	$1.22\pm0.20\times10^{3}$	$3.92\pm0.29\times10^{3}$	$1.77\pm0.50\times10^{2}$	$9.83\pm0.45\times10^{3}$
			Pigmented	$7.13\pm2.75\times10^{2}$	$2.87\pm1.20\times10^{3}$	$1.51\pm0.01\times10^{3}$	$2.57\pm0.10\times10^{3}$	$7.20\pm2.20\times10^{2}$	$3.42\pm0.32\times10^{3}$	$1.77\pm0.50\times10^{2}$	$5.93\pm0.95\times10^{3}$
			AAP	1.43×10^{2}	0	1.08×10^{3}	1.14×10^{4}	0	2.63×10^{3}	1.33×10^{2}	4.94×10^{3}
		Potato	Total	$1.43\pm0.15\times10^{4}$	$9.47\pm0.85\times10^{3}$	$5.60\pm0.80\times10^{4}$	$1.05\pm0.44\times10^{5}$	$6.00\pm3.80\times10^{3}$	$4.57\pm0.35\times10^{4}$	$1.54\pm0.13\times10^{3}$	$3.44\pm0.99\times10^{4}$
			Pigmented	$2.67\pm0.40\times10^{3}$	$3.37\pm0.40\times10^{3}$	$2.27\pm0.10\times10^{4}$	$4.23\pm1.15\times10^{5}$	$2.83\pm0.65\times10^{3}$	$2.70\pm0.35\times10^{4}$	$4.93\pm0.60\times10^{2}$	$9.37\pm3.55\times10^{3}$
			AAP	2.67×10^{2}	0	2.06×10^{3}	7.70×10^{4}	5.83×10^{2}	4.70×10^{3}	1.37×10^{2}	2.04×10^{3}
		R2A	Total	$1.56\pm0.03\times10^{4}$	$4.70\pm0.40\times10^{4}$	$1.31\pm0.25\times10^{5}$	$9.93\pm0.30\times10^{5}$	$1.23\pm0.05\times10^{5}$	$1.00\pm0.18\times10^{5}$	$3.35\pm0.42\times10^{4}$	$8.30\pm1.30\times10^{5}$
				Pigmented	$1.80\pm0.25\times10^{3}$	$6.00\pm0.50\times10^{2}$	$3.50\pm0.30\times10^{4}$	$4.53\pm0.55\times10^{5}$	$4.77\pm1.55\times10^{4}$	$5.30\pm1.70\times10^{4}$	$1.02\pm0.13\times10^{4}$
			AAP	0	2.86×10^{2}	0	9.71×10^{4}	2.38×10^{3}	4.82×10^{3}	1.20×10^{3}	2.50×10^{4}
		BG-11	Total	$1.43\pm0.07\times10^{4}$	$3.92\pm0.26\times10^{4}$	$5.17\pm1.00\times10^{5}$	$6.87\pm0.40\times10^{6}$	$1.21\pm0.07\times10^{5}$	$5.23\pm0.10\times10^{5}$	$8.60\pm2.85\times10^{4}$	$4.97\pm0.95\times10^{6}$
			AOP	$4.44\pm1.33\times10^{3}$	$1.37\pm0.03\times10^{3}$	$8.57\pm0.00\times10^{3}$	$1.33\pm0.05\times10^{4}$	$7.20\pm0.10\times10^{2}$	$2.33\pm1.20\times10^{2}$	$8.47\pm6.00\times10^{2}$	$1.70\pm0.14\times10^{4}$
		PNSm	Total	$1.33\pm0.50\times10^{1}$	$2.67\pm0.50\times10^{1}$	$6.83\pm0.55\times10^{2}$	$4.23\pm0.65\times10^{2}$	$3.33\pm0.75\times10^{1}$	$1.99\pm0.07\times10^{3}$	$9.47\pm0.10\times10^{3}$	$4.64\pm0.69\times10^{3}$
			AnAnP	0	0	$1.50\pm0.10\times10^{2}$	0	0	0	$1.67\pm0.00\times10^{1}$	0
	**Fall**	RO	Total	$1.13\pm0.08\times10^{4}$	$5.07\pm0.40\times10^{4}$	$4.47\pm0.05\times10^{3}$	$8.17\pm0.15\times10^{3}$	$1.76\pm0.84\times10^{4}$	$6.80\pm0.55\times10^{4}$	$9.43\pm1.95\times10^{3}$	$5.47\pm0.15\times10^{5}$
			Pigmented	$2.97\pm0.60\times10^{3}$	$2.03\pm0.30\times10^{4}$	$2.77\pm0.55\times10^{3}$	$2.70\pm0.05\times10^{3}$	$3.87\pm1.70\times10^{3}$	$3.20\pm0.60\times10^{4}$	$3.03\pm1.60\times10^{3}$	$2.80\pm0.00\times10^{5}$
			AAP	2.97×10^{2}	1.36×10^{3}	0	5.79×10^{2}	8.79×10^{2}	1.01×10^{4}	4.33×10^{2}	6.67×10^{3}
		OM	Total	$1.71\pm0.04\times10^{4}$	$2.46\pm0.19\times10^{5}$	$6.03\pm0.15\times10^{4}$	$6.83\pm2.10\times10^{4}$	$1.57\pm0.19\times10^{4}$	$1.36\pm0.09\times10^{5}$	$4.43\pm0.25\times10^{4}$	$4.70\pm0.15\times10^{6}$
			Pigmented	$4.25\pm1.85\times10^{3}$	$2.63\pm0.35\times10^{4}$	$5.33\pm0.50\times10^{3}$	$1.53\pm0.30\times10^{4}$	$2.13\pm0.35\times10^{3}$	$2.90\pm0.60\times10^{4}$	$9.33\pm2.50\times10^{3}$	$147\pm0.05\times10^{6}$
			AAP	7.08×10^{2}	6.58×10^{3}	6.27×10^{2}	2.19×10^{3}	5.33×10^{2}	2.90×10^{3}	1.65×10^{3}	4.73×10^{4}
		RO_T	Total	$1.08\pm0.02\times10^{3}$	$8.30\pm1.45\times10^{3}$	$1.56\pm0.10\times10^{3}$	$6.50\pm0.65\times10^{2}$	$6.60\pm0.25\times10^{2}$	$9.63\pm2.00\times10^{2}$	$7.25\pm0.00\times10^{2}$	$2.60\pm0.40\times10^{4}$
			Pigmented	$8.03\pm2.45\times10^{2}$	$8.10\pm1.30\times10^{3}$	$1.24\pm0.10\times10^{3}$	$4.83\pm0.15\times10^{2}$	$3.30\pm0.20\times10^{2}$	$7.97\pm1.85\times10^{2}$	$5.95\pm0.00\times10^{2}$	$2.60\pm0.40\times10^{4}$
			AAP	1.15×10^{2}	0	4.97×10^{2}	8.53×10^{1}	1.10×10^{2}	5.98×10^{2}	8.93×10^{1}	1.53×10^{4}
		Potato	Total	$1.13\pm0.00\times10^{4}$	$2.82\pm0.00\times10^{4}$	$8.80\pm0.00\times10^{3}$	$3.33\pm0.05\times10^{3}$	$4.80\pm3.40\times10^{3}$	$6.20\pm0.20\times10^{4}$	$7.70\pm0.85\times10^{3}$	$2.36\pm0.12\times10^{6}$
			Pigmented	$3.60\pm0.00\times10^{3}$	$3.97\pm0.35\times10^{3}$	$2.55\pm0.00\times10^{3}$	$1.43\pm0.05\times10^{3}$	$1.53\pm1.20\times10^{3}$	$4.17\pm0.60\times10^{4}$	$4.10\pm0.60\times10^{3}$	$1.57\pm0.75\times10^{6}$
			AAP	4.80×10^{2}	5.29×10^{2}	2.32×10^{2}	0	6.81×10^{2}	1.39×10^{4}	1.23×10^{3}	0
		R2A	Total	$1.53\pm0.55\times10^{5}$	$7.20\pm2.05\times10^{4}$	$1.89\pm0.38\times10^{4}$	$1.43\pm0.05\times10^{4}$	$1.24\pm0.15\times10^{4}$	$1.15\pm0.03\times10^{5}$	$1.79\pm0.19\times10^{4}$	$6.67\pm0.65\times10^{5}$
			Pigmented	$1.57\pm0.20\times10^{4}$	$1.87\pm0.20\times10^{4}$	$3.43\pm2.20\times10^{3}$	$1.83\pm0.05\times10^{4}$	$3.60\pm0.85\times10^{3}$	$5.20\pm0.10\times10^{4}$	$5.13\pm0.85\times10^{3}$	$2.67\pm0.70\times10^{5}$
			AAP	1.12×10^{3}	1.56×10^{3}	3.81×10^{2}	1.02×10^{3}	0	1.20×10^{4}	5.70×10^{2}	9.20×10^{3}
		BG-11	Total	$6.50\pm0.35\times10^{4}$	$2.07\pm0.10\times10^{5}$	$1.22\pm0.17\times10^{5}$	$1.54\pm0.25\times10^{5}$	$5.83\pm0.05\times10^{4}$	$3.13\pm0.20\times10^{5}$	$7.03\pm1.75\times10^{4}$	$8.27\pm1.65\times10^{6}$
			AOP	$1.60\pm0.01\times10^{3}$	$2.10\pm0.00\times10^{2}$	0	$4.33\pm1.00\times10^{1}$	$3.33\pm0.00\times10^{0}$	$5.33\pm1.50\times10^{1}$	$4.00\pm2.00\times10^{1}$	$7.80\pm0.15\times10^{2}$
		PNSm	Total	$5.50\pm2.55\times10^{3}$	$1.17\pm0.00\times10^{3}$	$5.20\pm0.10\times10^{2}$	$2.80\pm0.00\times10^{2}$	$2.80\pm0.40\times10^{2}$	$2.93\pm0.15\times10^{2}$	$1.80\pm0.05\times10^{2}$	$3.47\pm0.25\times10^{3}$
			AnAnP	$0.66\pm1.00\times10^{3}$	0	0	0	0	0	0	0

Table S4. Continued (4/7).

Year	Season	Medium		5		6	7	8	9	10
				liquid	sediment	liquid	liquid	liquid	liquid	liquid
2016	**Spring**	RO	Total	$1.53\pm0.10\times10^{3}$	$7.90\pm0.00\times10^{4}$	$1.01\pm0.60\times10^{4}$	$1.02\pm0.17\times10^{4}$	$2.18\pm0.81\times10^{4}$	$7.50\pm1.20\times10^{3}$	$4.45\pm0.35\times10^{3}$
			Pigmented	$3.00\pm0.20\times10^{2}$	$1.40\pm1.40\times10^{4}$	$4.95\pm1.35\times10^{3}$	$4.20\pm2.00\times10^{3}$	$1.24\pm0.01\times10^{4}$	$6.65\pm1.55\times10^{3}$	$3.70\pm0.00\times10^{3}$
			AAP	8.68×10^{1}	8.33×10^{3}	7.40×10^{2}	2.55×10^{2}	3.92×10^{2}	1.05×10^{3}	2.87×10^{2}
		OM	Total	$1.86\pm1.07\times10^{4}$	$2.77\pm0.39\times10^{5}$	$2.38\pm0.31\times10^{4}$	$3.35\pm0.35\times10^{4}$	$8.90\pm0.20\times10^{3}$	$1.38\pm0.14\times10^{4}$	$3.909\pm0.70\times10^{3}$
			Pigmented	$5.35\pm0.35\times10^{2}$	$1.95\pm1.35\times10^{4}$	$5.50\pm0.30\times10^{3}$	$1.45\pm0.05\times10^{4}$	$3.40\pm1.60\times10^{3}$	$6.25\pm0.85\times10^{3}$	$1.50\pm0.00\times10^{3}$
			AAP	1.34×10^{1}	6.91×10^{3}	1.59×10^{2}	3.15×10^{2}	4.36×10^{1}	8.12×10^{1}	2.03×10^{1}
		RO_T	Total	$1.70\pm0.00\times10^{2}$	$6.50\pm0.00\times10^{2}$	$1.10\pm0.10\times10^{2}$	0	$1.00\pm0.00\times10^{1}$	$3.80\pm1.20\times10^{2}$	$2.00\pm0.00\times10^{1}$
			Pigmented	$1.50\pm0.00\times10^{2}$	$5.90\pm0.00\times10^{2}$	$1.10\pm0.10\times10^{2}$	0	$1.00\pm0.00\times10^{1}$	$3.80\pm1.20\times10^{2}$	$2.00\pm0.00\times10^{1}$
			AAP	0	4.29×10^{2}	3.47×10^{1}	0	0	7.60×10^{1}	0
		Potato	Total	$7.80\pm0.00\times10^{2}$	$3.70\pm0.00\times10^{4}$	$1.20\pm0.20\times10^{3}$	$2.95\pm0.65\times10^{3}$	$2.50\pm0.60\times10^{2}$	$1.80\pm0.20\times10^{2}$	$3.65\pm0.55\times10^{2}$
			Pigmented	$2.00\pm0.00\times10^{2}$	$5.00\pm0.00\times10^{3}$	$7.75\pm5.75\times10^{2}$	$1.00\pm1.00\times10^{2}$	$8.00\pm3.00\times10^{1}$	$2.50\pm0.50\times10^{1}$	$1.00\pm0.10\times10^{2}$
			AAP	2.50×10^{1}	9.09×10^{2}	4.13×10^{2}	1.43×10^{1}	4.71×10^{0}	8.33×10^{0}	0
		BG-11	Total	$2.14\pm0.00\times10^{5}$	$1.00\pm0.00\times10^{6}$	$2.31\pm0.84\times10^{4}$	$1.86\pm0.07\times10^{4}$	$1.42\pm1.07\times10^{4}$	$7.65\pm1.65\times10^{3}$	N/A
			AOP	$2.40\pm0.00\times10^{3}$	$1.00\pm0.00\times10^{2}$	$1.50\pm0.50\times10^{1}$	$5.00\pm5.00\times10^{0}$	0	$6.50\pm2.50\times10^{1}$	N/A
		PNSm	Total	$1.20\pm0.00\times10^{2}$	$6.90\pm0.00\times10^{2}$	$1.05\pm0.25\times10^{2}$	$1.50\pm0.10\times10^{2}$	$6.00\pm3.00\times10^{1}$	$2.50\pm0.50\times10^{1}$	0
			AnAnP	0	$1.00\pm0.00\times10^{1}$	0	0	0	0	0
	**Summer**	RO	Total	$6.75\pm1.25\times10^{3}$	$2.01\pm0.24\times10^{3}$	$5.40\pm0.20\times10^{2}$	$3.35\pm0.15\times10^{3}$	$1.34\pm0.15\times10^{3}$	$3.05\pm0.05\times10^{3}$	$1.53\pm0.20\times10^{3}$
			Pigmented	$2.10\pm0.20\times10^{3}$	$4.20\pm0.40\times10^{2}$	$8.00\pm5.00\times10^{1}$	$2.90\pm0.20\times10^{3}$	$3.70\pm0.30\times10^{2}$	$2.60\pm0.30\times10^{3}$	$1.32\pm0.22\times10^{3}$
			AAP	4.20×10^{2}	9.16×10^{1}	4.21×10^{0}	3.45×10^{2}	3.36×10^{1}	3.59×10^{2}	8.22×10^{1}
		OM	Total	$1.89\pm0.27\times10^{4}$	$1.28\pm0.24\times10^{4}$	$1.85\pm0.25\times10^{3}$	$1.88\pm0.04\times10^{3}$	$2.73\pm0.04\times10^{3}$	$4.90\pm0.20\times10^{3}$	$4.90\pm1.10\times10^{3}$
			Pigmented	$2.35\pm0.35\times10^{3}$	$1.35\pm0.85\times10^{3}$	$4.00\pm1.00\times10^{2}$	$1.19\pm0.04\times10^{3}$	$6.30\pm0.70\times10^{2}$	$2.75\pm0.55\times10^{3}$	$1.45\pm0.55\times10^{3}$
			AAP	2.40×10^{2}	1.78×10^{2}	3.33×10^{1}	3.61×10^{1}	1.75×10^{1}	8.87×10^{1}	9.35×10^{1}
		RO_T	Total	$7.40\pm0.00\times10^{2}$	$3.00\pm0.00\times10^{2}$	$1.10\pm4.00\times10^{2}$	$4.05\pm0.65\times10^{2}$	$3.20\pm0.00\times10^{2}$	$9.00\pm0.00\times10^{1}$	$3.40\pm0.00\times10^{2}$
			Pigmented	$7.00\pm0.00\times10^{2}$	$2.90\pm0.00\times10^{2}$	$1.10\pm0.40\times10^{2}$	$4.05\pm0.65\times10^{2}$	$1.00\pm0.00\times10^{2}$	$9.00\pm0.00\times10^{1}$	$3.40\pm0.00\times10^{2}$
			AAP	0	0	0	9.42×10^{0}	0	0	0
		Potato	Total	$8.88\pm0.00\times10^{2}$	$4.50\pm0.00\times10^{2}$	$2.20\pm0.00\times10^{3}$	$4.00\pm0.00\times10^{3}$	$8.00\pm0.00\times10^{2}$	$2.00\pm0.00\times10^{3}$	$5.70\pm0.00\times10^{2}$
			Pigmented	$3.20\pm0.00\times10^{2}$	$6.00\pm0.00\times10^{1}$	$1.80\pm0.00\times10^{3}$	$3.70\pm0.00\times10^{3}$	$1.90\pm0.00\times10^{2}$	$7.70\pm0.00\times10^{3}$	$1.40\pm0.00\times10^{2}$
			AAP	0	0	0	0	0	0	0
		BG-11	Total	$2.10\pm0.00\times10^{5}$	$9.00\pm0.00\times10^{5}$	$7.00\pm0.00\times10^{3}$	$6.70\pm0.00\times10^{3}$	$4.00\pm0.00\times10^{3}$	$4.70\pm0.00\times10^{3}$	$3.40\pm0.00\times10^{3}$
			AOP	$4.40\pm0.00\times10^{2}$	0	$1.20\pm0.00\times10^{2}$	$1.00\pm0.00\times10^{2}$	0	0	$1.00\pm0.00\times10^{2}$
		PNSm	Total	$4.80\pm0.00\times10^{2}$	$1.60\pm0.00\times10^{2}$	$5.00\pm5.00\times10^{0}$	$3.00\pm2.00\times10^{1}$	$2.40\pm0.00\times10^{2}$	$1.90\pm0.00\times10^{2}$	$9.00\pm0.00\times10^{1}$
			AnAnP	0	$1.00\pm0.00\times10^{1}$	0	0	0	0	0
	**Fall**	RO	Total	$1.41\pm0.60\times10^{4}$	$1.07\pm0.11\times10^{5}$	$6.15\pm0.65\times10^{2}$	$8.35\pm1.35\times10^{3}$	$1.20\pm0.40\times10^{2}$	$7.45\pm1.65\times10^{2}$	$4.35\pm0.85\times10^{2}$
			Pigmented	$4.60\pm4.10\times10^{3}$	$1.10\pm0.40\times10^{5}$	$4.65\pm0.85\times10^{2}$	$7.40\pm1.50\times10^{3}$	$7.50\pm1.50\times10^{1}$	$3.15\pm0.85\times10^{2}$	$2.60\pm0.50\times10^{2}$
			AAP	8.00×10^{2}	7.33×10^{3}	0	3.08×10^{2}	0	0	0
		OM	Total	$8.25\pm1.55\times10^{4}$	$1.15\pm0.45\times10^{6}$	$2.57\pm0.69\times10^{3}$	$9.80\pm1.60\times10^{3}$	$1.61\pm0.02\times10^{3}$	$5.70\pm0.00\times10^{3}$	$1.90\pm0.62\times10^{3}$
			Pigmented	$1.35\pm0.15\times10^{4}$	$1.50\pm0.50\times10^{5}$	$4.40\pm0.60\times10^{2}$	$8.05\pm1.15\times10^{3}$	$4.10\pm1.30\times10^{2}$	$9.50\pm2.50\times10^{2}$	$3.70\pm1.30\times10^{2}$
			AAP	1.35×10^{3}	0	0	0	0	0	0
		RO_T	Total	$3.80\pm0.00\times10^{2}$	$4.00\pm0.00\times10^{4}$	0	$3.10\pm0.00\times10^{3}$	$1.10\pm0.00\times10^{2}$	$5.00\pm0.00\times10^{1}$	$1.60\pm0.00\times10^{2}$
			Pigmented	$3.80\pm0.00\times10^{2}$	$3.90\pm0.00\times10^{4}$	0	$3.10\pm0.00\times10^{3}$	$1.10\pm0.00\times10^{2}$	$4.00\pm0.00\times10^{1}$	$1.50\pm0.00\times10^{2}$
			AAP	0	0	0	0	0	0	0
		Potato	Total	$5.60\pm0.00\times10^{3}$	$6.50\pm0.00\times10^{4}$	$3.80\pm0.00\times10^{2}$	$4.30\pm0.00\times10^{3}$	$2.90\pm0.00\times10^{2}$	$2.90\pm0.00\times10^{2}$	$4.40\pm0.00\times10^{2}$
			Pigmented	$2.10\pm0.00\times10^{3}$	$5.50\pm0.00\times10^{4}$	$2.00\pm0.00\times10^{2}$	$4.30\pm0.00\times10^{3}$	$2.00\pm0.00\times10^{2}$	$2.20\pm0.00\times10^{2}$	$3.90\pm0.00\times10^{2}$
			AAP	0	0	0	0	0	0	0
		BG-11	Total	$3.50\pm0.20\times10^{4}$	$3.25\pm0.86\times10^{5}$	$8.70\pm0.00\times10^{3}$	$1.05\pm0.00\times10^{4}$	$2.40\pm0.00\times10^{2}$	$1.60\pm0.00\times10^{2}$	$1.63\pm0.00\times10^{4}$
			AOP	$8.50\pm0.50\times10^{1}$	$5.25\pm0.25\times10^{2}$	$4.00\pm0.00\times10^{1}$	$2.00\pm0.00\times10^{1}$	0	$1.00\pm0.00\times10^{1}$	$2.00\pm0.00\times10^{1}$
		PNSm	Total	$1.30\pm0.00\times10^{2}$	$1.01\pm0.00\times10^{3}$	$6.00\pm0.00\times10^{1}$	$2.00\pm0.00\times10^{1}$	$5.00\pm0.00\times10^{1}$	$9.00\pm0.00\times10^{1}$	$1.00\pm0.00\times10^{1}$
			AnAnP	0	N/A	0	0	0	0	0
2017	**Winter**	Alternative Sites				W10	**W1**	W9	**W6**	**W8**
		RO	Total	N/A	N/A	N/A	N/A	$2.00\pm0.00\times10^{1}$	N/A	N/A
			Pigmented	N/A	N/A	N/A	N/A	1.00 ± 0.5010^{1}	N/A	N/A
			AAP	N/A	N/A	N/A	N/A	0	N/A	N/A
		OM	Total	N/A	N/A	N/A	N/A	$1.44\pm0.50\times10^{1}$	N/A	N/A
			Pigmented	N/A	N/A	N/A	N/A	$3.33\pm0.00\times10^{0}$	N/A	N/A
			AAP	N/A	N/A	N/A	N/A	0	N/A	N/A
		RO_T	Total	N/A	N/A	N/A	N/A	$6.67\pm0.50\times10^{0}$	N/A	N/A
			Pigmented	N/A	N/A	N/A	N/A	$3.33\pm0.00\times10^{0}$	N/A	N/A
			AAP	N/A	N/A	N/A	N/A	0	N/A	N/A
		Potato	Total	N/A	N/A	N/A	N/A	$3.33\pm0.00\times10^{0}$	N/A	N/A
			Pigmented	N/A	N/A	N/A	N/A	0	N/A	N/A
			AAP	N/A	N/A	N/A	N/A	0	N/A	N/A
		BG-11	Total	N/A	N/A	N/A	N/A	$7.67\pm3.00\times10^{1}$	N/A	N/A
			AOP	N/A	N/A	N/A	N/A	0	N/A	N/A
		PNSm	Total	N/A	N/A	N/A	N/A	$1.33\pm0.50\times10^{1}$	N/A	N/A
			AnAnP	N/A	N/A	N/A	N/A	0	N/A	N/A

Table S4. Continued (5/7).

Year	Season	Medium		5 liquid	5 sediment	6 liquid	7 liquid	8 liquid	9 liquid	10 liquid
2017	**Spring**	RO	Total	$5.10\pm0.25\times10^{3}$	$1.23\pm0.07\times10^{4}$	$2.09\pm0.97\times10^{3}$	$1.08\pm0.26\times10^{3}$	$1.94\pm0.23\times10^{4}$	$8.30\pm0.70\times10^{3}$	$6.40\pm0.60\times10^{3}$
			Pigmented	$2.50\pm0.40\times10^{3}$	$3.80\pm0.25\times10^{3}$	$4.33\pm5.01\times10^{2}$	$9.67\pm1.00\times10^{1}$	$1.44\pm0.48\times10^{4}$	$4.50\pm0.35\times10^{3}$	$5.20\pm0.20\times10^{3}$
			AAP	1.12×10^{3}	3.68×10^{2}	0	8.79×10^{0}	1.80×10^{3}	4.29×10^{2}	8.67×10^{2}
		OM	Total	$1.23\pm0.03\times10^{4}$	$7.97\pm0.40\times10^{4}$	$3.05\pm0.11\times10^{3}$	$2.35\pm0.18\times10^{3}$	$3.35\pm0.11\times10^{4}$	$1.18\pm0.04\times10^{4}$	$9.67\pm1.55\times10^{3}$
			Pigmented	$2.67\pm0.35\times10^{3}$	$8.67\pm3.00\times10^{3}$	$1.63\pm0.42\times10^{2}$	$8.33\pm0.00\times10^{1}$	$2.37\pm0.13\times10^{4}$	$2.93\pm0.75\times10^{3}$	$5.03\pm2.10\times10^{3}$
			AAP	7.41×10^{2}	1.93×10^{3}	2.04×10^{1}	0	3.38×10^{3}	1.30×10^{3}	3.36×10^{3}
		RO$_T$	Total	$1.17\pm0.30\times10^{2}$	$5.67\pm2.50\times10^{1}$	0	0	$6.67\pm5.00\times10^{0}$	$6.67\pm0.00\times10^{0}$	0
			Pigmented	$7.00\pm0.50\times10^{1}$	$2.00\pm3.00\times10^{1}$	0	0	$3.33\pm0.00\times10^{0}$	$6.67\pm0.00\times10^{0}$	0
			AAP	0	6.67×10^{0}	0	0	0	0	0
		Potato	Total	$8.67\pm2.50\times10^{2}$	$8.33\pm2.00\times10^{3}$	$1.00\pm0.36\times10^{3}$	$2.40\pm0.10\times10^{3}$	$1.03\pm0.15\times10^{3}$	$1.10\pm0.10\times10^{3}$	$5.67\pm2.50\times10^{2}$
			Pigmented	$7.80\pm0.40\times10^{3}$	$1.14\pm0.05\times10^{5}$	$4.63\pm0.79\times10^{3}$	$3.67\pm0.50\times10^{3}$	$3.47\pm0.25\times10^{3}$	$1.80\pm0.15\times10^{3}$	$9.33\pm1.50\times10^{2}$
			AAP	1.49×10^{3}	4.97×10^{3}	1.05×10^{3}	2.44×10^{2}	2.04×10^{2}	4.29×10^{2}	5.33×10^{2}
		BG-11	Total	$2.29\pm0.09\times10^{4}$	$1.55\pm0.08\times10^{5}$	$6.00\pm0.00\times10^{3}$	$2.92\pm0.20\times10^{3}$	$1.38\pm0.65\times10^{4}$	$1.21\pm0.13\times10^{4}$	$6.83\pm0.25\times10^{3}$
			AOP	$2.30\pm1.80\times10^{2}$	$2.00\pm0.50\times10^{2}$	$2.03\pm2.74\times10^{2}$	$2.77\pm0.15\times10^{2}$	0	0	0
		PNSm	Total	$1.10\pm0.45\times10^{2}$	$1.41\pm0.54\times10^{3}$	$4.00\pm1.63\times10^{1}$	0	$9.33\pm0.50\times10^{1}$	$5.67\pm0.50\times10^{1}$	$3.37\pm0.10\times10^{2}$
			AnAnP	0	$2.70\pm0.35\times10^{2}$	0	0	0	0	0
	**Summer**	RO	Total	$1.45\pm0.00\times10^{4}$	$5.15\pm0.25\times10^{5}$	$5.37\pm0.13\times10^{3}$	$4.92\pm0.73\times10^{3}$	$7.80\pm0.95\times10^{2}$	$3.20\pm0.65\times10^{3}$	$8.03\pm0.25\times10^{2}$
			Pigmented	$4.50\pm0.00\times10^{3}$	$1.55\pm0.15\times10^{5}$	$4.50\pm0.08\times10^{3}$	$4.11\pm0.61\times10^{3}$	$1.63\pm0.05\times10^{2}$	$9.67\pm1.00\times10^{2}$	$4.40\pm0.30\times10^{2}$
			AAP	0	0	1.88×10^{2}	1.21×10^{2}	1.63×10^{1}	1.38×10^{2}	0
		OM	Total	$1.77\pm0.65\times10^{4}$	$1.40\pm0.11\times10^{6}$	$5.60\pm1.06\times10^{3}$	$5.44\pm0.46\times10^{3}$	$1.21\pm0.00\times10^{3}$	$4.47\pm0.15\times10^{3}$	$1.47\pm0.01\times10^{3}$
			Pigmented	$2.43\pm0.00\times10^{3}$	$2.33\pm0.20\times10^{5}$	$4.53\pm1.10\times10^{3}$	$4.37\pm0.29\times10^{3}$	$2.10\pm0.00\times10^{2}$	$1.10\pm0.40\times10^{3}$	$5.17\pm0.70\times10^{2}$
			AAP	8.39×10^{1}	1.46×10^{4}	1.36×10^{3}	0	2.10×10^{1}	8.80×10^{1}	0
		RO$_T$	Total	$9.33\pm2.00\times10^{1}$	$1.40\pm0.65\times10^{3}$	$1.37\pm0.24\times10^{2}$	$1.47\pm0.10\times10^{2}$	$2.67\pm0.50\times10^{1}$	$3.67\pm0.00\times10^{1}$	$4.67\pm3.00\times10^{1}$
			Pigmented	$8.33\pm0.50\times10^{1}$	$1.13\pm0.40\times10^{3}$	$1.23\pm0.13\times10^{2}$	$1.30\pm0.00\times10^{2}$	$1.67\pm1.50\times10^{1}$	$3.67\pm0.00\times10^{1}$	$4.33\pm3.00\times10^{1}$
			AAP	0	0	0	0	0	0	0
		Potato	Total	$3.90\pm0.95\times10^{3}$	$7.83\pm3.10\times10^{4}$	$6.30\pm0.78\times10^{2}$	$6.10\pm0.40\times10^{3}$	$6.57\pm0.05\times10^{2}$	$1.16\pm0.09\times10^{3}$	$5.63\pm1.05\times10^{2}$
			Pigmented	$1.97\pm0.45\times10^{3}$	$2.37\pm1.00\times10^{4}$	$5.13\pm0.74\times10^{3}$	$5.23\pm0.50\times10^{3}$	$1.07\pm0.05\times10^{2}$	$3.80\pm0.45\times10^{2}$	$3.23\pm0.25\times10^{2}$
			AAP	1.64×10^{2}	1.18×10^{3}	0	0	9.28×10^{0}	2.38×10^{1}	2.81×10^{1}
		BG-11	Total	$4.30\pm1.35\times10^{4}$	$1.83\pm0.30\times10^{6}$	$4.27\pm0.13\times10^{3}$	$7.40\pm1.00\times10^{3}$	$1.29\pm0.21\times10^{4}$	$1.39\pm0.08\times10^{3}$	$1.66\pm0.14\times10^{3}$
			AOP	$1.93\pm0.15\times10^{3}$	0	$9.33\pm2.05\times10^{1}$	$2.00\pm0.00\times10^{1}$	$8.33\pm6.00\times10^{2}$	0	0
		PNSm	Total	$9.30\pm0.15\times10^{2}$	$1.03\pm0.55\times10^{4}$	$2.67\pm0.47\times10^{1}$	$2.67\pm0.5\times10^{1}$	$1.30\pm0.45\times10^{2}$	$5.00\pm0.00\times10^{1}$	$3.00\pm1.00\times10^{1}$
			AnAnP	$3.33\pm5.00\times10^{0}$	$1.67\pm0.50\times10^{1}$	0	0	0	0	0
	**Fall**	RO	Total	$9.30\pm0.30\times10^{3}$	$2.50\pm0.14\times10^{5}$	$1.03\pm0.56\times10^{4}$	$5.57\pm0.40\times10^{3}$	$8.73\pm2.45\times10^{3}$	$1.01\pm0.22\times10^{3}$	$3.87\pm0.25\times10^{3}$
			Pigmented	$2.93\pm0.10\times10^{3}$	$5.10\pm0.55\times10^{4}$	$5.70\pm4.05\times10^{3}$	$2.83\pm0.15\times10^{3}$	$2.30\pm0.90\times10^{3}$	$1.47\pm0.05\times10^{2}$	$1.10\pm0.50\times10^{3}$
			AAP	7.04×10^{2}	5.80×10^{3}	0	0	0	0	0
		OM	Total	$1.41\pm0.14\times10^{4}$	$9.87\pm2.40\times10^{4}$	$2.59\pm0.26\times10^{4}$	$7.73\pm0.28\times10^{3}$	$5.60\pm0.10\times10^{4}$	$6.23\pm1.50\times10^{3}$	$1.28\pm0.08\times10^{4}$
			Pigmented	$2.00\pm0.70\times10^{3}$	$3.67\pm0.75\times10^{4}$	$5.87\pm0.42\times10^{3}$	$3.43\pm0.90\times10^{3}$	$1.13\pm0.45\times10^{4}$	$8.67\pm4.50\times10^{2}$	$3.70\pm1.85\times10^{3}$
			AAP	0	9.91×10^{2}	0	0	0	0	0
		RO$_T$	Total	$2.70\pm0.30\times10^{2}$	$4.17\pm0.65\times10^{3}$	$1.33\pm0.96\times10^{2}$	$1.67\pm0.15\times10^{2}$	$2.00\pm0.00\times10^{1}$	0	0
			Pigmented	$2.70\pm0.30\times10^{2}$	$4.03\pm0.55\times10^{3}$	$1.33\pm0.96\times10^{2}$	$1.63\pm0.20\times10^{2}$	$2.00\pm0.00\times10^{1}$	0	0
			AAP	0	0	0	0	0	0	0
		Potato	Total	$4.27\pm0.50\times10^{3}$	$1.89\pm0.15\times10^{4}$	$5.10\pm0.99\times10^{3}$	$1.06\pm0.15\times10^{3}$	$7.53\pm1.10\times10^{3}$	$8.77\pm0.20\times10^{2}$	$1.29\pm0.13\times10^{3}$
			Pigmented	$1.97\pm0.35\times10^{3}$	$5.87\pm0.40\times10^{3}$	$2.70\pm0.74\times10^{3}$	$4.77\pm0.70\times10^{2}$	$1.40\pm0.65\times10^{3}$	$1.40\pm0.10\times10^{2}$	$2.40\pm0.40\times10^{2}$
			AAP	8.55×10^{1}	4.51×10^{2}	0	0	0	0	0
		BG-11	Total	$4.13\pm0.40\times10^{4}$	$1.26\pm0.08\times10^{5}$	$2.90\pm0.08\times10^{4}$	$8.17\pm0.75\times10^{2}$	$1.67\pm0.90\times10^{2}$	$1.30\pm0.15\times10^{2}$	$1.38\pm1.08\times10^{4}$
			AOP	$7.33\pm0.00\times10^{2}$	$1.70\pm0.05\times10^{3}$	$2.67\pm0.09\times10^{1}$	$1.00\pm0.50\times10^{1}$	0	$3.3\pm0.00\times10^{0}$	$1.33\pm1.00\times10^{1}$
		PNSm	Total	$8.67\pm1.50\times10^{1}$	$1.52\pm1.73\times10^{4}$	$1.33\pm0.47\times10^{1}$	$1.33\pm0.50\times10^{1}$	$1.20\pm0.30\times10^{2}$	$1.67\pm1.50\times10^{1}$	$1.67\pm1.50\times10^{1}$
			AnAnP	0	$2.67\pm0.00\times10^{1}$	0	0	0	0	0
2018	**Winter**	Alternative Sites				W10	**W1**	W9	**W6**	**W8**
		RO	Total	N/A	N/A	$8.20\pm0.60\times10^{2}$	$4.03\pm0.21\times10^{2}$	$8.63\pm0.90\times10^{2}$	$1.09\pm0.04\times10^{3}$	$1.53\pm0.05\times10^{2}$
			Pigmented	N/A	N/A	$4.43\pm2.15\times10^{2}$	$2.47\pm0.47\times10^{2}$	$5.57\pm0.05\times10^{2}$	$6.80\pm0.10\times10^{2}$	$7.00\pm0.5\times10^{1}$
			AAP	N/A	N/A	0	0	0	0	1.48×10^{1}
		OM	Total	N/A	N/A	$1.54\pm0.06\times10^{3}$	$6.65\pm1.15\times10^{2}$	$1.64\pm0.00\times10^{3}$	$1.52\pm0.19\times10^{3}$	$3.70\pm0.55\times10^{2}$
			Pigmented	N/A	N/A	$8.50\pm0.15\times10^{2}$	$3.15\pm0.15\times10^{2}$	$9.20\pm0.00\times10^{2}$	$3.00\pm0.60\times10^{2}$	$8.00\pm1.50\times10^{1}$
			AAP	N/A	N/A	0	0	0	0	0
		RO$_T$	Total	N/A	N/A	$6.67\pm0.50\times10^{1}$	$6.67\pm9.43\times10^{0}$	$7.00\pm1.50\times10^{1}$	$5.00\pm1.00\times10^{1}$	$6.67\pm5.00\times10^{0}$
			Pigmented	N/A	N/A	$4.67\pm0.00\times10^{1}$	$6.67\pm9.43\times10^{0}$	$6.67\pm1.50\times10^{1}$	$3.00\pm4.00\times10^{1}$	$6.67\pm5.00\times10^{0}$
			AAP	N/A	N/A	0	0	0	0	0
		Potato	Total	N/A	N/A	$7.90\pm0.20\times10^{2}$	N/A	$7.75\pm1.15\times10^{2}$	N/A	$1.77\pm0.45\times10^{2}$
			Pigmented	N/A	N/A	$6.27\pm0.50\times10^{2}$	N/A	$7.05\pm1.35\times10^{2}$	N/A	$7.00\pm2.50\times10^{1}$
			AAP	N/A	N/A	0	0	0	0	0
		BG-11	Total	N/A	N/A	$1.79\pm0.03\times10^{3}$	$4.60\pm4.81\times10^{2}$	$1.49\pm0.32\times10^{3}$	$1.85\pm1.22\times10^{3}$	$7.27\pm2.20\times10^{2}$
			AOP	N/A	N/A	0	0	0	0	0
		PNSm	Total	N/A	N/A	0	0	$0.67\pm1.00\times10^{1}$	$3.00\pm0.50\times10^{1}$	$3.33\pm5.00\times10^{0}$
			AnAnP	N/A	N/A	0	0	0	0	0

Table S4. Continued (6/7).

				5		6	7	8	9	10
Year	**Season**	Medium		liquid	sediment	liquid	liquid	liquid	liquid	liquid
2018	**Spring**	RO	Total	5.93±0.90×10^{2}	5.07±0.30×10^{4}	9.93±0.81×10^{2}	3.63±1.10×10^{3}	9.27±1.50×10^{2}	1.10±0.24×10^{3}	7.00±0.00×10^{2}
			Pigmented	2.50±0.20×10^{3}	1.60±0.15×10^{4}	3.67±0.21×10^{2}	1.97±0.60×10^{3}	2.97±0.25×10^{2}	5.47±0.55×10^{2}	2.50±0.00×10^{2}
			AAP	3.91×10^{2}	2.46×10^{3}	0	5.46×10^{1}	0	4.56×10^{1}	1.67×10^{1}
		OM	Total	1.08±0.50×10^{3}	1.22±0.43×10^{5}	3.75±0.26×10^{3}	9.30±0.15×10^{3}	3.77±0.90×10^{3}	6.37±1.10×10^{3}	6.67±0.45×10^{3}
			Pigmented	1.77±0.50×10^{3}	1.77±1.15×10^{4}	4.47±0.54×10^{2}	1.70±0.10×10^{3}	1.13±0.30×10^{3}	7.00±1.00×10^{2}	6.63±2.00×10^{2}
			AAP	0	0	0	5.15×10^{1}	0	0	4.87×10^{1}
		RO$_T$	Total	1.47±0.10×10^{2}	2.77±0.30×10^{3}	1.67±0.94×10^{1}	3.33±2.00×10^{1}	2.00±1.50×10^{1}	0	0.67±1.00×10^{1}
			Pigmented	1.10±0.15×10^{2}	2.50±0.10×10^{3}	1.33±1.25×10^{1}	3.33±2.00×10^{1}	2.00±1.50×10^{1}	0	0.67±1.00×10^{1}
			AAP	0	8.33×10^{2}	0	0	0	0	0
		Potato	Total	1.09±0.23×10^{3}	7.93±2.65×10^{3}	2.43±0.61×10^{2}	3.07±0.30×10^{2}	2.07±0.25×10^{2}	3.00±1.50×10^{1}	2.33±1.00×10^{1}
			Pigmented	5.13±2.40×10^{2}	1.20±0.40×10^{3}	1.30±0.36×10^{2}	1.87±0.20×10^{2}	2.33±1.50×10^{1}	1.67±1.00×10^{1}	1.00±0.50×10^{1}
			AAP	1.12×10^{2}	2.18×10^{2}	0	0	0	0	0
		BG-11	Total	1.45±0.08×10^{4}	4.47±0.15×10^{5}	2.15±0.16×10^{3}	1.97±0.05×10^{3}	1.59±0.012×10^{3}	5.23±1.15×10^{3}	1.67±0.50×10^{2}
			AOP	5.33±0.50×10^{1}	3.33±5.00×10^{2}	1.53±0.18×10^{3}	9.00±5.00×10^{1}	1.90±0.65×10^{2}	0	0
		PNSm	Total	3.33±2.50×10^{1}	1.16±0.01×10^{3}	5.87±1.66×10^{2}	3.00±1.00×10^{1}	9.33±4.50×10^{1}	2.67±0.00×10^{1}	5.00±1.50×10^{1}
			AnAnP	0	0	0	0	0	0	0
	**Summer**	RO	Total	1.55±0.12×10^{4}	5.67±0.40×10^{4}	8.67±0.94×10^{1}	4.50±0.00×10^{1}	1.70±0.10×10^{2}	3.15±0.00×10^{2}	2.17±0.00×10^{3}
			Pigmented	8.23±2.00×10^{3}	2.87±1.00×10^{4}	0	5.00±0.00×10^{0}	2.67±0.00×10^{1}	1.45±0.00×10^{2}	3.30±0.00×10^{2}
			AAP	0	0	0	0	0	0	0
		OM	Total	2.34±0.05×10^{4}	1.45±0.21×10^{5}	3.27±0.85×10^{2}	8.77±1.40×10^{2}	3.97±0.30×10^{2}	1.25±0.13×10^{3}	2.70±0.79×10^{3}
			Pigmented	6.13±0.90×10^{3}	3.00±1.00×10^{4}	5.33±2.87×10^{1}	6.67±0.50×10^{1}	1.00±0.35×10^{2}	1.77±0.10×10^{2}	4.50±2.80×10^{2}
			AAP	0	0	0	0	0	0	0
		RO$_T$	Total	1.08±0.02×10^{3}	1.16±0.04×10^{4}	3.33±4.71×10^{0}	3.30±5.00×10^{0}	6.67±0.00×10^{0}	1.33±1.00×10^{1}	2.00±2.00×10^{1}
			Pigmented	9.37±0.60×10^{2}	9.77±1.10×10^{3}	3.33±4.71×10^{0}	3.33±5.00×10^{0}	6.67±0.00×10^{0}	0	2.00±2.00×10^{1}
			AAP	0	0	0	0	0	0	0
		Potato	Total	3.55±0.84×10^{3}	8.73±1.35×10^{3}	3.33±1.70×10^{1}	2.33±0.50×10^{1}	3.67±1.00×10^{1}	1.33±1.00×10^{1}	1.49±0.01×10^{3}
			Pigmented	2.89±0.71×10^{3}	4.80±0.75×10^{2}	0	0	3.33±5.00×10^{0}	0	1.20±0.15×10^{2}
			AAP	0	0	0	0	0	0	0
		BG-11	Total	1.33±2.00×10^{7}	N/A	9.00±4.20×10^{2}	2.47±2.45×10^{3}	1.77±1.00×10^{2}	8.67±1.50×10^{1}	1.68±0.05×10^{3}
			AOP	2.77±0.25×10^{2}	1.47±0.25×10^{3}	0	2.00±0.50×10^{1}	0	0	0
		PNSm	Total	6.53±0.35×10^{2}	4.83±0.30×10^{2}	0	6.67±5.00×10^{0}	6.67±5.00×10^{0}	0.67±1.00×10^{1}	1.61±0.29×10^{3}
			AnAnP	0	0	0	0	0	0	0
	**Fall**	RO	Total	3.87±1.15×10^{3}	2.27±0.10×10^{4}	5.07±0.63×10^{3}	N/A	1.18±0.06×10^{4}	2.34±0.06×10^{3}	N/A
			Pigmented	1.33±0.10×10^{3}	4.33±1.50×10^{3}	4.03±0.69×10^{3}	N/A	4.00±0.50×10^{3}	6.47±0.00×10^{2}	N/A
			AAP	1.98×10^{2}	0	8.58×10^{1}	N/A	0	0	N/A
		OM	Total	8.07±1.20×10^{3}	7.53±0.00×10^{4}	9.57±1.18×10^{3}	N/A	5.73±0.90×10^{4}	1.05±0.17×10^{4}	N/A
			Pigmented	2.03±0.15×10^{3}	1.10±0.00×10^{4}	3.00±2.41×10^{3}	N/A	1.80±1.20×10^{4}	3.03±0.00×10^{3}	N/A
			AAP	0	0	0	N/A	3.91×10^{2}	0	N/A
		RO$_T$	Total	3.43±0.65×10^{2}	2.68±0.51×10^{3}	1.84±0.36×10^{3}	N/A	2.33±0.50×10^{1}	3.33±1.50×10^{1}	N/A
			Pigmented	3.30±0.65×10^{2}	1.51±0.32×10^{3}	1.84±0.36×10^{3}	N/A	2.33±0.50×10^{1}	3.33±1.50×10^{1}	N/A
			AAP	0	0	0	N/A	0	0	N/A
		Potato	Total	2.03±0.25×10^{3}	1.05±0.27×10^{4}	3.60±0.29×10^{3}	N/A	6.83±0.15×10^{2}	7.17±0.00×10^{2}	N/A
			Pigmented	7.03±0.30×10^{2}	1.47±0.35×10^{3}	3.03±0.37×10^{3}	N/A	8.67±2.00×10^{1}	2.83±0.40×10^{2}	N/A
			AAP	0	0	0	N/A	6.19×10^{0}	0	N/A
		BG-11	Total	3.25±0.65×10^{4}	2.02±0.22×10^{5}	2.09±0.20×10^{4}	N/A	N/A	N/A	N/A
			AOP	1.87±0.35×10^{3}	9.60±0.00×10^{3}	1.67±0.47×10^{1}	N/A	4.00±0.00×10^{1}	2.00±0.00×10^{1}	N/A
		PNSm	Total	1.47±0.00×10^{2}	2.54±0.13×10^{3}	5.67±0.47×10^{1}	N/A	2.00±0.50×10^{1}	1.00±1.00×10^{1}	N/A
			AnAnP	0	3.33±5.00×10^{0}	2.00±1.41×10^{1}	N/A	0	0	N/A
2019	**Winter**	Alternative Sites				W10	**W1**	W9	**W6**	**W8**
		RO	Total	N/A	N/A	3.83±0.80×10^{2}	3.30±1.27×10^{2}	5.10±0.10×10^{2}	3.00±0.40×10^{2}	3.87±0.15×10^{2}
			Pigmented	N/A	N/A	1.50±0.60×10^{2}	1.37±0.97×10^{2}	1.50±0.05×10^{2}	1.20±0.15×10^{2}	1.47±0.05×10^{2}
			AAP	N/A	N/A	0	0	0	0	0
		OM	Total	N/A	N/A	4.90±0.20×10^{2}	2.83±1.23×10^{2}	6.27±0.75×10^{2}	2.10±0.05×10^{2}	3.93±0.15×10^{2}
			Pigmented	N/A	N/A	1.33±0.35×10^{2}	2.00±1.63×10^{1}	1.70±0.35×10^{2}	2.67±0.05×10^{1}	8.67±1.00×10^{1}
			AAP	N/A	N/A	0	0	0	0	0
		RO$_T$	Total	N/A	N/A	1.50±0.50×10^{1}	0	3.33±5.00×10^{0}	1.67±2.50×10^{1}	3.33±5.00×10^{0}
			Pigmented	N/A	N/A	1.50±0.50×10^{1}	0	3.33±5.00×10^{0}	0.66±1.00×10^{1}	3.33±5.00×10^{0}
			AAP	N/A	N/A	0	0	0	0	0
		Potato	Total	N/A	N/A	3.20±1.20×10^{2}	2.37±1.03×10^{2}	3.90±0.20×10^{2}	1.27±0.20×10^{2}	3.10±0.40×10^{2}
			Pigmented	N/A	N/A	1.50±0.50×10^{2}	1.27±0.56×10^{2}	2.10±0.20×10^{2}	5.33±2.00×10^{1}	1.80±0.00×10^{2}
			AAP	N/A	N/A	0	0	0	0	0
		R2A	Total	N/A	N/A	N/A	5.40±2.79×10^{2}	N/A	5.40±0.90×10^{2}	N/A
			Pigmented	N/A	N/A	N/A	2.50±1.10×10^{2}	N/A	2.50±0.60×10^{2}	N/A
			AAP	N/A	N/A	N/A	0	N/A	0	N/A
		BG-11	Total	N/A	N/A	4.63±0.10×10^{2}	4.77±0.73×10^{2}	5.97±0.25×10^{2}	2.08±0.79×10^{3}	5.43±0.05×10^{2}
			AOP	N/A	N/A	0	3.33±4.71×10^{0}	0	0	0
		PNSm	Total	N/A	N/A	0	0	0	0	0
			AnAnP	N/A	N/A	0	0	0	0	0

Table S4. Continued (7/7).

Year	Season	Medium		5 liquid	5 sediment	6 liquid	7 liquid	8 liquid	9 liquid	10 liquid
2019	**Spring**	RO	Total	$4.80\pm0.35\times10^{4}$	$9.33\pm0.30\times10^{3}$	$1.51\pm0.07\times10^{5}$	$7.60\pm0.45\times10^{3}$	$3.56\pm1.28\times10^{3}$	$9.77\pm1.60\times10^{3}$	$5.87\pm0.05\times10^{2}$
			Pigmented	$1.17\pm0.20\times10^{4}$	$2.70\pm0.50\times10^{3}$	$1.29\pm0.20\times10^{5}$	$7.27\pm0.40\times10^{3}$	$2.81\pm0.87\times10^{3}$	$4.47\pm0.25\times10^{3}$	$4.30\pm0.85\times10^{2}$
			AAP	7.07×10^{2}	0	2.52×10^{3}	8.38×10^{2}	1.59×10^{2}	5.32×10^{2}	1.72×10^{1}
		OM	Total	$7.73\pm0.25\times10^{4}$	$3.53\pm0.50\times10^{4}$	$2.30\pm0.78\times10^{4}$	$1.16\pm0.04\times10^{4}$	$2.14\pm0.08\times10^{4}$	$1.18\pm0.16\times10^{4}$	$5.13\pm0.65\times10^{3}$
			Pigmented	$4.00\pm2.50\times10^{3}$	$7.67\pm3.00\times10^{3}$	$1.23\pm0.39\times10^{4}$	$8.20\pm0.50\times10^{3}$	$9.70\pm0.60\times10^{3}$	$3.80\pm0.45\times10^{3}$	$6.60\pm0.10\times10^{2}$
			AAP	2.22×10^{2}	1.21×10^{3}	0	5.13×10^{2}	1.01×10^{3}	7.04×10^{2}	0
		RO_T	Total	$2.20\pm0.25\times10^{3}$	$1.79\pm0.20\times10^{3}$	$6.07\pm0.29\times10^{3}$	$2.24\pm0.16\times10^{3}$	$1.97\pm0.25\times10^{3}$	$9.03\pm0.05\times10^{2}$	$8.67\pm2.00\times10^{1}$
			Pigmented	$2.13\pm0.18\times10^{3}$	$1.58\pm0.42\times10^{3}$	$6.07\pm0.29\times10^{3}$	$2.20\pm0.13\times10^{3}$	$1.93\pm0.26\times10^{3}$	$8.47\pm0.00\times10^{2}$	$8.67\pm2.00\times10^{1}$
			AAP	0	5.91×10^{2}	0	0	0	0	0
		Potato	Total	$2.93\pm0.85\times10^{4}$	$5.37\pm0.05\times10^{3}$	$1.00\pm0.13\times10^{4}$	$3.67\pm0.25\times10^{3}$	$1.00\pm0.00\times10^{4}$	$2.13\pm0.03\times10^{3}$	$1.21\pm0.13\times10^{3}$
			Pigmented	$1.30\pm0.45\times10^{4}$	$1.73\pm0.20\times10^{3}$	$8.77\pm0.87\times10^{3}$	$3.43\pm0.30\times10^{3}$	$1.00\pm0.00\times10^{4}$	$6.33\pm9.50\times10^{2}$	$7.80\pm1.45\times10^{2}$
			AAP	7.65×10^{2}	3.47×10^{2}	1.95×10^{3}	4.29×10^{2}	0	3.17×10^{1}	4.88×10^{1}
		R2A	Total	$8.40\pm2.45\times10^{4}$	$3.67\pm0.25\times10^{4}$	$1.91\pm0.10\times10^{4}$	N/A	$1.49\pm0.01\times10^{4}$	$1.11\pm0.07\times10^{4}$	$6.50\pm1.05\times10^{3}$
			Pigmented	$2.33\pm0.70\times10^{4}$	$1.23\pm0.20\times10^{4}$	$9.13\pm6.46\times10^{3}$	N/A	$8.40\pm0.50\times10^{3}$	$5.43\pm0.20\times10^{3}$	$2.80\pm0.45\times10^{3}$
			AAP	0	3.36×10^{3}	4.35×10^{2}	N/A	1.80×10^{3}	0	1.08×10^{2}
		BG-11	Total	$8.33\pm0.85\times10^{4}$	$3.09\pm0.44\times10^{4}$	$1.78\pm0.26\times10^{4}$	$5.35\pm0.00\times10^{4}$	$1.78\pm0.07\times10^{4}$	$1.29\pm0.26\times10^{4}$	$2.41\pm0.45\times10^{4}$
			AOP	0	$3.33\pm5.00\times10^{0}$	$1.33\pm1.25\times10^{1}$	0	0	$6.67\pm1.00\times10^{1}$	0
		PNSm	Total	$9.37\pm0.25\times10^{3}$	$1.05\pm0.01\times10^{4}$	$1.33\pm1.25\times10^{1}$	$2.33\pm2.00\times10^{1}$	$3.33\pm0.00\times10^{0}$	$1.33\pm0.05\times10^{1}$	$0.67\pm1.00\times10^{1}$
			AnAnP	0	0	0	0	0	0	0
	**Summer**	RO	Total	$7.40\pm0.10\times10^{2}$	$5.45\pm0.00\times10^{5}$	$2.85\pm0.06\times10^{3}$	$2.83\pm0.03\times10^{3}$	$2.63\pm0.25\times10^{3}$	$3.24\pm1.07\times10^{3}$	$7.77\pm1.05\times10^{3}$
			Pigmented	$1.13\pm0.20\times10^{2}$	$1.30\pm0.00\times10^{5}$	$1.23\pm0.06\times10^{3}$	$1.08\pm0.14\times10^{3}$	$8.33\pm2.50\times10^{2}$	$6.47\pm1.30\times10^{2}$	$4.13\pm0.20\times10^{3}$
			AAP	0	1.34×10^{4}	0	0	0	0	1.80×10^{2}
		OM	Total	$1.85\pm0.00\times10^{3}$	$6.10\pm1.85\times10^{5}$	$4.50\pm0.80\times10^{3}$	$5.33\pm0.75\times10^{3}$	$1.83\pm0.18\times10^{4}$	$5.30\pm0.45\times10^{3}$	$1.41\pm0.00\times10^{4}$
			Pigmented	$2.10\pm0.00\times10^{2}$	$8.00\pm1.00\times10^{4}$	$2.33\pm1.25\times10^{2}$	$2.67\pm0.00\times10^{2}$	$2.20\pm0.05\times10^{3}$	$1.77\pm0.00\times10^{3}$	$1.65\pm0.00\times10^{3}$
			AAP	0	3.08×10^{3}	8.33×10^{0}	9.52×10^{0}	0	0	1.10×10^{2}
		RO_T	Total	$4.00\pm0.50\times10^{1}$	$3.77\pm1.20\times10^{4}$	$3.50\pm1.61\times10^{2}$	$7.33\pm5.00\times10^{1}$	$4.33\pm1.00\times10^{1}$	$1.00\pm0.10\times10^{2}$	$3.00\pm0.50\times10^{1}$
			Pigmented	$3.33\pm0.00\times10^{1}$	$3.60\pm1.05\times10^{4}$	$3.50\pm1.61\times10^{2}$	$7.33\pm5.00\times10^{1}$	$4.33\pm1.00\times10^{1}$	$1.00\pm0.10\times10^{2}$	$3.00\pm0.50\times10^{1}$
			AAP	4.76×10^{0}	7.20×10^{3}	7.00×10^{1}	1.47×10^{1}	7.88×10^{0}	0	0
		Potato	Total	$4.07\pm0.75\times10^{2}$	$4.23\pm0.30\times10^{4}$	$9.60\pm0.78\times10^{3}$	$1.35\pm0.06\times10^{3}$	$7.07\pm0.35\times10^{3}$	$8.00\pm0.00\times10^{3}$	$2.40\pm0.00\times10^{3}$
			Pigmented	$9.67\pm0.50\times10^{1}$	$1.77\pm0.10\times10^{4}$	$2.03\pm0.24\times10^{3}$	$8.13\pm0.25\times10^{2}$	$3.33\pm0.15\times10^{3}$	$1.20\pm0.00\times10^{3}$	$1.91\pm0.00\times10^{3}$
			AAP	1.76×10^{1}	2.52×10^{3}	1.45×10^{2}	4.78×10^{1}	1.52×10^{2}	0	1.12×10^{2}
		R2A	Total	$1.54\pm0.36\times10^{3}$	$1.90\pm0.29\times10^{5}$	$1.45\pm0.12\times10^{3}$	$1.86\pm0.08\times10^{3}$	$5.43\pm0.90\times10^{3}$	$1.10\pm0.26\times10^{4}$	$5.57\pm1.20\times10^{3}$
			Pigmented	$2.27\pm0.30\times10^{2}$	$2.57\pm0.25\times10^{4}$	$7.67\pm0.63\times10^{2}$	$7.53\pm0.20\times10^{2}$	$1.53\pm0.20\times10^{3}$	$1.47\pm0.30\times10^{3}$	$2.77\pm0.30\times10^{3}$
			AAP	0	2.14×10^{3}	3.65×10^{1}	3.42×10^{1}	1.92×10^{2}	0	3.95×10^{2}
		BG-11	Total	$3.07\pm0.10\times10^{4}$	$1.22\pm0.01\times10^{6}$	$7.73\pm0.46\times10^{3}$	$4.53\pm0.05\times10^{3}$	$5.43\pm1.85\times10^{3}$	$1.22\pm0.71\times10^{4}$	$6.23\pm0.10\times10^{3}$
			AOP	$1.27\pm0.13\times10^{3}$	$2.97\pm0.03\times10^{3}$	$1.67\pm0.47\times10^{1}$	$2.33\pm0.50\times10^{1}$	$6.33\pm2.50\times10^{1}$	$5.37\pm1.55\times10^{2}$	$2.40\pm1.20\times10^{2}$
		PNSm	Total	$4.53\pm0.10\times10^{3}$	$1.12\pm0.06\times10^{4}$	$8.87\pm2.19\times10^{2}$	$1.23\pm0.15\times10^{3}$	$3.33\pm0.00\times10^{1}$	$7.90\pm0.10\times10^{2}$	$3.67\pm1.50\times10^{1}$
			AnAnP	$7.33\pm2.00\times10^{2}$	0	$7.67\pm2.96\times10^{2}$	$5.40\pm0.40\times10^{2}$	0	0	0
	**Fall**	RO	Total	$2.06\pm0.11\times10^{4}$	$1.72\pm0.47\times10^{5}$	$1.47\pm0.03\times10^{4}$	$1.78\pm0.07\times10^{3}$	$1.66\pm0.14\times10^{4}$	$5.80\pm1.70\times10^{3}$	$6.13\pm0.35\times10^{4}$
			Pigmented	$4.17\pm0.35\times10^{3}$	$5.53\pm0.80\times10^{4}$	$1.03\pm0.05\times10^{4}$	$9.47\pm0.40\times10^{2}$	$8.57\pm1.40\times10^{3}$	$3.00\pm0.60\times10^{3}$	$1.27\pm0.95\times10^{4}$
			AAP	0	0	3.21×10^{2}	0	0	0	2.88×10^{2}
		OM	Total	$5.73\pm0.15\times10^{4}$	$9.07\pm0.50\times10^{5}$	$1.80\pm0.10\times10^{4}$	$7.97\pm3.50\times10^{3}$	$1.62\pm0.33\times10^{4}$	$9.17\pm0.15\times10^{3}$	$4.70\pm3.80\times10^{4}$
			Pigmented	$7.00\pm1.00\times10^{3}$	$1.17\pm0.05\times10^{5}$	$1.25\pm0.15\times10^{4}$	$1.57\pm0.15\times10^{3}$	$5.60\pm1.20\times10^{3}$	$2.43\pm0.15\times10^{3}$	$7.33\pm3.50\times10^{3}$
			AAP	0	5.83×10^{3}	2.27×10^{2}	0	0	0	8.84×10^{1}
		RO_T	Total	$3.23\pm0.65\times10^{3}$	$5.03\pm0.80\times10^{4}$	$2.60\pm1.10\times10^{3}$	$3.73\pm0.25\times10^{2}$	$2.47\pm0.40\times10^{2}$	$1.13\pm0.71\times10^{3}$	$7.07\pm0.85\times10^{2}$
			Pigmented	$3.00\pm0.60\times10^{3}$	$4.97\pm0.70\times10^{4}$	$2.60\pm1.10\times10^{3}$	$3.63\pm0.15\times10^{2}$	$2.40\pm0.35\times10^{2}$	$1.11\pm0.73\times10^{3}$	$7.07\pm0.85\times10^{2}$
			AAP	9.23×10^{2}	0	0	0	1.26×10^{1}	0	1.09×10^{2}
		Potato	Total	$5.73\pm2.65\times10^{3}$	$5.57\pm0.85\times10^{4}$	$1.43\pm0.07\times10^{4}$	$1.44\pm0.12\times10^{3}$	$8.47\pm0.65\times10^{2}$	$2.30\pm0.21\times10^{3}$	$3.90\pm0.50\times10^{3}$
			Pigmented	$2.47\pm0.20\times10^{3}$	$1.83\pm0.55\times10^{4}$	$1.03\pm0.08\times10^{4}$	$8.47\pm0.50\times10^{2}$	$5.00\pm0.90\times10^{2}$	$1.17\pm0.32\times10^{3}$	$1.60\pm0.25\times10^{3}$
			AAP	0	2.62×10^{3}	0	8.06×10^{1}	0	0	0
		R2A	Total	$1.60\pm0.15\times10^{4}$	$2.25\pm0.22\times10^{5}$	$2.60\pm1.10\times10^{3}$	$8.20\pm0.15\times10^{3}$	$7.63\pm0.70\times10^{3}$	$4.43\pm0.50\times10^{3}$	$1.61\pm0.30\times10^{4}$
			Pigmented	$2.97\pm1.25\times10^{3}$	$6.03\pm1.05\times10^{4}$	$2.60\pm1.10\times10^{3}$	$1.63\pm0.10\times10^{3}$	$1.93\pm0.10\times10^{3}$	$2.23\pm0.50\times10^{3}$	$2.23\pm0.85\times10^{3}$
			AAP	0	1.51×10^{4}	1.04×10^{2}	0	0	0	9.71×10^{1}
		BG-11	Total	$7.33\pm1.35\times10^{4}$	$9.67\pm1.60\times10^{5}$	$4.23\pm0.13\times10^{4}$	$5.37\pm1.90\times10^{2}$	$1.86\pm0.10\times10^{4}$	$2.17\pm0.15\times10^{4}$	$6.57\pm5.00\times10^{4}$
			AOP	$1.10\pm0.30\times10^{2}$	$4.50\pm0.00\times10^{3}$	0	0	$6.67\pm0.00\times10^{0}$	$8.67\pm0.50\times10^{1}$	$2.33\pm0.40\times10^{2}$
		PNSm	Total	$3.17\pm0.15\times10^{2}$	$4.63\pm1.33\times10^{3}$	$1.47\pm0.41\times10^{2}$	$6.33\pm4.50\times10^{1}$	$3.33\pm5.00\times10^{0}$	$1.33\pm1.00\times10^{1}$	$2.67\pm0.50\times10^{1}$
			AnAnP	0	0	0	0	0	0	0
2020	**Winter**	Alternative Sites				W10	**W1**	W9	**W6**	**W8**
			Total	N/A	N/A	$8.67\pm1.00\times10^{1}$	$1.93\pm0.31\times10^{2}$	$2.33\pm1.00\times10^{1}$	$1.20\pm0.05\times10^{2}$	$9.67\pm2.00\times10^{1}$
			Pigmented	N/A	N/A	0	$5.00\pm2.16\times10^{1}$	$1.33\pm1.00\times10^{1}$	$3.33\pm1.50\times10^{1}$	$2.33\pm0.00\times10^{1}$
			AAP	N/A	N/A	0	0	0	2.67×10^{0}	0
		OM	Total	N/A	N/A	$6.53\pm0.35\times10^{2}$	$7.00\pm0.00\times10^{1}$	$6.00\pm0.00\times10^{1}$	$2.70\pm0.55\times10^{2}$	$3.00\pm0.50\times10^{1}$
			Pigmented	N/A	N/A	0	$2.33\pm1.70\times10^{1}$	0	$2.67\pm2.50\times10^{1}$	0
			AAP	N/A	N/A	0	0	0	0	0
		RO_T	Total	N/A	N/A	$6.67\pm0.00\times10^{0}$	$6.33\pm1.25\times10^{1}$	$4.00\pm1.00\times10^{1}$	0	$1.00\pm0.50\times10^{1}$
			Pigmented	N/A	N/A	0	$6.67\pm4.71\times10^{0}$	0	$3.00\pm1.00\times10^{1}$	$1.00\pm0.50\times10^{1}$
			AAP	N/A	N/A	0	0	0	0	0
		Potato	Total	N/A	N/A	$6.67\pm0.00\times10^{0}$	$6.00\pm1.41\times10^{1}$	0	$3.00\pm1.50\times10^{1}$	$2.33\pm1.00\times10^{1}$
			Pigmented	N/A	N/A	0	$1.00\pm0.82\times10^{1}$	0	$3.33\pm0.00\times10^{0}$	0
			AAP	N/A	N/A	0	0	0	0	0
		R2A	Total	N/A	N/A	$2.90\pm1.10\times10^{2}$	$5.90\pm0.71\times10^{2}$	$1.27\pm0.20\times10^{2}$	$5.67\pm2.5\times10^{1}$	$1.27\pm0.05\times10^{2}$
			Pigmented	N/A	N/A	$5.67\pm2.00\times10^{1}$	0	$3.67\pm1.00\times10^{1}$	$6.67\pm0.00\times10^{0}$	$2.00\pm1.00\times10^{1}$
			AAP	N/A	N/A	0	0	0	0	1.13×10^{1}
		BG-11	Total	N/A	N/A	$1.28\pm0.31\times10^{3}$	$2.70\pm0.29\times10^{2}$	$4.17\pm0.55\times10^{2}$	$4.00\pm0.00\times10^{1}$	$6.00\pm3.00\times10^{1}$
			AOP	N/A	N/A	$1.33\pm0.50\times10^{1}$	$3.67\pm2.49\times10^{1}$	0	$3.33\pm5.00\times10^{0}$	0
		PNSm	Total	N/A	N/A	0	0	$6.00\pm0.00\times10^{1}$	0	0
			AnAnP	N/A	N/A	0	0	0	0	0

Table S5. AAP type species used as reference for eDNA 16S V4 rRNA gene analysis.

Phylum	Class	Order	Family	Genus	Species	Reference
Proteobacteria	*α-Proteobacteria*	*Sphingomonadales*	*Erythromonadaceae*	*Erythrobacter*	*litoralis*	[17]
Proteobacteria	*α-Proteobacteria*	*Sphingomonadales*	*Erythromonadaceae*	*Erythromicrobium*	*ramosum*	[17]
Proteobacteria	*α-Proteobacteria*	*Sphingomonadales*	*Erythromonadaceae*	*Porphyrobacter*	*sanguineus*	[17]
Proteobacteria	*α-Proteobacteria*	*Sphingomonadales*	*Sphingomonadaceae*	*Blastomonas*	*ursincola*	[17]
Proteobacteria	*α-Proteobacteria*	*Sphingomonadales*	*Sphingomonadaceae*	*Sphingomonas*	*kaistensis*	[71]
Proteobacteria	*α-Proteobacteria*	*Sphingomonadales*	*Sphingomonadaceae*	*Citromicrobium*	*bathyomarinum*	[70]
Proteobacteria	*α-Proteobacteria*	*Sphingomonadales*	*Sphingomonadaceae*	*Citrimicrobium*	*luteum*	[18]
Proteobacteria	*α-Proteobacteria*	*Sphingomonadales*	*Sphingosinicellaceae*	*Sandaracinobacter*	*sibiricus*	[17]
Proteobacteria	*α-Proteobacteria*	*Sphingomonadales*	*Sphingosinicellaceae*	*Sandarakinorhabdus*	*limnophila*	[71]
Proteobacteria	*α-Proteobacteria*	*Rhodobacterales*	*Rhodobacteraceae*	*Roseobacter*	*denitrificans*	[17]
Proteobacteria	*α-Proteobacteria*	*Rhodobacterales*	*Rhodobacteraceae*	*Roseovarius*	*tolerans*	[70]
Proteobacteria	*α-Proteobacteria*	*Rhodobacterales*	*Rhodobacteraceae*	*Roseibium*	*denhamense*	[70]
Proteobacteria	*α-Proteobacteria*	*Rhodobacterales*	*Rhodobacteraceae*	*Rubrimonas*	*cliftonensis*	[70]
Proteobacteria	*α-Proteobacteria*	*Rhodobacterales*	*Rhodobacteraceae*	*Roseivivax*	*halodurans*	[70]
Proteobacteria	*α-Proteobacteria*	*Rhodobacterales*	*Rhodobacteraceae*	*Sulfitobacter*	*guttiformis*	[70]
Proteobacteria	*α-Proteobacteria*	*Rhodobacterales*	*Rhodobacteraceae*	*Roseinatronobacter*	*thiooxidans*	[70]
Proteobacteria	*α-Proteobacteria*	*Rhodobacterales*	*Rhodobacteraceae*	*Roseicyclus*	*mahoneyensis*	[71]
Proteobacteria	*α-Proteobacteria*	*Rhodobacterales*	*Rhodobacteraceae*	*Thalassobacter*	*stenotrophicus*	[71]
Proteobacteria	*α-Proteobacteria*	*Rhodobacterales*	*Rhodobacteraceae*	*Dinoroseobacter*	*shibae*	[71]
Proteobacteria	*α-Proteobacteria*	*Rhodobacterales*	*Rhodobacteraceae*	*Roseibacterium*	*elongatum*	[71]
Proteobacteria	*α-Proteobacteria*	*Rhodobacterales*	*Rhodobacteraceae*	*Roseisalinus*	*antarcticus*	[71]
Proteobacteria	*α-Proteobacteria*	*Rhodobacterales*	*Rhodobacteraceae*	*Labrenzia*	*alexandrii*	[71]
Proteobacteria	*α-Proteobacteria*	*Rhodobacterales*	*Rhodobacteraceae*	*Roseibaca*	*ekhonensis*	[72]
Proteobacteria	*α-Proteobacteria*	*Rhodobacterales*	*Rhodobacteraceae*	*Roseicitereum*	*antarcticum*	[72]
Proteobacteria	*α-Proteobacteria*	*Rhodobacterales*	*Rhodobacteraceae*	*Charonomicrobium*	*ambiphotophicum*	[72]
Proteobacteria	*α-Proteobacteria*	*Rhodobacterales*	*Rhodobacteraceae*	*Roseibacula*	*alcaliiphilum*	[18]
Proteobacteria	*α-Proteobacteria*	*Rhodospirillales*	*Acetobacteraceae*	*Roseococcus*	*thiosulfatophilus*	[17]
Proteobacteria	*α-Proteobacteria*	*Rhodospirillales*	*Acetobacteraceae*	*Acidiphilium*	*rubrum*	[17]
Proteobacteria	*α-Proteobacteria*	*Rhodospirillales*	*Acetobacteraceae*	*Acidishaera*	*rubrifaceins*	[70]
Proteobacteria	*α-Proteobacteria*	*Rhodospirillales*	*Acetobacteraceae*	*Rubritepida*	*flocculans*	[70]
Proteobacteria	*α-Proteobacteria*	*Rhodospirillales*	*Acetobacteraceae*	*Craurococcus*	*roseus*	[70]
Proteobacteria	*α-Proteobacteria*	*Rhodospirillales*	*Acetobacteraceae*	*Paracraurococcus*	*ruber*	[70]
Proteobacteria	*α-Proteobacteria*	*Rhodospirillales*	*Acetobacteraceae*	*Geminicoccus*	*roseus*	[71]
Proteobacteria	*α-Proteobacteria*	*Rhodospirillales*	*Acetobacteraceae*	*Humitalea*	*rosea*	[18]
Proteobacteria	*α-Proteobacteria*	*Rhodospirillales*	*Acetobacteraceae*	*Roseomonas*	*aestuarii*	[72]
Proteobacteria	*α-Proteobacteria*	*Caulobacterales*	*Maricaulaceae*	*Alkalicaulis*	*satelles*	[68]
Proteobacteria	*α-Proteobacteria*	*Caulobacterales*	*Maricaulaceae*	*Photocaulis*	*sulfatitolerans*	[68]
Proteobacteria	*α-Proteobacteria*	*Rhodospirillales*	*Rhodospirillaceae*	*Aerophototrophica*	*crusticola*	[74]
Proteobacteria	*α-Proteobacteria*	*Hyphomicrobiales*	*Phyllobacteriaceae*	*Hoeflea*	*photoytophica*	[71]
Proteobacteria	*α-Proteobacteria*	*Hyphomicrobiales*	*Methylobacteriaceae*	*Methylorubrum*	*extorquens*	[73]
Proteobacteria	*β-Proteobacteria*	*Burkholderiales*	*Comamonadaceae*	*Limnohabitans*	*planktonicus*	[63]
Proteobacteria	*β-Proteobacteria*	*Burkholderiales*	*Comamonadaceae*	*Roseateles*	*depolymerans*	[70]
Proteobacteria	*β-Proteobacteria*	*Rhodocyclales*	*Rhodocyclaceae*	*Aquincola*	*tertiaricarbonis*	[20]
Proteobacteria	*γ-Proteobacteria*	*Cellvibrionales*	*Halieaceae*	*Congregibacter*	*litoralis*	[72]
Proteobacteria	*γ-Proteobacteria*	*Cellvibrionales*	*Halieaceae*	*Chromocurvus*	*halotolerans*	[72]
Gemmatimonadetes	*Gemmatimonadetes*	*Gemmatimonales*	*Gemmatimonaceae*	*Gemmatimonas*	*phototrophica*	[20]
Acidobacteriota	*"Acidobacteria"*	*Acidobacteriales*	*Acidobacteriaceae*	*Chloracidobacterium*	*thermophilum*	[69]

Table S6. AnAnP type species used as reference for eDNA 16S V4 rRNA gene analysis.

Phylum	Class	Order	Family	Genus	Species	Reference
Chloroflexi	*Chloroflexia*	*Chloroflexales*	*Chloroflexiaceae*	*Chloroflexus*	*aurantiacus*	[75]
Chloroflexi	*Chloroflexia*	*Chloroflexales*	*Chloroflexiaceae*	*Oscillochloris*	*trichoides*	[75]
Chloroflexi	*Chloroflexia*	*Chloroflexales*	*Chloroflexiaceae*	*Chloroploca*	*asiatica*	[75]
Chloroflexi	*Chloroflexia*	*Chloroflexales*	*Roseiflexiaceae*	*Roseiflexus*	*castenholzii*	[75]
Chloroflexi	*Chloroflexia*	*Chloroflexales*	*Kouleothrichniae*	*Kouleothrix*	*aurantiaca*	[75]
Firmicutes	*Clostridia*	*Eubacteriales*	*Heliobacteriaceae*	*Heliobacterium*	*chlorum*	[77]
Firmicutes	*Clostridia*	*Eubacteriales*	*Heliobacteriaceae*	*Heliomicrobium*	*gestii*	[77]
Firmicutes	*Clostridia*	*Eubacteriales*	*Heliobacteriaceae*	*Heliophilum*	*fasciatum*	[77]
Firmicutes	*Clostridia*	*Eubacteriales*	*Heliobacteriaceae*	*Heliorestis*	*daurensis*	[77]
Chlorobiota	*Chlorobia*	*Chlorobiales*	*Chlorobiaceae*	*Ancalochloris*	*perfilievii*	[78]
Chlorobiota	*Chlorobia*	*Chlorobiales*	*Chlorobiaceae*	*Chlorobaculum*	*tepidum*	[78]
Chlorobiota	*Chlorobia*	*Chlorobiales*	*Chlorobiaceae*	*Chlorobium*	*limicola*	[78]
Chlorobiota	*Chlorobia*	*Chlorobiales*	*Chlorobiaceae*	*Chloroherpeton*	*thalassium*	[78]
Chlorobiota	*Chlorobia*	*Chlorobiales*	*Chlorobiaceae*	*Prosthecochloris*	*aestuarii*	[78]
Proteobacteria	*α-Proteobacteria*	*Hyphomicrobiales*	*Blastochloridaceae*	*Blastochloris*	*viridis*	[75]
Proteobacteria	*α-Proteobacteria*	*Hyphomicrobiales*	*Hyphomicrobiaceae*	*Rhodomicrobium*	*vannielii*	[75]
Proteobacteria	*α-Proteobacteria*	*Hyphomicrobiales*	*Nitrobacteraceae*	*Rhodoplanes*	*roseus*	[75]
Proteobacteria	*Alphaproteobacteria*	*Hyphomicrobiales*	*Nitrobacteraceae*	*Rhodopseudomonas*	*palustris*	[92]
Proteobacteria	*α-Proteobacteria*	*Rhodospirillales*	*Rhodospirillaceae*	*Phaeospirillum*	*fulvum*	[75]
Proteobacteria	*α-Proteobacteria*	*Rhodospirillales*	*Rhodospirillaceae*	*Pararhodospirillum*	*photometricum*	[75]
Proteobacteria	*α-Proteobacteria*	*Rhodospirillales*	*Rhodospirillaceae*	*Rhodospirillum*	*rubrum*	[75]
Proteobacteria	*α-Proteobacteria*	*Rhodospirillales*	*Rhodospirillaceae*	*Roseospira*	*mediosalina*	[75]
Proteobacteria	*α-Proteobacteria*	*Rhodobacterales*	*Rhodobacteraceae*	*Planktomarina*	*temperata*	[75]
Proteobacteria	*α-Proteobacteria*	*Rhodobacterales*	*Rhodobacteraceae*	*Rhodobacter*	*capsulatus*	[75]
Proteobacteria	*α-Proteobacteria*	*Rhodobacterales*	*Rhodobacteraceae*	*Halovulum*	*dunhuangense*	[75]
Proteobacteria	*α-Proteobacteria*	*Rhodobacterales*	*Rhodobacteraceae*	*Cereibacter*	*changlensis*	[75]
Proteobacteria	*β-Proteobacteria*	*Rhodocyclales*	*Rhodocyclaceae*	*Rhodocyclus*	*purpureus*	[75]
Proteobacteria	*β-Proteobacteria*	*Burkholderiales*	*Comamonadaceae*	*Rubrivivax*	*gelatinosus*	[75]
Proteobacteria	*β-Proteobacteria*	*Burkholderiales*	*Comamonadaceae*	*Hydrogenophaga*	*flava*	[75]
Proteobacteria	*β-Proteobacteria*	*Burkholderiales*	*Comamonadaceae*	*Rhodoferax*	*fermentans*	[75]
Proteobacteria	*γ-Proteobacteria*	*Chromatiales*	*Ectothiorhodospiraceae*	*Ectothiorhodosinus*	*mongolicus*	[76]
Proteobacteria	*γ-Proteobacteria*	*Chromatiales*	*Ectothiorhodospiraceae*	*Ectothiorhodospira*	*mobilis*	[76]
Proteobacteria	*γ-Proteobacteria*	*Chromatiales*	*Chromatiaceae*	*Allochromatium*	*vinosum*	[76]
Proteobacteria	*γ-Proteobacteria*	*Chromatiales*	*Chromatiaceae*	*Caldichromatium*	*japonicum*	[79]
Proteobacteria	*γ-Proteobacteria*	*Chromatiales*	*Chromatiaceae*	*Chromatium*	*okenii*	[76]
Proteobacteria	*γ-Proteobacteria*	*Chromatiales*	*Chromatiaceae*	*Halochromatium*	*salexigens*	[76]
Proteobacteria	*γ-Proteobacteria*	*Chromatiales*	*Chromatiaceae*	*Imhoffiella*	*purpurea*	[80]
Proteobacteria	*γ-Proteobacteria*	*Chromatiales*	*Chromatiaceae*	*Isochromatium*	*buderi*	[81]
Proteobacteria	*γ-Proteobacteria*	*Chromatiales*	*Chromatiaceae*	*Lamprobacter*	*modestohalophilus*	[82]
Proteobacteria	*γ-Proteobacteria*	*Chromatiales*	*Chromatiaceae*	*Lamprocystis*	*roseopersicina*	[75]
Proteobacteria	*γ-Proteobacteria*	*Chromatiales*	*Chromatiaceae*	*Marichromatium*	*gracile*	[76]
Proteobacteria	*γ-Proteobacteria*	*Chromatiales*	*Chromatiaceae*	*Nitrosococcus*	*nitrosus*	[83]
Proteobacteria	*γ-Proteobacteria*	*Chromatiales*	*Chromatiaceae*	*Phaeobacterium*	*nitratireducens*	[84]
Proteobacteria	*γ-Proteobacteria*	*Chromatiales*	*Chromatiaceae*	*Phaeochromatium*	*fluminis*	[85]
Proteobacteria	*γ-Proteobacteria*	*Chromatiales*	*Chromatiaceae*	*Rhabdochromatium*	*marinum*	[86]
Proteobacteria	*γ-Proteobacteria*	*Chromatiales*	*Chromatiaceae*	*Thermochromatium*	*tepidum*	[75]
Proteobacteria	*γ-Proteobacteria*	*Chromatiales*	*Chromatiaceae*	*Thioalkalicoccus*	*limnaeus*	[87]
Proteobacteria	*γ-Proteobacteria*	*Chromatiales*	*Chromatiaceae*	*Thiobaca*	*trueperi*	[88]
Proteobacteria	*γ-Proteobacteria*	*Chromatiales*	*Chromatiaceae*	*Thiocapsa*	*roseopersicina*	[75]
Proteobacteria	*γ-Proteobacteria*	*Chromatiales*	*Chromatiaceae*	*Thiococcus*	*pfennigii*	[75]
Proteobacteria	*γ-Proteobacteria*	*Chromatiales*	*Chromatiaceae*	*Thiocystis*	*violacea*	[75]
Proteobacteria	*γ-Proteobacteria*	*Chromatiales*	*Chromatiaceae*	*Thiodictyon*	*elegans*	[89]
Proteobacteria	*γ-Proteobacteria*	*Chromatiales*	*Chromatiaceae*	*Thiohalocapsa*	*halophila*	[75]
Proteobacteria	*γ-Proteobacteria*	*Chromatiales*	*Chromatiaceae*	*Thiolamprovum*	*pedioforme*	[75]
Proteobacteria	*γ-Proteobacteria*	*Chromatiales*	*Chromatiaceae*	*Thiopedia*	*rosea*	[90]
Proteobacteria	*γ-Proteobacteria*	*Chromatiales*	*Chromatiaceae*	*Thiophaeococcus*	*mangrovi*	[75]
Proteobacteria	*γ-Proteobacteria*	*Chromatiales*	*Chromatiaceae*	*Thiorhodococcus*	*minor*	[75]
Proteobacteria	*γ-Proteobacteria*	*Chromatiales*	*Chromatiaceae*	*Thiorhodovibrio*	*winogradskyi*	[75]
Proteobacteria	*γ-Proteobacteria*	*Chromatiales*	*Chromatiaceae*	*Thiospirillum*	*jenense*	[91]
Proteobacteria	*γ-Proteobacteria*	*Chromatiales*	*Halorhodospiraceae*	*Halochlorospira*	*halochloris*	[76]
Proteobacteria	*γ-Proteobacteria*	*Chromatiales*	*Halorhodospiraceae*	*Halorhodospira*	*halophila*	[76]
